# Structural Characterization of Maitotoxins Produced by Toxic *Gambierdiscus* Species

**DOI:** 10.3390/md20070453

**Published:** 2022-07-12

**Authors:** J. Sam Murray, Sarah C. Finch, Elizabeth M. Mudge, Alistair L. Wilkins, Jonathan Puddick, D. Tim Harwood, Lesley L. Rhodes, Roel van Ginkel, Frode Rise, Michèle R. Prinsep

**Affiliations:** 1 Cawthron Institute, Private Bag 2, Nelson 7040, New Zealand; jonathan.puddick@cawthron.org.nz (J.P.); tim.harwood@cawthron.org.nz (D.T.H.); lesley.rhodes@cawthron.org.nz (L.L.R.); roel.vanginkel@cawthron.org.nz (R.v.G.); 2 New Zealand Food Safety Science and Research Centre, Massey University, Private Bag 11 222, Palmerston North 4442, New Zealand; 3 School of Science, University of Waikato, Private Bag 3105, Hamilton 3240, New Zealand; wilkinsalw@hotmail.com (A.L.W.); michele.prinsep@waikato.ac.nz (M.R.P.); 4 AgResearch, Ruakura Research Centre, Private Bag 3123, Hamilton 3240, New Zealand; sarah.finch@agresearch.co.nz; 5 Biotoxin Metrology, National Research Council Canada, 1411 Oxford Street, Halifax, NS B3H 3Z1, Canada; elizabeth.mudge@nrc-cnrc.gc.ca; 6 Department of Chemistry, University of Oslo, Blindern, P.O. Box 1033, NO-0315 Oslo, Norway; frode.rise@kjemi.uio.no

**Keywords:** ciguatera poisoning, mass spectrometry, nuclear magnetic resonance spectroscopy, dinoflagellate, benthic, bioassay, acute toxicity

## Abstract

Identifying compounds responsible for the observed toxicity of the *Gambierdiscus* species is a critical step to ascertaining whether they contribute to ciguatera poisoning. Macroalgae samples were collected during research expeditions to Rarotonga (Cook Islands) and North Meyer Island (Kermadec Islands), from which two new *Gambierdiscus* species were characterized, *G. cheloniae* CAWD232 and *G. honu* CAWD242. Previous chemical and toxicological investigations of these species demonstrated that they did not produce the routinely monitored Pacific ciguatoxins nor maitotoxin-1 (MTX-1), yet were highly toxic to mice via intraperitoneal (i.p.) injection. Bioassay-guided fractionation of methanolic extracts, incorporating wet chemistry and chromatographic techniques, was used to isolate two new MTX analogs; MTX-6 from *G. cheloniae* CAWD232 and MTX-7 from *G. honu* CAWD242. Structural characterization of the new MTX analogs used a combination of analytical chemistry techniques, including LC–MS, LC–MS/MS, HR–MS, oxidative cleavage and reduction, and NMR spectroscopy. A substantial portion of the MTX-7 structure was elucidated, and (to a lesser extent) that of MTX-6. Key differences from MTX-1 included monosulfation, additional hydroxyl groups, an extra double bond, and in the case of MTX-7, an additional methyl group. To date, this is the most extensive structural characterization performed on an MTX analog since the complete structure of MTX-1 was published in 1993. MTX-7 was extremely toxic to mice via i.p. injection (LD_50_ of 0.235 µg/kg), although no toxicity was observed at the highest dose rate via oral administration (155.8 µg/kg). Future research is required to investigate the bioaccumulation and likely biotransformation of the MTX analogs in the marine food web.

## 1. Introduction

Ciguatera poisoning (CP) is the most prevalent, non-infectious, foodborne illness related to seafood consumption globally [1,2]. It has traditionally been associated with the bioaccumulation of marine toxins in fish flesh and viscera. However, over the last decade, additional CP vectors have been identified, including marine invertebrates, such as echinoderms (e.g., urchin, *Tripneustes gratilla*, and starfish, *Ophidiaster ophidianus*), gastropods (e.g., cone snails, *Conus* spp.) and bivalve molluscs (e.g., the giant clam, *Tridacna maxima*) [3,4,5,6,7], along with octopus (*Octopus cyanea*) and crustaceans (e.g., crab, *Percnon* spp., and lobster, *Panulirus penicillatus*) [8,9].

CP is prevalent in all the circumtropical regions of the world [10,11], and is particularly prolific throughout the tropical and sub-tropical waters of the South Pacific Ocean, affecting many indigenous island communities [2,12,13]. These communities are intrinsically linked to the reef system for subsistence and trade, which leaves them vulnerable to both the direct and indirect effects of CP [14].

The causative organism of CP in the Pacific Basin is the epiphytic, benthic, dinoflagellate genus *Gambierdiscus*. Global sea surface temperatures are increasing as a consequence of climate change, which is resulting in an expansion of the tropical and sub-tropical latitudes [15]. Therefore, the habitable range of *Gambierdiscus* is expanding further into sub-tropical and now temperate latitudes. Until the early 2000s, the habitable range of *Gambierdiscus* was thought to be limited to between the latitudes 35° N and 35° S (i.e., parts of the Pacific and Indian Oceans and the Caribbean Sea) [16]. However, recent studies have reported *Gambierdiscus* species in non-endemic areas, including Korea [17], Japan [18], the northern Gulf of Mexico [19], and the Mediterranean Sea [20].

The World Health Organization considers CP a neglected tropical disease worldwide, and in 2018, along with the Food and Agriculture Organization of the United Nations, held an expert meeting on CP. A report from the meeting summarized the collective multi-disciplinary knowledge of CP and highlighted several priority areas, with one being an improved understanding of the toxic secondary metabolites produced by *Gambierdiscus* species [21].

To date, *Gambierdiscus* has been demonstrated to produce a complex array of lipophilic and hydrophilic bioactive, cyclic polyether secondary metabolites, including Pacific ciguatoxins (P-CTXs) [22], maitotoxins (MTXs) [23,24], gambierones [25], gambierol [26], gambieroxide [27], and gambieric acids [28]. It is well documented that consumption of the aforementioned marine species contaminated with P-CTXs will lead to CP [29,30]. However, using liquid chromatography–mass spectrometry (LC–MS), only one species, *G. polynesiensis*, has been definitively shown to produce P-CTXs and the global distribution of this species does not align with the annual incidence rates of CP. Therefore, other secondary metabolites produced by *Gambierdiscus*, known and unknown, are likely to also play a role. In addition, *Gambierdiscus* cohabitates with other genera of toxin-producing benthic dinoflagellates, although it is unclear whether they also contribute to CP.

MTXs are a class of large cyclic polyethers that function as calcium channel antagonists. They are heavily hydroxylated hydrophilic compounds, which are either mono- or disulfated. MTX-1 is both the largest natural non-biopolymer and most toxic non-peptide compound known, with an LD_50_ of 50 ng/kg in mice via intraperitoneal (i.p.) injection [23]. MTX-1 is disulfated and comprised of 32 fused ether rings with aliphatic hydrocarbon chains at each terminus. Additional MTX analogs have also been described from *Gambierdiscus* spp., MTX-2 to MTX-5 [31,32,33], didehydro-demethyl-desulfo-MTX-1, and desulfo-MTX-1 [34]. However, based on the published mass spectral properties [31], MTX-3 was isolated, structurally elucidated, and determined to be 44-methylgambierone [35,36]. Elucidating the structure of MTX analogs is extremely complex and requires multiple analytical chemistry techniques. Of the MTX analogs reported to date, excluding MTX-3, the complete molecular structure has only been elucidated for MTX-1 and the determination spanned a 25-year body of research [23,37,38,39,40,41].

During research expeditions to Rarotonga (the Cook Islands) and North Meyer Island (the Kermadec Island group), various macroalgae samples were collected. Two new *Gambierdiscus* species were identified in these samples, morphologically characterized and published as *G. cheloniae* [42] and *G. honu* [43]. In 2017, following the identification of these species, a comparative study of 16 *Gambierdiscus* isolates was conducted [44], using LC–MS and the mouse bioassay (MBA). This demonstrated that both species were highly toxic to mice yet did not produce any of the routinely monitored P-CTXs nor MTX-1.

This manuscript describes the use of MBA-guided fractionation to isolate two new MTX analogs, MTX-6 and MTX-7, produced by *G. cheloniae* CAWD232 and *G. honu* CAWD242, respectively. The chemical structures of these new analogs were characterized using multiple analytical techniques, with concurrent MS experimental data generated for MTX-1 to aid in the characterization. The acute toxicity of the MTX analogs was determined in mice using i.p. injection and oral administration.

## 2. Results

### 2.1. Purification of Maitotoxins

Sequential monoclonal cultures of *G. cheloniae* CAWD232 and *G. honu* CAWD242 were grown (Section 4.1) and an MBA was used to guide the isolation of the toxic compounds produced by these isolates. Cultures were harvested in the stationary phase, extracted, and clarified using low-temperature protein precipitation and sequential membrane filtration. Extracellular phospholipids and highly lipophilic compounds were removed via a liquid–liquid partition (Section 4.2).

#### 2.1.1. Maitotoxin-6

A second liquid–liquid partition separated the hydrophilic MTX-like and lipophilic CTX-like compounds, with the MBA revealing the target compound (MTX-6) was hydrophilic in nature. Purification of MTX-6 was achieved using three liquid chromatography techniques: a solid-phase extraction (SPE) cartridge, flash chromatography, and preparative high-performance liquid chromatography (HPLC; Section 4.3). At each stage, fractions resulting from each separation technique were tested on mice and the toxicity was tracked. Two important observations were made using the HPLC system; basic mobile phases were essential for the elution of the target compounds from the preparative HPLC column (although this chromatographic behavior was not observed using the flash chromatography column) and MTX-6 had inconsistent chromatographic behavior between the analytical and preparative systems (which was unexplained at the time).

#### 2.1.2. Maitotoxin-7

As above, the MTX-like and CTX-like compounds were separated using a liquid–liquid partition, although, with MTX-7, pH control was critical for clean separation. The MBA confirmed the target compound (MTX-7) was also hydrophilic and it was subsequently purified using a SPE cartridge, flash chromatography, and preparative HPLC (Section 4.4). As with the isolation of MTX-6, MTX-7 had inconsistent chromatographic behavior between the analytical and preparative systems. Multiple parameters were investigated, which revealed that column temperature played the most critical role.

### 2.2. Liquid Chromatography–Mass Spectrometer Analysis of Intact Maitotoxins

LC–MS scanning experiments (*m*/*z* 48–2000; Section 4.5) of the three MTX analogs (MTX-1, MTX-6, and MTX-7) revealed signals pertaining to the doubly-, triply-, and quadruply-charged deprotonated molecular ions in −Electrospray Ionization (ESI) mode (Figure S1), and doubly- and triply-charged ions in +ESI mode (Figure S2). Dominant ions were fragmented using collision-induced dissociation (CID), with optimal collision energies (CEs) experimentally determined as 60 eV for −ESI mode and 40 eV for +ESI mode. Triply-charged ions fragmented more readily and provided more spectral information, with −ESI having the best sensitivity. The fragmentation spectra of MTX-6 and MTX-7 were similar in the *m*/*z* 110–950 region (−ESI mode), which was different from that of MTX-1 (Figures S3 and S4). All three analogs yielded the same *m*/*z* 97 fragment ion in −ESI, representing the bisulfate anion, which increased in intensity as the CE was raised.

### 2.3. High-Resolution Mass Spectrometer Analysis of Intact Maitotoxins

Similar scanning experiments were performed on the three MTX analogs using a HR–MS system (Section 4.6). The known chemical formula of MTX-1 (C_164_H_258_O_68_S_2_) was used to assign the +ESI and −ESI spectra for the singly, doubly, and triply charged ions (Figures S5–S9). The mass errors for the different ions ranged from +2.88 to −1.57 ppm, except for the singly charged anion, which was outside the calibration range of the instrument and had a mass error of −7.13 ppm. Masses pertaining to the [M+2H–2H_2_O]^2+^ (*m*/*z* 1673.8099), [M+2H–H_2_O–SO_3_]^2+^ (*m*/*z* 1642.8368), [M+2H–2H_2_O–SO_3_]^2+^ (*m*/*z* 1633.8314), followed by [M+2H–2SO_3_]^2+^ (*m*/*z* 1602.3569), and sequential [M+2H–*n*H_2_O–2SO_3_]^2+^ ions (*m*/*z* 1593.3508, 1584.3458 and 1575.3391) were observed in the +ESI spectrum of the doubly-charged cations (Figure S10). In the −ESI mode, there was a single peak representing either the [M–2H–SO_3_]^2−^, [M–2H]^2−^, or a formate adduct ion, in the singly-, doubly-, and triply-charged spectra, respectively (Figures S7–S9).

The isotope patterns of the dominant singly- and doubly-charged ions, in +ESI and −ESI, were mapped against the ‘theoretical isotope distribution’ calculated using the in-house developed National Research Council Canada (NRCC) ‘Molecular Formula Calculator v1.01’ [45]. The cations aligned well; for example, the [M+2H]^2+^ (Figure S11) and [M+H]^+^ ions, as well as the [M–2H]^2−^ ion. However, the [M–H]^−^ ion, which was outside the calibration range of the mass spectrometer, did not.

**Figure 1:**
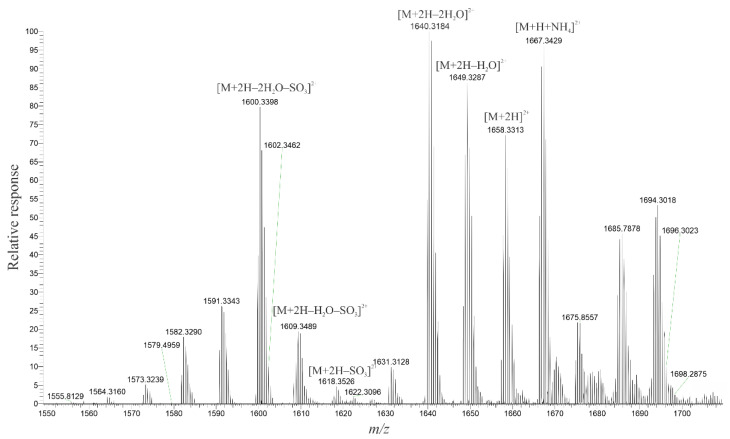
High-resolution mass spectrum of the doubly-charged cations of MTX-6, displaying the *m*/*z* range 1550–1710.

**Figure 2:**
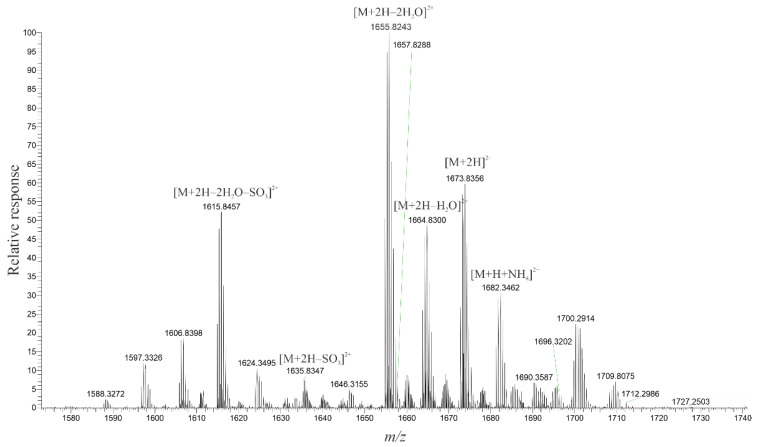
High-resolution mass spectrum of the doubly-charged cations of MTX-7, displaying the *m*/*z* range 1570–1740.

Interpretation of the spectra for MTX-6 and MTX-7 was more complicated as the chemical formulae were unknown. However, as with MTX-1, the +ESI spectrum provided more structural information, with the spectra of the doubly-charged ions being particularly informative for MTX-6 (Figure 1) and MTX-7 (Figure 2). Most notable in the spectra were ions resulting from the loss of water, followed by the loss of a single sulfate and sequential water loss ions, indicating the two new MTX analogs were monosulfated, in contrast to MTX-1, which is disulfated. This observation was supported by the spectra of the singly- and triply-charged ions (Figures S12–S15). In −ESI mode, the deprotonated molecular ion was observed in the singly-charged spectra (Figures S16 and S17), whereas, in contrast to MTX-1, a formate adduct dominated the signal in both the doubly- and triply-charged spectra (Figures S18–S21).

The same ‘theoretical isotope distribution’ modeling was applied to the dominant ions observed for MTX-6. This aligned for both the singly- (which was outside the calibration range of the instrument) and doubly-charged ions in the +ESI ([M+H–2H_2_O]^+^ (Figure S22) and [M+2H–2H_2_O]^2+^) and −ESI spectra ([M–H]^−^ and [M−H+CHO_2_]^2−^). There was a double molecular ion observed in both the +ESI ([2M+2H]^2+^) and −ESI ([2M–2H]^2−^) doubly-charged spectra for MTX-7 (Figure S23), which meant the isotope model could only be applied to a single spectrum, the formate adduct of the doubly-charged anion (Figure S24).

Based on the spectra generated for MTX-6, the molecular formula was calculated to be C_164_H_256_O_66_S, with errors of −0.268 ppm and +0.915 ppm for the dianion and trianion, respectively. For MTX-7, the molecular formula was calculated to be C_165_H_258_O_67_S, with errors of −0.880 ppm and +0.240 ppm for the dianion and trianion, respectively. These chemical formulae were programmed into the HR–MS software and checked against several in-source fragments (+ESI, dominant doubly and triply charged ions), which aligned with errors between −0.03 and +2.67 ppm for MTX-6, and −2.10 and +0.26 ppm for MTX-7.

Comparing the chemical formulae above with that published for MTX-1 [23], it was deduced that MTX-6 had one less sulfur, two fewer oxygens, and two fewer hydrogens (−66 Da; Table 1), whilst MTX-7 had one less sulfur, one less oxygen, and an additional carbon atom (−36 Da; Table 2). To account for the net atom change, several modifications would need to occur. These hypothesized variations are also included in Table 1 and Table 2.

### 2.4. Oxidative Cleavage

To assist with the structural characterization of the new MTX analogs, oxidative cleavage experiments using periodate were performed to cleave vicinal diols, affording smaller fragments of the parent molecule. This approach was originally used to help determine the molecular structure of MTX-1.

**Figure 3:**
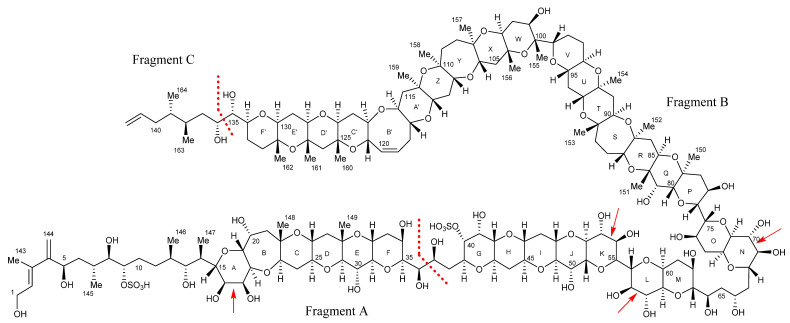
Oxidative cleavage schematic of MTX-1 depicting the six cleavage points between vicinal diols upon treatment with periodate. Red arrows indicate the vicinal diols in ether rings that will open, while the dotted red lines represent cleavage sites where the carbon backbone is broken, resulting in smaller sub-structures.

During the experiments with the new MTX analogs, MTX-1 was used as a model compound to evaluate how the technique performed and to the enable interpretation of the data. MTX-1 contains six vicinal diols, four in ether rings that are cleaved and open, and two in hydrocarbon linkages that fragment the backbone, creating three smaller sub-structures (Figure 3).

MTX-6 and MTX-7 were treated with periodate, and LC–MS scanning experiments (*m*/*z* 50–1800) were performed in −ESI mode on the reaction mixture. After a reaction time of 2 h, there were two unresolved peaks (1.31 and 1.35 min) that were identical in MTX-1 and MTX-7 (Figure S25A and S25B, respectively). The peak at 1.31 min had a dominant *m*/*z* 971.2 ion, representing Fragment A with dihydrated aldehydes in ring A ([M+2H_2_O–H]^−^; Figure S26A), and minor ions *m*/*z* 953.2 and *m*/*z* 935.3, representing lesser degrees of hydration ([M+H_2_O–H]^−^ and [M–H]^−^; Figure S26B and S26C, respectively). The peak at 1.35 min had a dominant *m*/*z* 985.3 ion, representing a hemiacetal ([M+H_2_O+CH_3_OH–H]^−^; Figure S26D), which resulted from the reaction occurring in aqueous MeOH.

Two unresolved peaks (1.36 and 1.39 min) were also observed in the MTX-6 trace with dominant ions of *m*/*z* 955.3 and *m*/*z* 969.3, which were hypothesized to also represent the dihydrated and hemiacetal variants of ‘Fragment A’, respectively (Figure S25C and S25D, respectively). It is currently unknown what structures the observed masses of MTX-6 represent. These experiments were monitored in −ESI mode, which confirmed the presence of the sulfate on C-9 and alluded to the likely scenario that MTX-6 and MTX-7 are desulfated at C-40.

The mass pertaining to the hemiacetal variant of MTX-1 and MTX-7 (*m*/*z* 985.3) was not observed when the oxidative cleavage products were analyzed using ammoniated mobile phases (Figure S27A), indicating it is labile under basic conditions. The corresponding mass observed in the MTX-6 trace (*m*/*z* 969.3) was also not present when analyzed using ammoniated conditions (Figure S27C), providing evidence that it also represented a hemiacetal variant.

**Figure 4:**
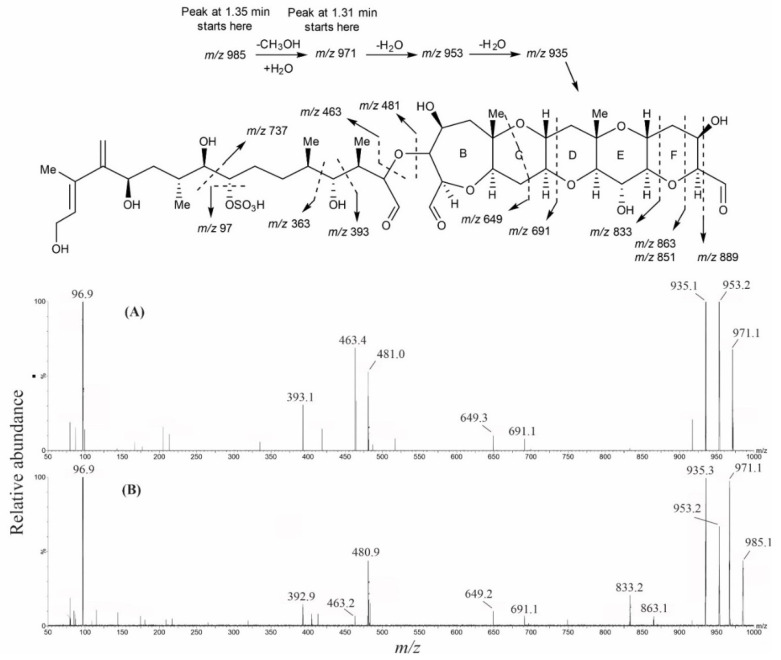
The proposed CID fragmentation of Fragment A in −ESI (60 eV CE; *m*/*z* 50–1000) and comparison of the corresponding spectra of (**A**) MTX-1 and (**B**) MTX-7.

CID fragmentation experiments were performed on the dominant oxidation products, *m*/*z* 971 and *m*/*z* 985 for MTX-1 and MTX-7, and *m*/*z* 955 and *m*/*z* 969 for MTX-6, to help identify structural similarities. A range of CEs was trialed with 60 eV being optimal and providing the most information (Figures S28–S30). Comparing the various CID spectra for both oxidation products, acquired using the different CEs, revealed an analogous fragmentation pattern for MTX-1 and MTX-7 (example spectra displaying some of the fragments in Figure 4). This demonstrated that Fragment A of MTX-1 and MTX-7 was the same.

Analysis of the equivalent ‘Fragment A’ for MTX-6 revealed a mass reduction of 16 Da compared to MTX-1 and MTX-7 yet had fragment ions 2 Da higher than the equivalent MTX-1 and MTX-7 fragments. A comparison of the CID experiments of the three MTX analogs demonstrated that the hydrocarbon chain from C-1–C-13 (*m*/*z* 393; Figure S31; displayed in red) is likely the same for MTX-1, MTX-6, and MTX-7, while the remaining structure of ‘Fragment A’ for MTX-6 is currently unknown.

HR–MS experiments were performed on periodate treated samples to further deduce structural similarities and differences between the three MTX analogs. Analogous to the data generated for the intact MTXs, +ESI mode provided the most structural information on the HR–MS instrument. After a 2 h reaction time, three main products were observed in the total ion chromatogram (TIC) of MTX-1, which represented Fragment A (4.55 min, *m*/*z* 823.4483, [M+H–2H_2_O–SO_3_]^+^), Fragment B (9.01 min, *m*/*z* 2316.0704, [M+H–H_2_O]^+^), and Fragments B and C connected (11.7 min, *m*/*z* 2360.2377, [M+H–SO_3_]^+^; Figure S32). The corresponding ions, in +ESI and −ESI modes, had mass errors ranging from +0.08 to +3.4 ppm. After a 24 h reaction time, the peak pertaining to Fragments B and C connected (11.7 min) was absent and the peak representing Fragment B only (9.01 min) had enlarged accordingly. The mass of Fragment C only was outside the scan range used for the experiments.

The MTX-6 TIC revealed three main products, which represented ‘Fragment A’ (4.77 min, *m*/*z* 823.4477) and two unknown ions *m*/*z* 1551.8261 and *m*/*z* 1665.9308 (10.48 and 12.95 min, respectively; Figure S33). The ions, when observed in the +ESI and −ESI modes, had mass errors ranging from +0.06 to +1.7 ppm. CID fragmentation of the *m*/*z* 1551.8261 and *m*/*z* 1665.9308 ions, using 40 eV of CE, showed similar mass fragments, indicating there was some commonality in the backbone of these two MTX-6 oxidation products (Figure S34). This led to the hypothesis that these two ions represent a variation of ‘Fragment B’ for MTX-6, with and without ‘Fragment C’ attached. The difference between the dominant higher mass fragments, *m*/*z* 1551.8253 and *m*/*z* 1647.9199 (Figure S34), was 96.0946 Da (C_7_H_12_). Fragment C from MTX-1 was *m*/*z* 124.1228 (C_9_H_16_), suggesting that ‘Fragment C’ from MTX-6 has C_2_H_4_ (28.0282 Da) less than MTX-1.

As per the other MTX analogs, there were three main products observed in the MTX-7 TIC. These represented Fragment A (4.55 min, *m*/*z* 823.4477) and two unknown ions, *m*/*z* 1567.8214 and *m*/*z* 1709.9563 (9.94 and 12.82 min, respectively; Figure S35). Mass errors for the corresponding ions, in +ESI and −ESI modes, ranged from −1.0 to +1.1 ppm. Similar mass fragments were observed during the CID experiments of the unknown ions, indicating the two MTX-7 products had the same backbone (Figure S36). As per MTX-6, it was hypothesized the ions represented Fragment B with and without Fragment C attached. The difference between the dominant higher mass fragments, *m*/*z* 1567.8208 and 1691.9461 (Figure S36), was 124.1253 Da (C_9_H_16_), confirming that Fragment C was the same for MTX-7 and MTX-1. Therefore, the structural differences between MTX-7 and MTX-1 are located in ‘Fragment B’.

**Figure 5:**
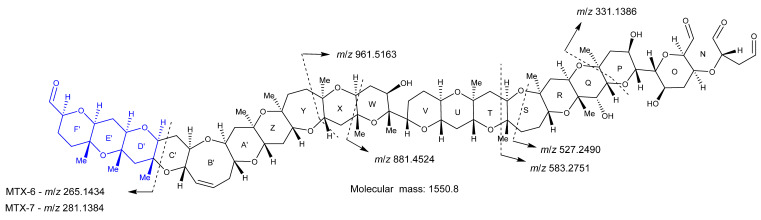
Proposed CID fragmentation pathway for the truncated ‘Fragment B’ of MTX-6 and MTX-7, based on the chemical structure of MTX-1, showing the common fragment ions produced under collision-induced dissociation. The highlighted region (in blue) is where the additional oxygen atom in MTX-7 is likely located.

Using the chemical structure of Fragment B from MTX-1 as a guide, the mass spectral data acquired for MTX-6 and MTX-7 were compared. This provided evidence that ‘Fragment B’ observed for MTX-6 and MTX-7 represented a truncated version of that in MTX-1. It was deduced that an additional hydroxyl group on C-65 of MTX-6 and MTX-7 created a vicinal diol, which cleaved when oxidized with periodate, producing the smaller sub-structure. Analysis of the oxidative cleavage products in the −ESI mode also confirmed that the C-40 sulfate group in MTX-1 was not present in MTX-6 and MTX-7, as this fragment was not observed. In addition, the difference of 44.0262 Da observed between the combined ‘Fragments B and C’ of MTX-6 (*m*/*z* 1647.9199) and MTX-7 (*m*/*z* 1691.9461) corresponded to C_2_H_4_O. As demonstrated above, ‘Fragment C’ of MTX-6 lacks C_2_H_4_ (compared to MTX-1 and MTX-7), meaning that the three fragments vary by only a single oxygen atom in rings N−Fʹ. To elucidate where the additional oxygen atom in MTX-7 is located, a comparison of the CID fragmentation data for the respective Fragment B’s (acquired using a range of CEs) from MTX-6 (*m*/*z* 1551.8261) and MTX-7 (*m*/*z* 1567.8214) was performed (example mass spectra in Figure S37). This revealed common ions, representing rings N–X (based on the structure of MTX-1; Figure 3) in both MTX analogs, with some lower mass fragments representing rings D′–F′ also observed. Subsequent experiments using a higher CE afforded a stronger signal for the lower mass fragments, with a *m*/*z* 265.1434 ion in MTX-6 and a *m*/*z* 281.1384 ion in MTX-7. The mass difference (15.995 Da) indicated that the additional oxygen atom in MTX-7 was located in rings Dʹ−Fʹ (blue portion Figure 5).

HR–MS CID fragmentation (using a range of CEs) of the *m*/*z* 823.448 ions (Fragment A, C_43_H_67_O_15_, mass errors of −1.0 to +1.3 ppm) observed at 4.55 min in MTX-1 and MTX-7, and 4.77 min in MTX-6 was performed (example spectra in Figure S38). The fragmentation patterns of MTX-1 and MTX-7 were the same, confirming the LC–MS observation that MTX-1 and MTX-7 have the same Fragment A. MTX-6 also showed identical lower mass fragments (*m*/*z* 95–221). However, the fragmentation ions in the *m*/*z* 245–500 region were 2 Da higher in mass than the equivalent ions observed in MTX-1 and MTX-7, which is analogous to the LC–MS experiments (Figure S31). Further investigation into the 2 Da mass difference, while using the HR–MS molecular formula calculator, suggested the difference between MTX-6 and MTX-1/MTX-7 is the addition of C_4_H_2_ and loss of three O atoms. Exactly how/where these structural modifications have occurred remains unclear at this stage.

### 2.5. Reduction of the Oxidative Cleavage Products

The reaction products from the oxidative cleavage experiments were subsequently reduced using sodium borohydride to afford single C–OH bonds and LC–MS scanning experiments (*m*/*z* 900−1100) were performed in −ESI mode. After a 2 h reaction time, there were two unresolved peaks (1.90 and 1.92 min) in the spectra for MTX-1 and MTX-7, which had dominant ions of *m*/*z* 941.3 and *m*/*z* 939.3, respectively (Figure S39). The difference of 6 and 4 Da compared to the non-reduced version of Fragment A (*m*/*z* 935.3; Figure S40A) represented Fragment A with either all three aldehydes reduced (Figure S40B) or only two (Figure S40C). This confirmed the presence of three aldehydes in Fragment A of MTX-1 and MTX-7. There was a single peak (1.96 min) in the trace for MTX-6 (*m*/*z* 939.3), which had a difference of 2 Da compared to the non-reduced product (*m*/*z* 937.3). This indicated that only a single aldehyde was present, which was hypothesized to be at the oxidative cleavage site, meaning the vicinal diols in ring A are not present in ‘Fragment A’ of MTX-6. The mass of this fragment was the same as that observed in MTX-1 and MTX-7; however, it eluted later, indicating a different structure or stereochemistry, with the exact nature of the difference unknown (Figure S39).

### 2.6. Nuclear Magnetic Resonance Spectroscopy of Maitotoxin-7

Using the structural features reported for MTX-1 [23,37,38,39,46] as a guide, an extensive series of 1D- and 2D-NMR experiments were performed on an 800 MHz spectrometer to structurally characterize MTX-7. These included ^1^H, homonuclear decoupled ^1^H, DEPT135Q, COSY, TOCSY, NOESY, ROESY, HSQC, HMBC, SHSQC, and SHMBC spectra, along with a series of higher-resolution (20, 40, 80 and 160 msec) 1D-SELTOCSY, SELROESY and SELNOESY spectra (Section 4.7). The 1D–SELECTIVE experiments were performed with carefully attenuated excitation pulse powers, such that methyl group signals that differed in their chemical shift by less than 0.01 ppm, from that of a neighboring methyl group, could be selectively excited in SELTOCSY, SELNOESY, and SELROESY experiments.

The ^1^H NMR spectrum of MTX-7, acquired with excitation sculpturing (ES) and/or continuous wave (CW) presaturation of the HOD and/or MeOH solvent signals, was extremely complex (Figure S41). The upfield and downfield regions, representing the methyl groups (Figure S42A) and olefin protons (Figure S42B) respectively, provided key structural insights. However, there was a significant overlap of the signals pertaining to the cyclic polyether backbone (3.5–4.5 ppm region; Figure S43). The signal-to-noise ratio of the DEPT135Q spectrum (which identified quaternary carbon signals as well as methyl, methylene, and methine carbon signals) was sufficient to identify most, but not all, the ^13^C signals of MTX-7. Correlations observed in the HSQC, SHSQC, HMBC, and SHMBC spectra also contributed to the identification of some of the ^13^C chemical shifts of MTX-7. As with the proton spectrum, there were multiple overlapping ^13^C signals, particularly those pertaining to the carbon atoms of the cyclic polyether backbone (65–90 ppm; Figure S44). To enable the ^1^H and ^13^C NMR methyl group chemical shifts to be differentiated, they were reported to three and two decimal places, respectively (where applicable; Table S1).

There were 22 methyl groups present in the proton spectrum of MTX-7, representing five secondary and 17 tertiary methyl groups, including a downfield olefinic methyl signal at 1.82 ppm (Table S1). The methyl groups are numbered according to their chemical shift, starting upfield. Compared to MTX-1, MTX-7 contained one additional methyl group, which was consistent with the MS data generated for this analog. Expansion of the 8–42 ppm region of the DEPT135Q spectrum (Figure S45) enabled all the methyl group ^13^C signals to be identified (Table S1), with the correlations to specific proton signals (Figure S43A) identified using the HSQC and higher-resolution SHSQC spectra (Figure S46).

Two methylene protons were observed at 2.78 and 2.27 ppm in the SHSQC spectrum (Figure S47), each of which exhibited a TOCSY correlation to an olefinic proton signal at 5.73 ppm. This was analogous to the TOCSY correlations observed for the H-118_a,b_ protons of MTX-1 [23] and consistent with MTX-7 having the same ring B′ (*Z*)-double bond. The proton signal at 2.78 ppm was particularly diagnostic of an allylic methylene proton. Substantial overlap of the remaining methylene proton signals was observed in the 1.4–3.0 ppm region and due to the complexity of the spectra, a complete assignment of the signals was not possible with the current data set.

The five methine proton signals coupled to the secondary methyl groups were identified using the COSY spectrum (Figure S48), while the chemical shifts of the methine carbon atoms were determined using the SHSQC spectrum (Table S2). The significant overlap of the methine proton signals arising from either the oxygenated ring junction or hydroxylated (-C*H*-O-C*H* and -C*H*OH) groups, occurred in the 2.9–4.6 ppm region of the ^1^H and SHSQC (Figure S49) spectra. Due to the complexity of these spectra, a complete assignment of the methine group ^1^H and ^13^C signals could not be performed.

The ^1^H, COSY (Figure S50), and HSQC (Figure S51) NMR spectra showed correlations pertaining to five individual protons or group olefinic signals (4.9−5.9 ppm). Four of these were analogous to those published for MTX-1 [23]; the single H-2 olefinic proton (5.68 ppm), the pair of 4=CH_2_ olefinic protons (H-144_a,b_, 5.12, 5.26 ppm), a pair of (*Z*)- coupled olefinic ring B′ protons (H-119 and H-120, 5.73 and 5.63 ppm, respectively), and the terminal H-141 (5.81 ppm) and H-142_a.b_ olefinic protons (4.96, 4.99 ppm). The fifth group of olefinic signals observed in MTX-7 (5.48 and 5.68 ppm) were different from those of MTX-1 and consisted of a pair of (*Z*)- coupled olefinic protons (labeled as the 2^nd^ double bond protons in Figures S50 and S51).

**Figure 6:**
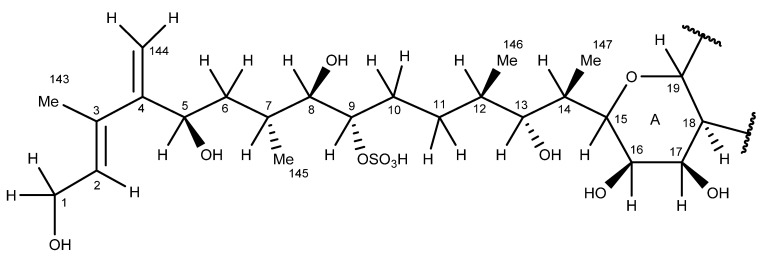
Structure of the aliphatic side chain C-1–C-14 connected to ring A in MTX-7 and numbered according to the data published for MTX-1 by Murata et al. [23].

The aliphatic side-chain atoms C-1–C-14 (numbered according to the structure of MTX-1) [23] connected to ring A (Figure 6) were elucidated using a combination of the ^1^H, COSY, TOCSY, NOESY, ROESY, HSQC, and HMBC experiments (Figures S52–S58), supplemented by higher-resolution 20, 40, 80, and 160 msec 1D-SELTOCSY, SHSQC, SHMBC, and DEPT135Q experiments.

The series of 20–160 msec 1D-SELTOCSY spectra (the 160 msec spectra are shown in Figure S59) acquired for the three-ring A aliphatic side-chain secondary methyl groups, which occurred at 1.03 ppm (7-CH_3_), 0.98 ppm (12-CH_3_), and 0.94 ppm (14-CH_3_), enabled the chemical shifts of the protons in close proximity to these methyl groups to be progressively identified. However, the SELTOCSY spectra acquired for the 0.94 ppm 14-CH_3_ protons did not identify the resonance of H-15. The resonance of this proton was tentatively identified via an HMBC correlation observed between the 14-CH_3_ group and the ^13^C signal at 74.9 ppm, which showed an HSQC correlation to the proton signal at 3.97 ppm.

Long-range SELTOCSY correlations enabled the H-5 (4.50 ppm) and H-9 (4.41 ppm) signals to be differentiated. This was based on both protons exhibiting correlations to the 7-CH_3_ group (1.03 ppm), with H-9 (4.41 ppm) also showing a correlation to the 12-CH_3_ (0.98 ppm) group. The chemical shift of the H-9 methine proton (4.41 ppm) was consistent with a sulfate group attached at C-9; therefore, confirming that MTX-7 is desulfated at C-40 as indicated by the MS data. The significantly greater chemical shift of the 5-CHOH methine proton (4.50 ppm), compared to that of the 13-CHOH methine proton (3.35 ppm), was attributed to the proximity of the allylic methine proton (H-5) to the 4=CH_2_ group.

A correlation observed in the ROESY spectrum of MTX-7 was consistent with a (*Z*)- relationship between the 1-CH_2_OH group (4.20 ppm) and the olefinic 3-CH_3_ group (1.82 ppm). In addition, a correlation between the 3-CH_3_ group (1.82 ppm) and the H-144_a_ proton (5.11 ppm, i.e., one of the 4=CH_2_ protons) was observed. Both the 12-CH_3_ (0.98 ppm) and 14-CH_3_ (0.94 ppm) groups showed an HMBC correlation to the 13-CHOH group (79.5 ppm), which enabled it to be differentiated from the 5-CHOH and 8-CHOH signals. HMBC correlations were also exhibited by the protons of the 12-CH_3_ and 14-CH_3_ groups and the reciprocal C-12 and C-14 methine carbon signals (37.2 and 35.8 ppm, respectively).

The NMR experiments described above confirmed the C-1–C-14 side-chain atoms of MTX-7 (Figure 6; Table S3) to be the same as those of MTX-1. In the absence of specific stereochemical information, asymmetric side-chain atoms have been assumed to possess the same stereochemistries as those published for the corresponding atoms of MTX-1 [23].

**Figure 7:**
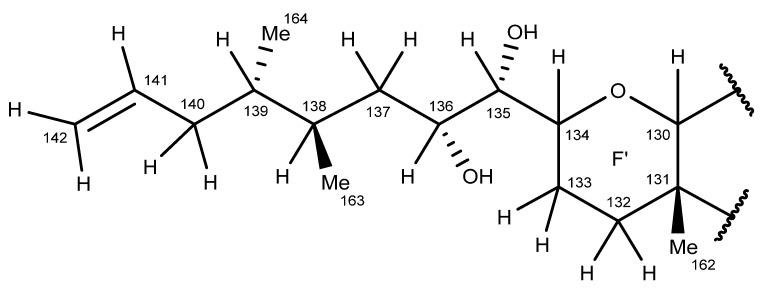
Structure of the aliphatic side chain C135–C142 connected to the ring F′ observed in MTX-7 and numbered according to the data published for MTX-1 by Murata et al. [23].

The same NMR experiments described above were used to elucidate the proton signals of the aliphatic side-chain atoms C-135−C-142 connected to ring F′ (Figure 7). However, due to the severe overlap observed in the HSQC and SHSQC spectra, not all the carbon atom chemical shifts could be determined.

**Figure 8:**
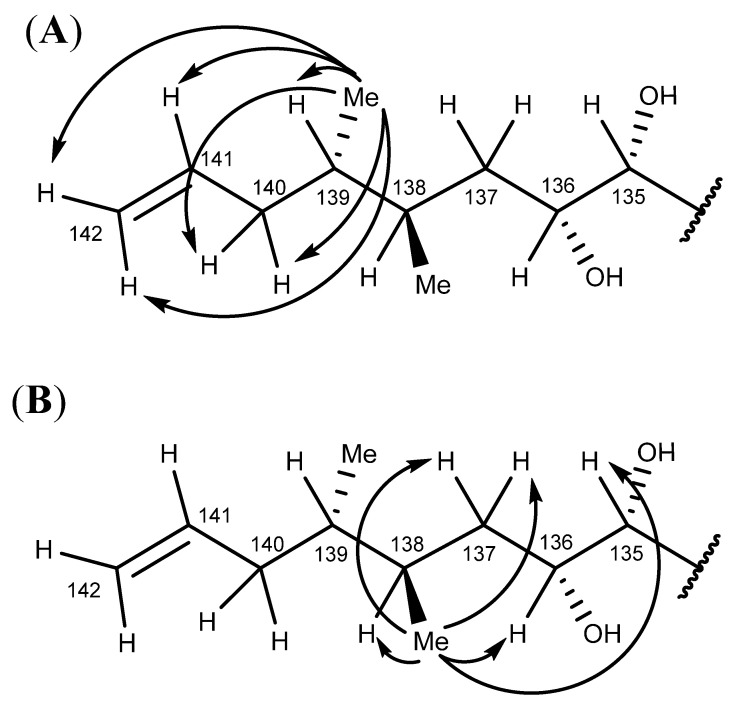
The 1D-SELTOCSY correlations observed for (**A**) the 139-CH_3_ group (0.86 ppm) and (**B**) the 138-CH3 group (0.90 ppm) of MTX-7.

The SELTOCSY spectra (160 msec; Figure S60) determined for the two secondary methyl groups on the ring F′ side chain (138-CH_3_, 0.90 ppm; 139-CH_3_, 0.86 ppm) were used to trace out correlated spin systems and identify the signals of adjacent protons. The 139-CH_3_ group (0.86 ppm) exhibited TOCSY correlations to the H-139 (1.48 ppm), H-140_a,b_ (1.85, 2.15 ppm), H-142_a,b_ (4.96, 4.99 ppm), and H-141 (5.81 ppm) signals (Figure S60B and Figure 8A), while the 138-CH_3_ group (0.90 ppm) exhibited TOCSY correlations to the H-137_a,b_ (1.20, 1.56 ppm), H-138 (1.76 ppm), H-135 (3.13 ppm), and H-136 (3.70 ppm) signals (Figure S60C and Figure 8B).

The chemical shifts observed for the H-140_a,b_ methylene protons (1.85, 2.15 ppm) were greater than those of the H-137_a,b_ methylene protons (1.20, 1.56 ppm), which was consistent with the H-140_a,b_ protons being adjacent to the C-141−C-142 double bond. The TOCSY correlations exhibited by the H-142_a,b_ (4.96, 4.99 ppm) and H-141 (5.81 ppm) olefinic protons enabled the 139-CH_3_ (0.86 ppm) and the 138-CH_3_ (0.90 ppm) groups to be differentiated.

A moderate intensity triplet signal believed to arise from fatty acids and/or triglyceride impurities present in the MTX-7 sample, co-occurred with the 138-CH_3_ group (0.90 ppm). TOCSY correlations observed for the H-135 (3.13 ppm) methine proton, as depicted in a 1D-slice for this proton extracted from the 160 msec 2D-TOCSY spectrum (Figure S60A), enabled identification of the H-135 proton resonance via correlations exhibited to the H-133, H-134, H-136, and H-137 protons.

Both the 138-CH_3_ and 139-CH_3_ groups displayed HMBC correlations to methine carbons, which resonated at 34.3 and 39.8 ppm, along with correlations to different methylene carbon signals at 37.6 and 39.1 ppm, respectively. The 138-CH_3_ showed a correlation to C-139, while the 139-CH_3_ group showed a correlation to C-138, thereby confirming these groups are attached to adjacent side-chain atoms, and that neither of these secondary methyl groups was adjacent to the 135-CHOH or 136-CHOH groups. The methine proton signals of these groups occurred at 3.13 ppm (H-135) and 3.70 ppm (H-136), respectively. TOCSY correlations observed for the H-135 (3.13 ppm) methine proton confirmed the 136-CHOH proton and the H-137_a,b_ (1.20, 1.56 ppm) methylene proton assignments. H-135 (3.13 ppm) TOCSY correlations were also observed with the ring junction H-134 (3.93 ppm) methine proton, and the ring F′ H-133_a,b_ (1.81, 2.22 ppm) methylene protons. HSQC and higher-resolution SHSQC spectra confirmed the H-133 protons were attached to a methylene carbon, which resonated at 32.9 ppm.

**Figure 9:**
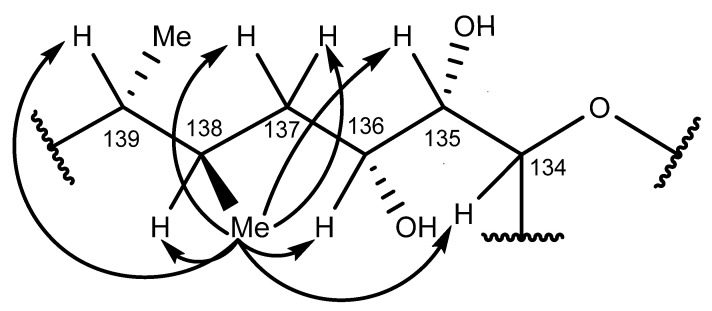
The NOESY correlations exhibited by the 138-CH_3_ (0.90 ppm) methyl group protons of MTX-7 showing correlations to H-134 (3.92 ppm), H-135 (3.31 ppm), H-136 (3.70 ppm), H-137_a,b_ (1.20, 1.56 ppm), H-138 (1.76 ppm), and H-139 (1.48 ppm) protons.

Strong correlations observed between the 138-CH_3_ (0.90 ppm) methyl group protons and the H-136 (3.70 ppm) and H-138 (1.76 ppm) protons in the NOESY spectrum (Figure S63) were used to confirm the signal assignment. Less intense NOESY correlations were also observed with the more distant H-134 (3.92 ppm), H-135 (3.31 ppm), H-137_a,b_ (1.20, 1.56 ppm), and H-139 (1.48 ppm) protons (Figure 9).

The NMR experiments described above confirmed that the C-135−C-142 side-chain atoms of MTX-7 (numbered according to the structure of MTX-1 [23]; Figure 7; Table S4) are the same as those of MTX-1. As above, in the absence of specific stereochemical information, asymmetric side-chain atoms have been assumed to possess the same stereochemistries as those published for the corresponding atoms of MTX-1 [23];

HR–MS CID experiments indicated there was an additional oxygen atom attached to either ring D′, E′, or F′ (Figure 6) in MTX-7. Analyses of NOESY, ROESY, HSQC, and HMBC data, together with considerations of the NMR data published for MTX-1 [23], were used to locate the additional oxygen atom. Moreover, 19 of the 22 methyl groups identified in MTX-7 (numbered according to their chemical shift, starting upfield) displayed HMBC correlations to two oxygenated carbons and a methylene carbon (Table S5), as reported for MTX-1 [23]. The remaining three methyl group protons (1.202, 1.23, and 1.36 ppm) displayed HMBC and SHMBC correlations to three oxygenated carbons (Table S5). The C-151-CH_3_ and C-155-CH_3_ groups in MTX-1 were situated adjacent to secondary hydroxy groups at C-30 and C-101, respectively, and a pair of ring junction secondary ether or quaternary oxygenated carbons. As demonstrated using HR–MS, the backbone rings N−C′ of MTX-7 were the same as those of MTX-1 [23]. This accounts for two of the three methyl group signals in MTX-7 displaying correlations to three oxygenated carbons. The third methyl group was, therefore, adjacent to an additional oxygenated group (hydroxyl) that was not present in MTX-1 [23].

The methyl group signal that occurred at 1.44 ppm showed NOESY correlations to methyl group signals at 1.36 and 1.32 ppm (Figure S62). Assuming the ring B’–F’ region of MTX-7 is the same as in MTX-1, this chain of three mutual NOESY correlations can be attributed to the methyl groups attached to C-125, C-127, and C-131. The NOESY and ROESY correlations were confirmed using a series of higher-resolution 1D-SELNOESY and SELROESY experiments.

The methyl signals that occurred at 1.32 ppm and 1.44 ppm exhibited HMBC correlations to the same methylene carbon signal (53.3 ppm), which was assigned to C-126. The 1.44 ppm methyl signal also displayed NOESY, ROESY, SELNOESY, and SELROESY correlations to the 1.36 ppm methyl group signal. Therefore, it follows that the 1.32, 1.44, and 1.36 ppm methyl groups are attached to C-125, C-127, and C-131, respectively. These methyl groups, (C-160, C-161, and C-162, respectively), are attached to rings C′–F′ of MTX-7 (assuming the structure in this region corresponds to that of MTX-1).

A unique feature of the HMBC correlations observed for the 1.36 ppm methyl group attached to C-131 was that it displayed correlations to three oxygenated carbons (Table S5), whereas the corresponding methyl group in MTX-1 only displayed correlations to two oxygenated carbons. Therefore, it was determined that an additional oxygen atom was attached to C-132 in MTX-7 (assuming the ring B’−F’ region is the same as MTX-1), with MS data confirming it was part of a hydroxyl group.

While the HSQC and HMBC data were used to determine the proton and carbon chemical shifts (3.72 and 77.3 ppm, respectively) of the C-132 hydroxyl group, the specific stereochemistry (equatorially or axially oriented) of this hydroxyl group could not be unequivocally determined due to overlap of six oxygenated proton signals in the 3.70−3.76 ppm region of the proton spectrum. However, it can be speculated that the C-132 hydroxyl group is likely to be equatorially oriented since this orientation would be less sterically hindered.

As described above and hypothesized based on HR–MS data (Table 2), an additional double bond and methyl group are present in MTX-7 compared to MTX-1. These functionalities account for the net atom change of hydrogen atoms. To date, detailed analysis of the COSY, TOCSY, NOESY, ROESY, HMBC, HSQC, and higher-resolution 1D-SELNOESY spectra, acquired using an array of experimental parameters, has not revealed the locations of the additional double bond and methyl group in MTX-7. The NOESY and HMBC spectra, however, provided two clues as to the location of the double bond. Firstly, the methyl signal that occurred at 1.274 ppm showed NOESY correlations to one of the pairs of methyl group signals that occurred at 1.35 ppm (Figure S62), and secondly, it also showed an HMBC correlation to an olefinic carbon signal at 142.2 ppm. While HR–MS evidence suggests the additional methyl group is located in the ring G−K region of MTX-7, the specific location was not able to be determined using the current data set. Therefore, it is not known if the 142.2 ppm HMBC correlation observed for the 1.274 ppm methyl group originates from an existing MTX-1-like methyl group or the new (additional) methyl group. There was no MS evidence of the additional double bond using the current data set.

### 2.7. Maitotoxin-7 Proposed Structure

**Figure 10:**
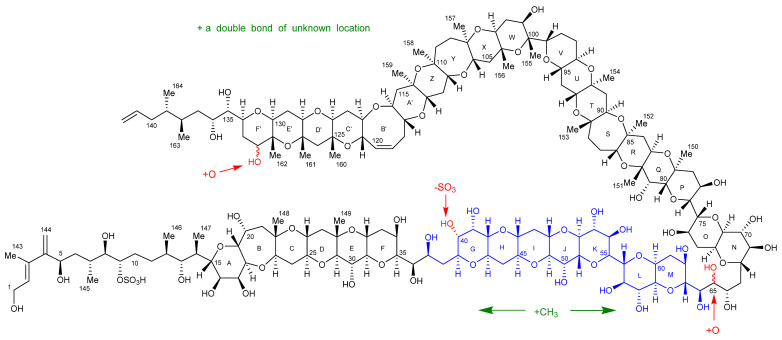
Structural hypothesis of MTX-7, displaying the similarities to MTX-1 (black), the sulfate–hydroxyl exchange at C-40 (red), the two additional hydroxyl groups at C-65 and C-132 (red), and where there is an additional methyl group (blue). The locations of the additional methyl group and double bond have not been determined.

Extensive combinations of 1D- and 2D-NMR experiments, LC–MS, HR–MS, oxidative cleavage, reduction, and CID experiments, with reference to the structure of MTX-1 [23], were performed to structurally characterize MTX-7. The characterization determined that the structure of the aliphatic side chain C-1–C-14, rings A–F, the aliphatic side chain C-135–C-142, the hydrocarbon linkage C-65–C-67, and rings N–F′ in MTX-7 are the same as in MTX-1. The sulfate group in MTX-1 on C-40 has been exchanged for a hydroxyl group; there are additional hydroxyl groups on C-65 and C-132 (via the addition of an oxygen atom); an additional methyl group between rings G–M; and an additional double bond of unknown location. These modifications account for the net atom change between MTX-1 and MTX-7 and led to the structural hypothesis presented in Figure 10.

### 2.8. Nuclear Magnetic Resonance of Maitotoxin-6

The ^1^H, COSY, TOCSY, and HSQC NMR spectra (Figures S63–S66) of MTX-6 contained high levels of both unknown and recognizable impurities, which, coupled with the extremely complex NMR spectra for the compound itself, meant that NMR data of sufficient quality could not be obtained, and only minimal structural insights were gained. NMR spectra of MTX-6 were recorded with combined ES and/or CW presaturation of the HOD peak at 4.8 ppm, which substantially attenuated the H-142_a,b_, and one of the H-144 proton signals. A comparison of the 5–6 ppm regions of the ^1^H and HSQC spectra of MTX-6 (Figures S67 and S68) and MTX-7 (Figure S51) showed the same five individual protons or groups of olefinic signals (H-2, 4=CH_2_ (H-144_a,b_), H-119, and H-120 (ring B’), H-141, and H-142_a,b_). This included those pertaining to the new double bond, whereas the methyl group signals (0.8–1.5 ppm), along with the methylene and methine signals (1.5–4.9 ppm), were indistinguishable via either direct or indirect COSY or TOCSY correlations.

### 2.9. Maitotoxin-6 Proposed Structure Nuclear Magnetic Resonance of Maitotoxin-6

**Figure 11:**
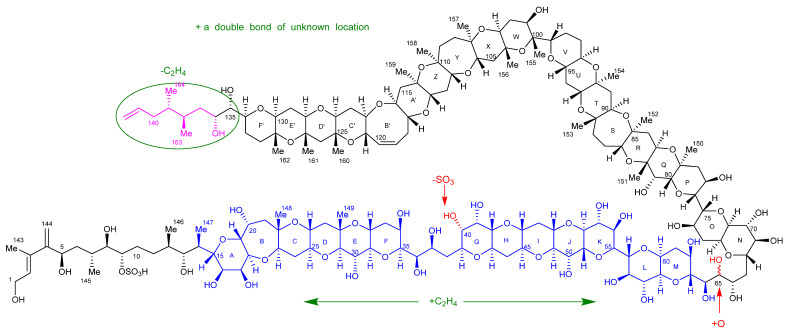
Structural hypothesis of MTX-6, displaying the similarities to MTX-1 (black), where there is an additional C_2_H_4_ (blue), the sulfate–hydroxyl exchange (red), the additional hydroxyl on C-65 (red), and where there is C_2_H_4_ less (pink). The exact locations of the C_2_H_4_ modifications have not been determined. Note: the stereochemistry of rings A–F is most likely different from that of MTX-1 and MTX-7, as demonstrated by having a different retention time and CID fragmentation pattern.

While the characterization of MTX-6 was less complete than for MTX-7, some structural insights were gained from the LC–MS, HR–MS, oxidative cleavage, and CID experiments. These included that the aliphatic chain C-1–C-13, hydrocarbon linkage C-65–C67, and rings N–F′ of MTX-6 were the same as in MTX-1; the sulfate group in MTX-1 on C-40 was exchanged for a hydroxyl group; there is an additional hydroxyl group on C-65; there is an additional C_2_H_4_ between rings A–M; ‘Fragment C’ is C_2_H_4_ less than MTX-1, and there is an additional double bond of unknown location. These modifications account for the net atom change between MTX-1 and MTX-6 and led to the structural hypothesis presented in Figure 11.

### 2.10. Acute Toxicity

Both LC–MS/MS and NMR spectroscopy indicated that MTX-7 was of high purity and a nominal concentration of 0.47 mg/mL was determined by gravimetric analysis. Using this material, the acute toxicity of MTX-7 (Section 4.8) was determined in mice via i.p. injection using the up-and-down procedure as described in the Organisation for Economic Cooperation and Development (OECD) guideline 425 [47]. Where applicable, mice were euthanized to prevent long-term suffering in accordance with the requirements of the OECD Humane Endpoints Guidance Document [48].

The LD_50_ of MTX-7 by i.p. injection was determined to be 0.235 µg/kg, (95% confidence intervals of 0.208 µg/kg and 0.326 µg/kg). At necropsy, the intestinal cavity of mice administered with a lethal dose contained a bloody fluid and the intestinal tract was distended and filled with a pale green fluid. Oral administration was achieved by dosing a mixture of ground mouse food and MTX-7 material ‘over the tongue’ of the mouse (Section 4.8.3). No oral toxicity was observed at the highest dose rate administered (156 µg/kg).

The high gravimetric mass of MTX-6 (2.66 mg) and the impurities observed in the MS and NMR spectra, indicated that MTX-6 was not of high purity. Therefore, the LD_50_ measurement of 6.45 µg/kg (95% confidence intervals of 5.93 and 6.65 µg/kg) by i.p. injection is indicative only. At necropsy, the gut of the mouse administered with a lethal dose was blackened and the stomach contained very little food, along with black particles. The intestinal tract contained a black runny paste and the caecum was also filled with solid black/green material. No oral toxicity was observed at the highest dose rate administered (891 µg/kg).

## 3. Discussion

Identifying the toxic secondary metabolites produced by *G. cheloniae* CAWD232 and *G. honu* CAWD242 is essential in ascertaining if these species play a role in CP events. Using the MBA as a guide, methanolic extracts were fractionated using liquid–liquid partitioning, benchtop SPE, and flash and preparative HPLC. Learnings from the isolation of MTX-6 from *G. cheloniae* CAWD232 meant that during the liquid–liquid partition and SPE fractionation of *G. honu* CAWD242, acidic conditions were used to isolate MTX-7.

A critical observation was made during the preparative HPLC fractionation, basic conditions were essential for the elution of sulfated cyclic polyethers (SCPs) from the stationary phase. This represents a potentially elegant way to purify SCPs, namely, loading under acidic conditions when the sulfate is protonated and has a strong binding affinity to the stationary phase, then elute under basic conditions when the sulfate is deprotonated and has less binding affinity. This, however, needs to be explored further.

An initially unexplained observation was also made during the preparative HPLC fractionation; the target compounds switched elution order between the analytical and preparative systems. An investigation into why this occurred included examination/consideration of the injection solvent, column temperature, loading capacity, void volume, stationary phase pore size, and the potential of physicochemical properties that could modify the affinity of the target compounds with the stationary phase. It was deduced that, of the parameters investigated, temperature played the most critical role in the binding affinity of large SCPs, such as MTXs.

The structural characterizations of MTX-6 and MTX-7, produced by *G. cheloniae* CAWD232 and *G. honu* CAWD242, respectively, were performed using a combination of analytical techniques, with comparison to data either experimentally determined or published for MTX-1 [23,37,38,39,40,41,46]. This involved LC–MS, LC–MS/MS, HR–MS, oxidative cleavage, subsequent reduction experiments, and NMR spectroscopy. The two new MTX analogs were initially identified using LC–MS, with oxidative cleavage and CID experiments performed to confirm that MTX-1, MTX-6, and MTX-7 had the same aliphatic hydrocarbon chain at one terminus (C-1–C-13), whilst MTX-1 and MTX-7 had the same cyclic polyether ring A–F system. Subsequent reduction experiments of the oxidation products confirmed the presence of three aldehydes in MTX-1 and MTX-7, whereas the mass difference of 2 Da observed with MTX-6 demonstrated that only one aldehyde was present. As an aldehyde would be formed via cleavage of the vicinal diols at C-36 and C-37 to generate the sub-structure being analyzed, it was determined that the ring A vicinal diols were not present in MTX-6.

HR–MS experiments enabled the chemical formulae to be determined for MTX-6 and MTX-7, which demonstrated net atom differences in the number of oxygen, hydrogen, and sulfur atoms. MTX-7 also contained one additional carbon atom, hypothesized to be present in the form of a methyl group. This was subsequently confirmed using NMR spectroscopy, where 22 methyl groups were identified, compared to 21 in MTX-1. The additional oxygen atom in MTX-6 and MTX-7 was located using HR–MS analysis of the oxidative cleavage products, which showed it was attached to C-65 in the form of a hydroxyl group. In turn, this created vicinal diols in the hydrocarbon linkage between C-64, C-65 and C-66, which were cleaved when treated with periodate. These experiments also demonstrated that MTX-6 and MTX-7 were desulfated at C-40, where the sulfate had been exchanged for a hydroxyl group. In addition, it was deduced that the aliphatic hydrocarbon chain at the other terminus (C-135–C-142) was the same for MTX-1 and MTX-7, whereas this portion of MTX-6 was C_2_H_4_ less. This also provided evidence that MTX-6 had an additional C_2_H_4_ between rings A–M compared to MTX-1 and MTX-7.

The location of the second additional oxygen atom in MTX-7 was narrowed down to rings D′–F′ using HR–MS analysis. NMR spectroscopy experiments were then used to identify the exact location, showing that it was attached to C-132 in the form of a hydroxyl group.

An extensive array of 1D- and 2D-NMR spectroscopy experiments on MTX-7 confirmed the MS results that the two aliphatic hydrocarbon chains at each terminus were the same as those in MTX-1. The experiments also demonstrated the presence of the sulfate group attached to C-9, thereby confirming that MTX-7 was desulfated at C-40. The additional double bond in MTX-6 and MTX-7 that was based on the net atom difference and hypothesized structural changes deduced via the HR–MS analysis, was also identified using NMR spectroscopy. However, despite the exhaustive experiments performed, the exact location of the additional double bond, and the exact location of the additional methyl group observed in MTX-7, could not be identified.

By i.p. injection, MTX-1 is the most toxic non-peptide toxin known, with an LD_50_ in mice of 0.05 µg/kg. However, the oral administration of MTX-1 producing cultures to mice showed considerably lower toxicity (there is no oral toxicity data available on purified MTX-1) [44]. To ascertain whether the newly described MTXs could contribute to CP, it was essential to evaluate their toxicity. By i.p. injection, the LD_50_s in mice were 6.35 and 0.235 µg/kg for MTX-6 and MTX-7, respectively, both of which were higher than that reported for MTX-1 (0.05 µg/kg). However, these values have been calculated from a nominal concentration based on gravimetric measurement and will likely be lower (meaning more toxic). To determine an accurate concentration, qNMR is required, and this will be investigated in the future. This is particularly relevant for MTX-6 as the LC−MS and NMR analysis showed this compound to be of low purity. The symptoms observed by the i.p. injection were similar for MTX-6 and MTX-7, although the onset of symptoms was much faster for MTX-7. The necropsy of animals given a lethal dose of either MTX analog showed a dramatic effect on the intestinal tract, including distention and the presence of extracellular fluid. Similar effects have been observed in mice injected with the crude *Gambierdiscus* extracts [49]. Due to the limited information on the mouse toxicity of MTX-1, it is unknown whether the symptomology is comparable between the three analogs.

Oral administration was performed using the ‘over-the-tongue’ method rather than by gavage, which is often the method chosen for oral dosing of mice. This is due to gavage having been shown to overestimate the toxicity of secondary metabolites. This is thought to be due to the consistency of the stomach contents of rodents, which, unlike a human, is semi-solid. This means the liquid dose administered by gavage can flow around the semi-solid mass to be rapidly absorbed by the duodenum. Using the ‘over-the-tongue’ method, MTX-6 and MTX-7 showed no toxicity at the highest dose rates administered (891 and 156 µg/kg, respectively). Due to the limited quantity of the new MTX analogs, it was not possible to administer higher dose rates. The absence of oral potency for MTX-6 and MTX-7 indicates they are unlikely to contribute to human intoxications. However, further investigation is required to determine if MTXs are biotransformed into more toxic analogs as they are bioaccumulated in the marine food web, as is observed with the P-CTXs.

## 4. Materials and Methods

### 4.1. Culturing

The two isolates, *G. cheloniae* CAWD232 and *G. honu* CAWD242, which are maintained in the Cawthron Institute Culture Collection of Microalgae, were grown in f/2 seawater (1:3; UV treated and filtered down to 0.22 µm). The culturing cabinet was set at 25 °C (±2 °C) with 40–70 µmol m^−2^ s^−1^ photon irradiance (12:12 h light:dark cycle). Consecutive 5 L monoclonal cultures (total of 100 L per isolate) were grown to produce enough biomass (1.6 × 10^8^ cells for *G. cheloniae* (CAWD232) and 1.8 × 10^8^ cells for *G. honu* (CAWD242)) and harvested during the stationary phase of the growth cycle by centrifugation (3200× *g*, 10 °C, 10 min). The resulting cell pellets were frozen (–20 °C) until ready for extraction.

### 4.2. Extraction

The pelletized cells (Section 4.1) underwent an exhaustive triple extraction (sonication aided; 10 min) with 90% aq. MeOH (1 mL per 2 × 10^5^ cells). Cellular debris was pelleted by centrifugation (3200× *g*, 4 °C, 5 min) between extractions and the supernatant combined in a Schott bottle. The 90% aq. MeOH layer was collected and frozen (−20 °C) to precipitate extracellular co-extractives, followed by centrifugation (3200× *g*, 4 °C, 10 min) and sequential membrane and glass fiber filtration (8, 2, and 1.6 µm) to remove any fine particulates. To remove lipids, the 90% aq. MeOH extract was subjected to a liquid−liquid partition with *n*-hexane (1:1, *v*/*v*).

### 4.3. Isolation of Maitotoxin-6

The 90% aq. MeOH extract (Section 4.2) of *G. cheloniae* CAWD232 was diluted to 60% aq. MeOH (1.23 L) before a second liquid–liquid partition with dichloromethane (DCM; 1:1) to separate the hydrophilic MTX-like and lipophilic P-CTX-like compounds. Both phases were collected and dried down using rotary evaporation (50 mBar/50 °C for 60% aq. MeOH and 300 mBar/40 °C for DCM). The dried 60% aq. MeOH phase was redissolved in Milli-Q water (30 mL). A Strata-X SPE column (10 g) was conditioned with EtOH, MeOH, and then Milli-Q water (200 mL of each), the sample was loaded in its entirety, washed with 40% aq. MeOH (200 mL), and eluted with 100% MeOH (200 mL).

The Strata-X SPE eluent (200 mL 100% MeOH) was dried down using rotary evaporation (50 mBar/50 °C) and redissolved in 20% aq. MeOH (5 mL). Fractionation of the extract was performed on a Reveleris flash chromatography system (Büchi) fitted with an Agilent Superflash C_18_ SF 25–75 g column (four injections) and two wavelengths were monitored, UV1 = 210 nm and UV2 = 230 nm. The column was eluted at 20 mL/min with (A) Milli-Q water and (B) MeCN mobile phases. The initial solvent composition was 20% B for 5 min before a linear gradient to 95% B from 5–30 min and then held at 95% B for 20 min. Fractions were collected every 30 s (10 mL; 99 fractions per injection) and combined as seven pooled fractions based on the UV2 trace (230 nm).

The two toxic fractions were combined, dried down under a stream of N_2_ gas at 50 °C, and redissolved in 30% aq. MeOH (1.5 mL). A second fractionation was performed on the Reveleris flash chromatography system (via a single injection) with the same column, mobile phases, flow rate, and monitored wavelengths. The initial solvent composition was 30% B for 5 min before a linear gradient to 95% B from 5–35 min, and then held at 95% B for 15 min. The resulting 99 fractions were combined as 4 pooled fractions based on the UV2 trace (230 nm).

The two toxic fractions were dried down under a stream of N_2_ gas at 50 °C and redissolved in 25% aq. MeOH (2 mL). A third fractionation was performed on a newer Reveleris X2 flash chromatography system (via a single injection) with the same column, mobile phases, flow rate, and monitored wavelengths. The initial solvent composition was 25% B for 5 min before a linear gradient to 55% B from 5–35 min, followed by a linear gradient to 95% B from 35–40 min, and then held at 95% B for 10 min. The resulting 99 fractions (from each injection) were combined as 3 and 4 pooled fractions based on the UV2 trace (230 nm).

The toxic fraction was further separated (via seven injections) on the Shimadzu preparative HPLC-PDA system with a Phenomenex Gemini C_18_ column (5 µm; 150 × 21.2 mm) and UV detection (190–300 nm). The column was eluted isocratically (25 mL/min) using 35% aq. MeCN mobile phase containing 0.2% (*v*/*v*) of a 25% NH_4_OH solution. The total injection time was 15 min with fractions manually collected every 30 s (12.5 mL; 30 fractions per injection). The resulting eluent was combined as six pooled fractions based on the LC–MS analysis.

The three most toxic fractions were combined, dried down under a stream of N_2_ gas at 50 °C, and redissolved in 100% MeOH (1.5 mL). The final fractionation (via ten injections) was performed using the same Shimadzu HPLC-PDA system, with the Gemini column and isocratic elution (25 mL/min) using 38% aq. MeCN mobile phases containing 0.2% (*v*/*v*) of a 25% NH_4_OH solution. The total injection time was 13 min with fractions manually collected every 30 s (12.5 mL; 26 fractions per injection). The resulting eluent was combined as nine pooled fractions based on the LC–MS analysis, with MTX-6 eluting in Fraction 5.

A flow diagram of the purification scheme is displayed in Figure S69.

### 4.4. Isolation of Maitotoxin-7

The 90% aq. MeOH extract (Section 4.2) of *G. honu* CAWD242 was diluted to 60% aq. MeOH (1.07 L), acidified with 0.05% acetic acid (*v*/*v*) before a second liquid–liquid partition with DCM (1:1, *v*/*v*) to separate the hydrophilic MTX-like and lipophilic P-CTX-like compounds. Both phases were collected and dried down using rotary evaporation (50 mBar/50 °C for 60% aq. MeOH and 300 mBar / 40 °C for DCM). The 60% aq. MeOH phase was redissolved in acidified Milli-Q water (25 mL; 0.05% acetic acid; *v*/*v*). A Strata-X SPE column (10 g) was conditioned with acidified EtOH, MeOH and then Milli-Q water, the sample was loaded in its entirety, washed with 60% aq. MeOH, and eluted with 100% MeOH (200 mL of each solution + 0.05% acetic acid; *v*/*v*).

The Strata-X SPE eluent (200 mL 100% MeOH) was dried down using rotary evaporation (50 mBar/50 °C) and redissolved in 20% aq. MeOH + 0.05% AA (*v*/*v*; 6 mL). Fractionation of the extract was performed on a Reveleris flash chromatography system fitted with an Agilent Superflash C_18_ SF 25–75 g column (three injections) and two wavelengths were monitored (UV1 = 210 nm and UV2 = 230 nm). The column was eluted at 20 mL/min with (A) Milli-Q water and (B) MeCN mobile phases, both with the addition of 0.05% AA (*v*/*v*). The initial solvent composition was 20% B for 5 min before a linear gradient to 95% B from 5–30 min and then held at 95% B for 10 min. Fractions were collected every 30 s (10 mL; 79 fractions per injection) and combined as 9 pooled fractions based on the UV2 trace (230 nm).

The toxic fraction (as determined by MBA) was dried down under a stream of N_2_ gas at 50 °C and redissolved in 38% aq. MeOH + 0.2% (*v*/*v*) of a 25% NH_4_OH solution (1 mL). Fractionation was performed (via six injections) using a Shimadzu preparative HPLC-PDA system with a Phenomenex Gemini C_18_ column (5 µm; 150 × 21.2 mm) and UV detection (190–300 nm). The column was eluted isocratically (25 mL/min) using 38% aq. MeCN mobile phase containing 0.2% (*v*/*v*) of a 25% NH_4_OH solution. The total injection time was 15 min. Fractions were manually collected every 30 s (12.5 mL; 30 fractions per injection) and combined as 13 pooled fractions based on LC–MS analysis

The toxic fraction (as determined by MBA) was dried down under a stream of N_2_ gas at 50 °C and redissolved in 35% aq. MeOH + 0.2% (*v*/*v*) of a 25% NH_4_OH solution (1 mL). The final fractionation (via five injections) was performed using the same Shimadzu HPLC-PDA system, with the Gemini column and isocratic elution (25 mL/min) using 35% aq. MeCN mobile phase containing 0.2% (*v*/*v*) of a 25% NH_4_OH solution. The total injection time was 12 min. Fractions were manually collected every 30 s (12.5 mL; 24 per injection) and combined as nine pooled fractions based on the LC–MS analysis, with MTX-7 eluting in Fraction 8.

A flow diagram of the purification scheme is displayed in Figure S70.

### 4.5. Liquid Chromatography–Mass Spectrometry

Analyses were performed on a Waters Xevo TQ-S triple quadrupole mass spectrometer coupled to a Waters Acquity UPLC i-Class with a flow-through needle sample manager. The mass spectrometer utilized electrospray ionization (ESI; positive and negative ion modes) and chromatographic separation was achieved on a Waters Acquity UPLC BEH phenyl column (1.7 μm, 100 × 2.1 mm) held at 50 °C. The column was eluted using mobile phases containing 0.2% (*v*/*v*) of a 25% NH_4_OH solution in (A) Milli-Q water and (B) MeCN and a flow rate of 0.55 mL/min. Initial solvent conditions were 5% B for 1 min with a linear gradient to 95% B from 1.0–7.5 min, held at 95% B for 1 min, followed by a linear gradient back to 5% B from 8.5−9 min. The column was re-equilibrated with 5% B until 10 min. Fresh mobile phases were prepared daily to ensure the optimal sensitivity and stable retention times. The autosampler chamber was maintained at 10 °C and the injection volume was 1 μL. Other settings were capillary voltage 3.0 kV, cone voltage 40 V, source temperature 150 °C, N_2_ gas desolvation flow rate 1000 L/h at 600 °C, cone gas 150 L/h, and the collision cell was operated with 0.15 mL/min argon. Data acquisition and processing were performed with MassLynx and TargetLynx software, respectively.

Scanning experiments were performed in both +ESI and −ESI modes with two ranges, low mass (*m*/*z* 200–1000) and high mass (*m*/*z* 800–1800). CID MS/MS experiments, with various scan ranges used between *m*/*z* 48–1800 (which were defined based on the precursor ion), were performed in both +ESI and −ESI modes. CEs ranged from 10 to 55 eV and 40 to 100 eV for +ESI and −ESI, respectively.

#### 4.5.1. Chemical Modification Experiments

##### Oxidative Cleavage

An aliquot (10 μL) of each MTX stock solution (either MTX-1, MTX-6, or MTX-7) was diluted with Milli-Q water (75 µL) and 50 mM of periodic acid (15 µL) was added. The reaction was left at room temperature for 2 h before LC–MS analysis.

##### Reduction

Post reaction (24 h), the periodic acid oxidative cleavage products were reduced with sodium borohydride. An aliquot of each oxidized MTX analog was taken (20 µL) and 100 mM of sodium borohydride (20 µL) was added. The reaction was left at room temperature for 2 h before LC–MS analysis.

##### Chromatographic Parameters

Chromatographic separation was achieved on a Thermo Scientific Hypersil Gold aQ column (1.9 μm, 50 × 2.1 mm) held at 40 °C. The column was eluted at 0.5 mL/min using mobiles phases containing 0.1% formic acid (*v*/*v*) in (A) Milli-Q water and (B) MeCN. The autosampler chamber was maintained at 10 °C and the injection volume was 1 μL. The reaction products (in both the oxidized and reduced states) were also analyzed using the BEH phenyl column and NH_4_OH mobile phases as described in Section 4.5. The initial solvent composition was 5% B with a linear gradient to 30% B from 0.5 to 3 min, ramped to 95% B by 3.5 min, held at 95% B until 4 min, and followed by a linear gradient back to 5% B at 4.5 min. The column was then re-equilibrated with 5% B until 5 min.

Scanning experiments were performed in both +ESI and −ESI modes with preliminary scan ranges of *m*/*z* 48–1000 and *m*/*z* 800–1800, followed by a narrow scan range of *m*/*z* 900−1100. CID fragmentation experiments were performed on the dominant precursor ions in −ESI with CEs ranging from 40 to 100 eV and a scan range of *m*/*z* 48–1000.

### 4.6. High-Resolution Mass Spectrometry

Analyses were performed on a Thermo Scientific Q Exactive HF Orbitrap mass spectrometer coupled to an Agilent 1200 Infinity LC system. The mass spectrometer utilized a heated ESI probe (HESI-II) and was calibrated from *m*/*z* 74−1622 according to the manufacturer’s specification using the Pierce LTQ Velos calibration solutions (Thermo Scientific). Chromatographic separation was achieved on a Phenomenex Kinetex F5 pentafluorophenyl column (1.7 µm, 100 × 2.1 mm) using gradient elution at 0.30 mL/min and mobiles phases containing 0.1% formic acid (*v*/*v*) in (A) Milli-Q water and (B) MeCN. The column was held at 40 °C. The initial solvent composition was 10% B with a linear gradient to 80% B from 0–18 min, ramped to 99% B by 18.1 min, and held at 99% B until 22 min, followed by a linear gradient back to 10% B at 22.5 min. The column was re-equilibrated with 10% B until 30 min. The autosampler was maintained at 10 °C and the injection volume was 5 µL. Other settings included positive and negative polarity switching with a spray voltage of 4.5 kV, sheath gas pressure of 40 psi, the auxiliary gas flow of 10 (arbitrary units), capillary temperature (340 °C), and heater temperature (150 °C).

Molecular formulae were determined with instrument parameters set with a mass tolerance of 10 ppm, ring double bond equivalence of 10–100, and the low/high atom composition thresholds were set for oxygen (0–70), carbon (0–170), hydrogen (0–270), and sulfur (0−2).

Initial scanning experiments were performed with two ranges, low mass (*m*/*z* 800−2000) and high mass (*m*/*z* 2500–3500). Each range had specific settings for the resolution (120,000 and 240,000), AGC target (1 × 10^6^ and 5 × 10^6^), and maximum injection time (100 ms and 200 ms), respectively.

Full scan data for the low mass range (*m*/*z* 800–2000) had a resolution setting of 120,000 with an AGC target of 1 × 10^6^ and a maximum injection time of 50 ms. Collision-induced dissociation parallel reaction monitoring (CID-PRM) experiments were selected for doubly- and triply-charged ions for MTX-1, MTX-6, and MTX-7 (Table 3) with an isolation window of 2.0 Da. CEs of 40, 60, and 80 eV were used. The MS resolution was 30,000 and the AGC target was 2 × 10^5^ with a maximum injection time of 512 ms.

#### Oxidative Cleavage

An aliquot of each MTX stock solution (MTX-1, MTX-6, or MTX-7; 10−30 μL) was dried under N_2_ gas and redissolved in 50% aq. MeCN (50 µL). Sodium periodate (50 mM; 2 µL) was added and the reaction mixture was left at room temperature for 2 h. The HR–MS scanning experiments were performed at approximately 2 and 18 h time points.

Full scan data were collected from *m*/*z* 500–2500 with the LC gradient and MS settings as described above (Section 4.6). The MS resolution was 120,000 and the AGC target was 1 × 10^5^ with a maximum injection time of 200 ms.

Data-dependent acquisition (DDA) was used to collect CID fragment ion scans of the three most abundant ions in the full scan acquisition at each cycle, with an inclusion list for the observed periodate oxidation cleavage products from the full scan acquisition (Table S6). The mass range for full scan acquisition was *m*/*z* 500–2500 with a resolution setting of 60,000, an AGC target of 1 × 10^5^, and a maximum injection time of 100 ms. CID fragment ion scans were acquired with an isolation window of 1.0 Da and CE of 60 eV unless otherwise specified (Table S6). The resolution was set to 120,000 with an AGC target of 2 × 10^5^ and a maximum injection time of 250 ms. DDA was collected in positive and negative ionization modes via separate analyses.

### 4.7. Nuclear Magnetic Resonance Spectrometry

Both MTX-6 and MTX-7 were dried under a stream of N_2_ gas at 50 °C, redissolved in CD_3_OD (600 µL, >99.8% deuterium), and transferred to a Wilmad^®^ 5 mm 800 MHz high precision NMR tube.

A Bruker AVIII-HD 800 MHz spectrometer, equipped with a TCI ^1^H/^13^C/^15^N cryoprobe, was used to acquire ^1^H, homonuclear decoupled ^1^H, COSY, TOCSY, NOESY, ROESY, HSQC, HMBC, SHSQC, SHMBC, and DEPT135Q spectra, along with a series of higher-resolution 20, 40, 80, and 160 msec 1D-SELTOCSY, SELROESY, and SELNOESY spectra at 300 K. Bruker supplied Topspin pulse programs were modified in the case of COSY, TOCSY, NOESY, ROESY, SELTOCSY, SELNOESY, and SELROESY experiments with ES and/or CW presaturation of the HOD and/or methanol lines during the interpulse train delay period. Presaturation was applied at O1 on the F1 channel or via the F2 channel, independent of the setup of the F1 channel. NMR signal assignments were reported relative to CHD_2_OD (3.31 ppm) and CD_3_OD (49.0 ppm). Data integration and interpretation were performed using Bruker TopSpin software version 4.1.3. ^1^H NMR signal assignments of methyl groups were reported to three decimal places, where necessary for differentiation, while other proton signals were reported to two decimal places. ^13^C shifts were either directly determined via a DEPT135Q experiment, or indirectly determined in HSQC, HMBC, SHSQC, and/or SHMBC experiments. ^13^C methyl group chemical shifts were reported to two decimal places, which enabled them to be differentiated, while other carbon signals were reported to one decimal place.

Specific method acquisition and processing conditions can be provided on request.

### 4.8. Mouse Bioassay

#### 4.8.1. Animals

Female Swiss albino mice (18–22 g) were bred at AgResearch, Ruakura, New Zealand. The mice were housed individually during the experiments and were allowed unrestricted access to food (Rat and Mouse Cubes, Specialty Feeds Ltd., Glen Forrest, Western Australia) and water. All dosing was conducted between 8 and 9.30 a.m. to avoid any diurnal variations in response.

#### 4.8.2. Acute Toxicity by Intraperitoneal Injection

Acute toxicities were determined according to the principles of OECD guideline 425 [47]. The guideline employs an up-and-down procedure whereby one animal is given a dose of the test compound at one step below the estimated LD_50_. If this animal survives, the dose for the next animal is increased by a factor determined by the software program associated with the guideline [50]. This factor is determined from an estimate of the gradient of the dose–response curve. If the initial animal dies, the dose for the next animal is decreased by the same factor. Dosing is continued until four live-death reversals have been achieved.

Each mouse was weighed prior to dosing and the quantity of the test compound calculated to yield a dose rate on a mg/kg basis. Each dose was prepared by using the required volume of stock solution (the isolated MTX analog in 90% aq. MeOH), dried under N_2_ gas at 40 °C, and redissolved in 1% Tween 60 in normal saline (1 mL). The freshly prepared dose was immediately injected into the test mice. Mice were monitored closely during the day of dosing and those that survived were monitored for a 14-day observation period, which included daily measurements of food consumption and bodyweight. After 14 days, the mice were euthanized by CO_2_ inhalation and necropsied. The weights of the livers, kidneys, spleens, hearts, lungs, stomachs (full and empty), and the whole guts were measured and calculated as a percentage of bodyweight (data not included).

#### 4.8.3. Acute Toxicity by Oral Administration

The ‘over the tongue’ method was used to assess the oral toxicity of MTX-6 and MTX-7, with each dose calculated on a mg/kg basis. A ‘test solution’ was prepared that contained the required quantity of the respective purified secondary metabolite in water. A semi-solid paste was then made that contained ground mouse food (20 mg) and the ‘test solution’ (50 µL). The paste was applied over the tongue of the test mouse, with care taken to ensure the entire dose had been swallowed. Mice were weighed immediately prior to dosing and monitored closely during the day of dosing and for a 14-day observation period, which included a daily measurement of food consumption and bodyweight. After 14 days, the mice were euthanized by CO_2_ inhalation and necropsied [51]. The weights of the livers, kidneys, spleens, hearts, lungs, stomachs (full and empty), and the whole guts were measured and calculated as a percentage of bodyweight (data not included).

## 5. Conclusions

Two new MTX analogs, MTX-6 from *G. cheloniae* CAWD232 and MTX-7 from *G. honu* CAWD242, were isolated and described. Extensive analytical chemistry experiments were performed to characterize the structures, with MTX-7 being the most well-characterized compound of this class since the structure of MTX-1 was published in 1993. However, further research is required to elucidate the full structure. Both MTX-6 and MTX-7 displayed high toxicity to mice by i.p. injection, and as such, were identified as the primary compounds responsible for the observed i.p. toxicity of these *Gambierdiscus* species. Neither MTX analog displayed any oral potency at the highest dose rate administered, suggesting that these compounds are unlikely to play a major role in CP events.

## Figures and Tables

**Table 1:** Molecular composition of MTX-6 and MTX-1 [23], with the net atom changes and hypothesized chemical modifications.

	C	H	O	S
MTX-1	164	258	68	2
MTX-6	164	256	66	1
Net atom change *^a^*	-	−2	−2	−1
Sulfite loss			−3	−1
One additional hydroxyl group			1	
Double bond		−2		

*^a^* Compared to MTX-1.

**Table 2:** Molecular compositions of MTX-7 and MTX-1 [23], with the net atom changes and hypothesized chemical modifications.

	C	H	O	S
MTX-1	164	258	68	2
MTX-7	165	258	67	1
Net atom change *^a^*	1	-	−1	−1
Sulfite loss			−3	−1
Additional methyl group	1	2		
Two additional hydroxyl groups			2	
Double bond		−2		

*^a^* Compared to MTX-1.

**Table 3:** The doubly- and triply-charged ions used for the CID-PRM experiments.

	MTX-1	MTX-6	MTX-7
Doubly-charged ions	1690.8239	1657.8326	1655.8267 1672.8365
Triply-charged ions		1067.2311 1093.5519	1077.8365

MTX = Maitotoxin.

## Supplementary Materials

The following supporting information can be downloaded at: https://www.mdpi.com/article/10.3390/md20070453/s1. **Figure S1.** Full scan −ESI mass spectra of (**A**) MTX 1, (**B**) MTX 6 and (**C**) MTX 7 (*m*/*z* 800–1800) showing the [M–2H]^2−^ (*m*/*z* 1689.4, 1656.3 and 1671.4), [M–3H]^3−^ (*m*/*z* 1126.1, 1104.1 and 1114.1) and [M−4H]^4−^ (*m*/*z* 844.4, 827.8 and 835.4) ions, respectively; **Figure S2.** Full scan +ESI mass spectra of (**A**) MTX 1, (**B**) MTX 6 and (**C**) MTX 7 (*m*/*z* 800–1800) showing the doubly-charged (*m*/*z* 1594.0, 1600.0 and 1615.5) and triply charged (*m*/*z* 1062.8, 1073.0 and 1077.5) ions, respectively; **Figure S3.** Lower mass range CID spectra of the dominant trianions [M-3H]^3−^ of (**A**) MTX 1, (**B**) MTX 6 and (**C**) MTX 7 (60 eV CE; *m*/*z* 50–310); **Figure S4.** Higher mass range CID spectra of the dominant trianions [M-3H]^3−^ of (**A**) MTX 1, (**B**) MTX 6 and (**C**) MTX 7 (60 eV CE; *m*/*z* 700–1100); **Figure S5.** High-resolution full scan mass spectrum of MTX 1 in +ESI mode showing the singly-charged cations (Δ +2.8 ppm). Note: this is outside the calibration range of the instrument; **Figure S6.** High-resolution full scan mass spectrum of MTX 1 in +ESI mode showing the triply-charged cations (Δ −0.14 ppm); **Figure S7.** High-resolution full scan mass spectrum of MTX 1 in −ESI mode showing a singly-charged anion (Δ −7.13 ppm). Note: this is outside the calibration range of the instrument; **Figure S8.** High-resolution full scan mass spectrum of MTX 1 in −ESI mode showing a doubly-charged anion (Δ +1.49 ppm); **Figure S9.** High-resolution full scan mass spectrum of MTX 1 in −ESI mode showing the triply-charged anions (Δ −1.59 ppm); **Figure S10.** High-resolution mass spectrum of the doubly charged cations of MTX 1, displaying the *m*/*z* range 1540−1760; **Figure S11.** (**A**) Observed isotope distribution of the MTX 1 [M+2H]^2+^ ion, compared to (**B**) the theoretical isotope distribution calculated using the NRCC Molecular Formula Calculator v1.01; **Figure S12.** High-resolution full scan mass spectrum of MTX 6 in +ESI mode showing the singly-charged cations. Note: this is outside the calibration range of the instrument; **Figure S13.** High-resolution full scan mass spectrum of MTX-6 in +ESI mode showing the triply-charged cations; **Figure S14.** Hig- resolution full scan mass spectrum of MTX-7 in +ESI mode showing the singly-charged cations. Note: this is outside the calibration range of the instrument; **Figure S15.** High-resolution full scan mass spectrum of MTX-7 in +ESI mode showing the triply-charged cations; **Figure S16.** High-resolution full scan mass spectrum of MTX-6 in −ESI mode showing a singly-charged anion. Note: this is outside the calibration range of the instrument; **Figure S17.** High-resolution full scan mass spectrum of MTX-7 in −ESI mode showing a singly-charged anion. Note: this is outside the calibration range of the instrument; **Figure S18.** High-resolution full scan mass spectrum of MTX-6 in −ESI mode showing the doubly-charged anions; **Figure S19.** High-resolution full scan mass spectrum of MTX-6 in −ESI mode showing the triply-charged anions; **Figure S20.** High-resolution full scan mass spectrum of MTX-7 in −ESI mode showing the doubly-charged anions; **Figure S21.** High-resolution full scan mass spectrum of MTX-7 in −ESI mode showing the triply-charged anions; **Figure S22.** (**A**) Observed isotope distribution of the MTX-6 cation [M+H]^+^, compared to (**B**) the theoretical isotope distribution calculated using the NRCC Molecular Formula Calculator v1.01; **Figure S23.** Expanded view of (**A**) [M+2H]^2+^ and (**B**) [M–2H]^2−^, of MTX-7, displaying the signals from the single (most intense) and double (minor) molecular ions; **Figure S24.** (**A**) Observed isotope distribution of the MTX-7 doubly-charged formate adduct, compared to (**B**) the theoretical isotope distribution calculated using the NRCC Molecular Formula Calculator v1.01; **Figure S25.** Full scan −ESI mass spectra of the oxidative cleavage products of the (**A**) peak at 1.31 min and (**B**) the peak at 1.35 min observed in MTX-1 and MTX-7. The peaks observed at (**C**) 1.36 min and (**D**) 1.39 min in MTX-6 are also included; **Figure S26.** Proposed structures of the products of periodate oxidation of MTX-1 and MTX-7 pertaining to the common ions observed in the spectra of the compounds; **Figure S27.** Full scan −ESI mass spectra of the oxidative cleavage products of the (**A**) peak at 1.37 min and (**B**) peak at 1.39 min observed in MTX-1 and MTX-7. The peak observed at (**C**) 1.42 min in MTX-6 (acquired using a BEH phenyl column and ammoniated mobile phases) is also included; **Figure S28.** CID of the dominant oxidation cleavage products (**A**) *m*/*z* 971.2 at 1.31 min and (**B**) *m*/*z* 985.3 at 1.35 min for MTX-1 using 60 eV CE; **Figure S29.** CID of the dominant oxidation cleavage products (**A**) *m*/*z* 955.3 at 1.36 min and (**B**) *m*/*z* 969.3 at 1.39 min for MTX-6 using 60 eV CE; **Figure S30.** CID of the dominant oxidation cleavage products (**A**) *m*/*z* 971.2 at 1.31 min and (**B**) *m*/*z* 985.3 at 1.35 min for MTX-7 using 60 eV CE; **Figure S31.** The proposed CID fragmentation of Fragment A in −ESI (60 eV CE; *m*/*z* 50–1000) and comparison of the corresponding spectra of (**A**) MTX-1, (**B**) MTX-6 and (**C**) MTX-7. Green lines indicate the fragments that are 2 Da higher in mass for MTX-6 compared to MTX-1 and MTX-7. Red lines indicate the fragments that align between the three MTX analogs; **Figure S32.** Full scan +ESI HR mass spectra (*m*/*z* 800–2500) of the oxidative cleavage products of MTX-1 displaying (**A**) Fragment A, (**B**) Fragment B and (**C**) Fragments B and C connected; **Figure S33.** Full scan +ESI HR mass spectra (*m*/*z* 800–2500) of the oxidative cleavage products of MTX-6 displaying (**A**) ‘Fragment A’, (**B**) ‘Fragment B’ and (**C**) ‘Fragments B and C’ connected; **Figure S34.** CID fragmentation spectra of the two unknown ions (**A**) *m*/*z* 1665.9308 and (**B**) *m*/*z* 1551.8261 of MTX-6, using 40 eV CE; **Figure S35.** Full scan +ESI HR mass spectra (*m*/*z* 800–2500) of the oxidative cleavage products of MTX-7 displaying (**A**) Fragment A, (**B**) ‘Fragment B’ and (**C**) ‘Fragments B and C’ connected; **Figure S36.** CID fragmentation spectra of the two unknown ions (**A**) *m*/*z* 1709.9563 and (**B**) *m*/*z* 1567.8214 of MTX-7, using 40 eV CE; **Figure S37.** A comparison of the CID fragmentation spectra (40 eV CE) for the B fragments of (**A**) MTX-6 *m*/*z* 1551.8253 and (**B**) MTX-7 *m*/*z* 1567.8208; **Figure S38.** Fragmentation spectra of the *m*/*z* 823.4481 ions (C_43_H_67_O_15_) from (**A**) MTX-1, (**B**) MTX-6 and (**C**) MTX-7; **Figure S39.** Reduced oxidative cleavage products of the (**A**) peak at 1.90 min, (**B**) peak at 1.92 min in MTX-1 and MTX-7, and (**C**) the peak in MTX-6 at 1.96 min; **Figure S40.** The (**A**) Fragment A oxidation product of MTX-1 and MTX-7 and the proposed structures for the reduced forms pertaining to the two unresolved peaks at (**B**) 1.90 min and (**C**) 1.92 min (one of the three hydroxyls in blue is an aldehyde); **Figure S41.** ^1^H NMR spectrum of MTX-7 acquired in CD_3_OD at 800 MHz; **Figure S42.** Expansion of the ^1^H NMR spectrum of MTX 7 displaying (**A**) the upfield methyl groups (0.8–1.5 ppm) and (**B**) the downfield olefinic proton signals (4.9–5.9 ppm) acquired in CD_3_OD at 800 MHz; **Figure S43.** Expansion of the ^1^H NMR spectrum of MTX-7 (3.5–4.5 ppm region) acquired in CD_3_OD at 800 MHz; **Figure S44.** DEPT135Q NMR spectrum of MTX-7 acquired in CD_3_OD at 200 MHz; **Figure S45.** Expansion of the 8–42 ppm region of the DEPT135Q spectrum of MTX-7 acquired in CD_3_OD at 200 MHz; **Figure S46.** Expansion of the SHSQC spectrum (^1^H: 0.8–1.8 ppm; ^13^C: 10−30 ppm) showing the correlations determined for the 22 methyl groups (numbered according to their chemical shift, starting upfield) present in MTX-7 acquired in CD_3_OD at 800 MHz; **Figure S47.** Expansion of the SHSQC spectrum (^1^H: 1.2–2.8 ppm; ^13^C: 25–55 ppm) of MTX-7 acquired in CD_3_OD at 800 MHz showing the methylene proton region, including the 2.78 ppm and 2.27 ppm proton signals proposed to originate from the H-118_a,b_ protons of a ring B′ (*Z*)-double bond, analogous to that published by Murata et al., for MTX-1; **Figure S48.** Expansion of the COSY spectrum (^1^H: 0.8–1.05 ppm; ^1^H: 0.8−2.2 ppm) of MTX-7 acquired in CD_3_OD at 800 MHz depicting the methine proton correlations to the five secondary methyl groups; **Figure S49.** Expansion of the SHSQC spectrum (^1^H: 2.9–4.5 ppm; ^13^C: 62−88 ppm) of MTX-7 acquired in CD_3_OD at 800 MHz showing correlations arising from the oxygenated methine signals; **Figure S50.** Expansion of the COSY spectrum (^1^H: 4.7–5.8 ppm; ^1^H: 4.9−5.8 ppm) of MTX-7 acquired in CD_3_OD at 800 MHz showing the five sets of olefinic signals (individual protons or groups), with assignments based on those published for MTX-1 by Murata et al.; **Figure S51.** Expansion of the HSQC spectrum (^1^H: 4.9–6.0 ppm; ^13^C: 100−150 ppm) of MTX-7 acquired in CD_3_OD at 800 MHz showing the five individual or pairs of olefinic protons, with assignments based on those published for MTX-1 by Murata et al.; **Figure S52.** COSY NMR spectrum of MTX-7 acquired in CD_3_OD at 800 MHz; **Figure S53.** HMBC NMR spectrum of MTX-7 acquired in CD_3_OD at 800 MHz; **Figure S54.** Expansion of the HMBC NMR spectrum of MTX-7 (^1^H: 0.8–1.8 ppm; ^13^C: 20–140 ppm) acquired in CD_3_OD at 800 MHz; **Figure S55.** HSQC NMR spectrum of MTX-7 acquired in CD_3_OD at 800 MHz; **Figure S56.** NOESY NMR spectrum of MTX-7 acquired in CD_3_OD at 800 MHz; **Figure S57.** ROESY NMR spectrum of MTX-7 acquired in CD_3_OD at 800 MHz; **Figure S58.** TOCSY NMR spectrum of MTX-7 acquired in CD_3_OD at 800 MHz; **Figure S59.** 160 msec 1D-SELTOCSY spectra used to assign the three-ring A secondary methyl groups (**A**) 0.936 ppm (14-CH_3_), (**B**) 0.979 ppm (12-CH_3_) and (**C**) 1.031 ppm (7-CH_3_); **Figure S60.** (**A**) 3.13 ppm H-135 slice from the 160 msec 2D TOCSY spectrum, and the 160 msec 1D SELTOCSY spectra acquired for the two ring F′ secondary methyl groups (**B**) 139 CH_3_ (0.863 ppm) and (**C**) 138 CH_3_ (0.900 ppm) in MTX-7 acquired in CD_3_OD at 800 MHz; **Figure S61.** A NOESY slice ex the 138-CH_3_ (0.900 ppm) methyl group protons of MTX-7 acquired in CD_3_OD at 800 MHz showing correlations to H-134 (3.92 ppm), H-135 (3.31 ppm), H-136 (3.70 ppm), H-137_a,b_ (1.20, 1.56 ppm), H-138 (1.76 ppm) and H-139 (1.48 ppm) protons; **Figure S62.** Expansion of the NOESY spectrum (^1^H: 1.15–1.50 ppm; ^1^H: 1.15−1.50 ppm) of MTX-7 acquired in CD_3_OD at 800 MHz displaying the three sets of mutual NOESY correlations pertaining to the three methyl groups located in rings C′–F′. NMR chemical shifts are calibrated relative to CHD_2_OD (3.31 ppm); **Figure S63.** ^1^H NMR spectrum of MTX-6 acquired in CD_3_OD at 800 MHz; **Figure S64.** COSY NMR spectrum of MTX-6 acquired in CD_3_OD at 800 MHz; **Figure S65.** TOCSY NMR spectrum of MTX-6 acquired in CD_3_OD at 800 MHz; **Figure S66.** HSQC NMR spectrum of MTX-6 acquired in CD_3_OD at 800 MHz; **Figure S67.** Stacked ^1^H NMR spectra (4.6–5.8 ppm) of (**A**) MTX-7 and (**B**) MTX-6 acquired in CD_3_OD at 800 MHz showing the five sets of olefinic proton signals; H-2, 4=CH_2_ (H-144_a,b_), H-119 and H-120 (ring B’), H-141 and H-142_a,b_; **Figure S68.** Expansion of the HSQC spectrum (^1^H: 4.9–6.0 ppm; ^13^C: 100–150 ppm) of MTX-6 acquired in CD_3_OD at 800 MHz showing the five individual or pairs of olefinic protons, with assignments based on those defined for MTX-7 (Figure S52). NMR chemical shifts are calibrated relative to CHD_2_OD (3.31 ppm) and CD_3_OD (49.0 ppm); **Figure S69.** Purification scheme of maitotoxin-6; **Figure S70.** Purification scheme of maitotoxin-7; **Table S1.** The ^1^H and ^13^C NMR chemical shifts (ppm), multiplicity and coupling constants (Hz) of the 22 methyl groups (numbering according to their chemical shift, starting upfield) observed in MTX-7. Spectra were acquired in CD_3_OD using a 800 MHz spectrometer; **Table S2.** The ^1^H and ^13^C NMR chemical shifts (ppm; 800 MHz) of the five methine groups and the respective secondary methyl groups (the chemical shift corresponds to the underlined atom). Spectra were acquired in CD_3_OD using a 800 MHz spectrometer; **Table S3.** The ^1^H and ^13^C NMR chemical shifts (ppm) of the aliphatic side-chain atoms connected to ring A in MTX-7. Spectra were acquired in CD_3_OD using a 800 MHz spectrometer; **Table S4.** The ^1^H and ^13^C NMR chemical shifts (ppm) of the aliphatic side-chain atoms connected to ring F′ in MTX 7. Spectra were acquired in CD_3_OD using a 800 MHz spectrometer; **Table S5.** The ^1^H and ^13^C NMR chemical shifts (ppm) and the HMBC ^13^C correlations observed for the 22 methyl groups (numbered according to their chemical shift, starting upfield) in MTX-7. Spectra were acquired in CD_3_OD using a 800 MHz spectrometer; **Table S6.** An inclusion list of the observed periodate oxidation fragment ions for MTX-1, MTX-6 and MTX-7 that were used for the CID experiments.
