# Supplementary material for: Structural Characterization of Maitotoxins Produced by Toxic Gambierdiscus Species
Source: Mar Drugs. 2022 Jul 12;20(7):453. doi: 10.3390/md20070453 (PMC9324523; doi:10.3390/md20070453)
Supplement: Supplementary file 1 [file marinedrugs-20-00453-s001.zip › marinedrugs-1798717-supplementary.pdf]

Supplementary information for

Structural characterization of maitotoxins produced by toxic *Gambierdiscus* species

---

J. Sam Murray,* Sarah C. Finch, Elizabeth M. Mudge, Alistair L. Wilkins, Jonathan Puddick, D. Tim Harwood, Lesley L. Rhodes, Roel van Ginkel, Frode Rise and Michèle R. Prinsep

* Corresponding author – sam.murray@Cawthron.org.nz

Figure S1. Full scan –ESI mass spectra of (**A**) MTX 1, (**B**) MTX 6 and (**C**) MTX 7 (*m/z* 800–1,800) showing the $[M–2H]^{2-}$ (*m/z* 1,689.4, 1,656.3 and 1,671.4), $[M–3H]^{3-}$ (*m/z* 1,126.1, 1,104.1 and 1,114.1) and $[M−4H]^{4-}$ (*m/z* 844.4, 827.8 and 835.4) ions, respectively 7

Figure S2. Full scan +ESI mass spectra of (**A**) MTX 1, (**B**) MTX 6 and (**C**) MTX 7 (*m/z* 800–1,800) showing the doubly-charged (*m/z* 1,594.0, 1,600.0 and 1,615.5) and triply charged (*m/z* 1,062.8, 1,073.0 and 1,077.5) ions, respectively 7

Figure S3. Lower mass range CID spectra of the dominant trianions $[M\text{-}3H]^{3-}$ of (**A**) MTX 1, (**B**) MTX 6 and (**C**) MTX 7 (60 eV CE; *m/z* 50−310) 8

Figure S4. Higher mass range CID spectra of the dominant trianions $[M\text{-}3H]^{3-}$ of (**A**) MTX 1, (**B**) MTX 6 and (**C**) MTX 7 (60 eV CE; *m/z* 700−1,100) 8

Figure S5. High resolution full scan mass spectrum of MTX 1 in +ESI mode showing the singly-charged cations (Δ +2.8 ppm). Note: this is outside the calibration range of the instrument 9

Figure S6. High resolution full scan mass spectrum of MTX 1 in +ESI mode showing the triply-charged cations (Δ –0.14 ppm) 9

Figure S7. High resolution full scan mass spectrum of MTX 1 in –ESI mode showing a singly-charged anion (Δ –7.13 ppm). Note: this is outside the calibration range of the instrument 10

Figure S8. High resolution full scan mass spectrum of MTX 1 in –ESI mode showing a doubly-charged anion (Δ +1.49 ppm) 10

Figure S9. High resolution full scan mass spectrum of MTX 1 in –ESI mode showing the triply-charged anions (Δ –1.59 ppm) 11

Figure S10. High resolution mass spectrum of the doubly charged cations of MTX 1, displaying the *m/z* range 1,540−1,760 11

Figure S11. (**A**) Observed isotope distribution of the MTX 1 $[M+2H]^{2+}$ ion, compared to (**B**) the theoretical isotope distribution calculated using the NRCC Molecular Formula Calculator v1.01 12

Figure S12. High resolution full scan mass spectrum of MTX 6 in +ESI mode showing the singly-charged cations. Note: this is outside the calibration range of the instrument 13

Figure S13. High resolution full scan mass spectrum of MTX-6 in +ESI mode showing the triply-charged cations 13

Figure S14. High resolution full scan mass spectrum of MTX-7 in +ESI mode showing the singly-charged cations. Note: this is outside the calibration range of the instrument 14

Figure S15. High resolution full scan mass spectrum of MTX-7 in +ESI mode showing the triply-charged cations 14

Figure S16. High resolution full scan mass spectrum of MTX-6 in –ESI mode showing a singly-charged anion. Note: this is outside the calibration range of the instrument 15

Figure S17. High resolution full scan mass spectrum of MTX-7 in –ESI mode showing a singly-charged anion. Note: this is outside the calibration range of the instrument 15

Figure S18. High resolution full scan mass spectrum of MTX-6 in –ESI mode showing the doubly-charged anions 16

Figure S19. High resolution full scan mass spectrum of MTX-6 in –ESI mode showing the triply-charged anions 16

Figure S20. High resolution full scan mass spectrum of MTX-7 in –ESI mode showing the doubly-charged anions ... 17
Figure S21. High resolution full scan mass spectrum of MTX-7 in –ESI mode showing the triply-charged anions ... 17
Figure S22. (**A**) Observed isotope distribution of the MTX-6 cation $[M+H]^+$, compared to (**B**) the theoretical isotope distribution calculated using the NRCC Molecular Formula Calculator v1.01 ... 18
Figure S23. Expanded view of (**A**) $[M+2H]^{2+}$ and (**B**) $[M–2H]^{2-}$, of MTX-7, displaying the signals from the single (most intense) and double (minor) molecular ions ... 19
Figure S24. (**A**) Observed isotope distribution of the MTX-7 doubly-charged formate adduct, compared to (**B**) the theoretical isotope distribution calculated using the NRCC Molecular Formula Calculator v1.01 ... 20
Figure S25. Full scan –ESI mass spectra of the oxidative cleavage products of the (**A**) peak at 1.31 min and (**B**) the peak at 1.35 min observed in MTX-1 and MTX-7. Also included are the peaks observed at (**C**) 1.36 min and (**D**) 1.39 min in MTX-6 ... 20
Figure S26. Proposed structures of the products of periodate oxidation of MTX-1 and MTX-7 pertaining to the common ions observed in the spectra of the compounds ... 21
Figure S27. Full scan –ESI mass spectra of the oxidative cleavage products of the (**A**) peak at 1.37 min and (**B**) peak at 1.39 min observed in MTX-1 and MTX-7. Also included is the peak observed at (**C**) 1.42 min in MTX-6 (acquired using a BEH phenyl column and ammoniated mobile phases) ... 22
Figure S28. CID of the dominant oxidation cleavage products (**A**) *m/z* 971.2 at 1.31 min and (**B**) *m/z* 985.3 at 1.35 min for MTX-1 using 60 eV CE ... 22
Figure S29. CID of the dominant oxidation cleavage products (**A**) *m/z* 955.3 at 1.36 min and (**B**) *m/z* 969.3 at 1.39 min for MTX-6 using 60 eV CE ... 23
Figure S30. CID of the dominant oxidation cleavage products (**A**) *m/z* 971.2 at 1.31 min and (**B**) *m/z* 985.3 at 1.35 min for MTX-7 using 60 eV CE ... 23
Figure S31. The proposed CID fragmentation of Fragment A in –ESI (60 eV CE; *m/z* 50−1,000) and comparison of the corresponding spectra of (**A**) MTX-1, (**B**) MTX-6 and (**C**) MTX-7. Green lines indicate the fragments that are 2 Da higher in mass for MTX-6 compared to MTX-1 and MTX-7. Red lines indicate the fragments that align between the three MTX analogues ... 24
Figure S32. Full scan +ESI HR mass spectra (*m/z* 800–2,500) of the oxidative cleavage products of MTX-1 displaying (**A**) Fragment A, (**B**) Fragment B and (**C**) Fragments B and C connected ... 25
Figure S33. Full scan +ESI HR mass spectra (*m/z* 800–2,500) of the oxidative cleavage products of MTX-6 displaying (**A**) ‘Fragment A’, (**B**) ‘Fragment B’ and (**C**) ‘Fragments B and C’ connected ... 26
Figure S34. CID fragmentation spectra of the two unknown ions (**A**) *m/z* 1,665.9308 and (**B**) *m/z* 1,551.8261 of MTX-6, using 40 eV CE ... 27
Figure S35. Full scan +ESI HR mass spectra (*m/z* 800–2,500) of the oxidative cleavage products of MTX-7 displaying (**A**) Fragment A, (**B**) ‘Fragment B’ and (**C**) ‘Fragments B and C’ connected ... 28
Figure S36. CID fragmentation spectra of the two unknown ions (**A**) *m/z* 1,709.9563 and (**B**) *m/z* 1,567.8214 of MTX-7, using 40 eV CE ... 29

Figure S37. Comparison of the CID fragmentation spectra (40 eV CE) for the B fragments of (**A**) MTX-6 *m/z* 1,551.8253 and (**B**) MTX-7 *m/z* 1,567.8208 30

Figure S38. Fragmentation spectra of the *m/z* 823.4481 ions ($C_{43}H_{67}O_{15}$) from (**A**) MTX-1, (**B**) MTX-6 and (**C**) MTX-7 31

Figure S39. Reduced oxidative cleavage products of the (**A**) peak at 1.90 min, (**B**) peak at 1.92 min in MTX-1 and MTX-7, and (**C**) the peak in MTX-6 at 1.96 min 31

Figure S40. The (**A**) Fragment A oxidation product of MTX-1 and MTX-7 and the proposed structures for the reduced forms pertaining to the two unresolved peaks at (**B**) 1.90 min and (**C**) 1.92 min (one of the three hydroxyls in blue is an aldehyde) 32

Figure S41. ^{1}H NMR spectrum of MTX-7 acquired in CD_3OD at 800 MHz 32

Figure S42. Expansion of the ^{1}H NMR spectrum of MTX 7 displaying (**A**) the upfield methyl groups (0.8−1.5 ppm) and (**B**) the downfield olefinic proton signals (4.9–5.9 ppm) acquired in CD_3OD at 800 MHz. 33

Figure S43. Expansion of the ^{1}H NMR spectrum of MTX-7 (3.5–4.5 ppm region) acquired in CD_3OD at 800 MHz. 34

Figure S44. DEPT135Q NMR spectrum of MTX-7 acquired in CD_3OD at 200 MHz........ 34

Figure S45. Expansion of the 8–42 ppm region of the DEPT135Q spectrum of MTX-7 acquired in CD_3OD at 200 MHz. 36

Figure S46. Expansion of the SHSQC spectrum (^{1}H: 0.8–1.8 ppm; ^{13}C: 10−30 ppm) showing the correlations determined for the 22 methyl groups (numbered according to their chemical shift, starting upfield) present in MTX-7 acquired in CD_3OD at 800 MHz........ 36

Figure S47. Expansion of the SHSQC spectrum (^{1}H: 1.2–2.8 ppm; ^{13}C: 25–55 ppm) of MTX-7 acquired in CD_3OD at 800 MHz showing the methylene proton region, including the 2.78 ppm and 2.27 ppm proton signals proposed to originate from the H-$118_{a,b}$ protons of a ring B′ (*Z*)-double bond, analogous to that published by Murata *et al.*,[1,2] for MTX-1........ 37

Figure S48. Expansion of the COSY spectrum (^{1}H: 0.8–1.05 ppm; ^{1}H: 0.8−2.2 ppm) of MTX-7 acquired in CD_3OD at 800 MHz depicting the methine proton correlations to the five secondary methyl groups. 37

Figure S49. Expansion of the SHSQC spectrum (^{1}H: 2.9–4.5 ppm; ^{13}C: 62−88 ppm) of MTX-7 acquired in CD_3OD at 800 MHz showing correlations arising from the oxygenated methine signals 38

Figure S50. Expansion of the COSY spectrum (^{1}H: 4.7–5.8 ppm; ^{1}H: 4.9−5.8 ppm) of MTX-7 acquired in CD_3OD at 800 MHz showing the five sets of olefinic signals (individual protons or groups), with assignments based on those published for MTX-1 by Murata *et al.*[2] 39

Figure S51. Expansion of the HSQC spectrum (^{1}H: 4.9–6.0 ppm; ^{13}C: 100−150 ppm) of MTX-7 acquired in CD_3OD at 800 MHz showing the five individual or pairs of olefinic protons, with assignments based on those published for MTX-1 by Murata *et al.*[2] 39

Figure S52. COSY NMR spectrum of MTX-7 acquired in CD_3OD at 800 MHz........ 40

Figure S53. HMBC NMR spectrum of MTX-7 acquired in CD_3OD at 800 MHz........ 40

Figure S54. Expansion of the HMBC NMR spectrum of MTX-7 (^{1}H: 0.8–1.8 ppm; ^{13}C: 20–140 ppm) acquired in CD_3OD at 800 MHz 41

Figure S55. HSQC NMR spectrum of MTX-7 acquired in CD_3OD at 800 MHz 41
Figure S56. NOESY NMR spectrum of MTX-7 acquired in CD_3OD at 800 MHz 42
Figure S57. ROESY NMR spectrum of MTX-7 acquired in CD_3OD at 800 MHz 42
Figure S58. TOCSY NMR spectrum of MTX-7 acquired in CD_3OD at 800 MHz 43
Figure S59. 160 msec 1D-SELTOCSY spectra used to assign the three ring A secondary methyl groups (**A**) 0.936 ppm (14-CH_3), (**B**) 0.979 ppm (12-CH_3) and (**C**) 1.031 ppm (7-CH_3) 43
Figure S60. (**A**) 3.13 ppm H-135 slice from the 160 msec 2D TOCSY spectrum, and the 160 msec 1D SELTOCSY spectra acquired for the two ring F′ secondary methyl groups (**B**) 139 CH_3 (0.863 ppm) and (**C**) 138 CH_3 (0.900 ppm) in MTX-7 acquired in CD_3OD at 800 MHz 45
Figure S61. A NOESY slice ex the 138-CH_3 (0.900 ppm) methyl group protons of MTX-7 acquired in CD_3OD at 800 MHz showing correlations to H-134 (3.92 ppm), H-135 (3.31 ppm), H-136 (3.70 ppm), H-$137_{a,b}$ (1.20, 1.56 ppm), H-138 (1.76 ppm) and H-139 (1.48 ppm) protons........ 45
Figure S62. Expansion of the NOESY spectrum (^{1}H: 1.15–1.50 ppm; ^{1}H: 1.15−1.50 ppm) of MTX-7 acquired in CD_3OD at 800 MHz displaying the three sets of mutual NOESY correlations pertaining to the three methyl groups located in rings C′–F′. NMR chemical shifts are calibrated relative to CHD_2OD (3.31 ppm). 48
Figure S63. ^{1}H NMR spectrum of MTX-6 acquired in CD_3OD at 800 MHz 48
Figure S64. COSY NMR spectrum of MTX-6 acquired in CD_3OD at 800 MHz........ 49
Figure S65. TOCSY NMR spectrum of MTX-6 acquired in CD_3OD at 800 MHz 49
Figure S66. HSQC NMR spectrum of MTX-6 acquired in CD_3OD at 800 MHz........ 50
Figure S67. Stacked ^{1}H NMR spectra (4.6–5.8 ppm) of (**A**) MTX-7 and (**B**) MTX-6 acquired in CD_3OD at 800 MHz showing the five sets of olefinic proton signals; H-2, 4=CH_2 (H-$144_{a,b}$), H-119 and H-120 (ring B'), H-141 and H-$142_{a,b}$........ 40
Figure S68. Expansion of the HSQC spectrum (^{1}H: 4.9–6.0 ppm; ^{13}C: 100–150 ppm) of MTX-6 acquired in CD_3OD at 800 MHz showing the five individual or pairs of olefinic protons, with assignments based on those defined for MTX-7 (Figure S52). NMR chemical shifts are calibrated relative to CHD_2OD (3.31 ppm) and CD_3OD (49.0 ppm)........ 51
Figure 69. Purification scheme of maitotoxin-6........ 52
Figure 70. Purification scheme of maitotoxin-7........ 53

Table S1. The ^1H and ^{13}C NMR chemical shifts (ppm), multiplicity and coupling constants (Hz) of the 22 methyl groups (numbering according to their chemical shift, starting upfield) observed in MTX-7. Spectra were acquired in CD_3OD using a 800 MHz spectrometer 35

Table S2. The ^1H and ^{13}C NMR chemical shifts (ppm; 800 MHz) of the five methine groups and the respective secondary methyl groups (the chemical shift corresponds to the underlined atom). Spectra were acquired in CD_3OD using a 800 MHz spectrometer 38

Table S3. The ^1H and ^{13}C NMR chemical shifts (ppm) of the aliphatic side chain atoms connected to ring A in MTX-7. Spectra were acquired in CD_3OD using a 800 MHz spectrometer........ 44

Table S4. The ^1H and ^{13}C NMR chemical shifts (ppm) of the aliphatic side chain atoms connected to ring F′ in MTX 7. Spectra were acquired in CD_3OD using a 800 MHz spectrometer........ 46

Table S5. The ^1H and ^{13}C NMR chemical shifts (ppm) and the HMBC ^{13}C correlations observed for the 22 methyl groups (numbered according to their chemical shift, starting upfield) in MTX-7. Spectra were acquired in CD_3OD using a 800 MHz spectrometer 47

Table S6. An inclusion list of the observed periodate oxidation fragment ions for MTX-1, MTX-6 and MTX-7 that were used for the CID experiments........ 54

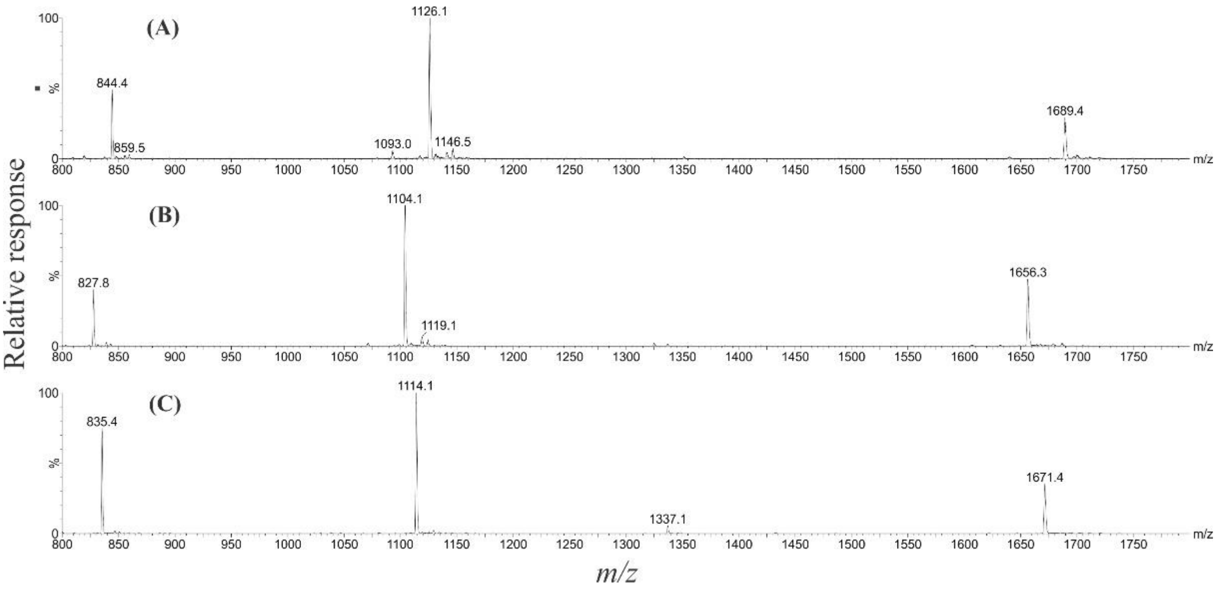

Figure S1. Full scan –ESI mass spectra of (**A**) MTX-1, (**B**) MTX-6 and (**C**) MTX-7 (*m/z* 800–1,800) showing the $[M–2H]^{2-}$ (*m/z* 1,689.4, 1,656.3 and 1,671.4), $[M–3H]^{3-}$ (*m/z* 1,126.1, 1,104.1 and 1,114.1) and $[M−4H]^{4-}$ (*m/z* 844.4, 827.8 and 835.4) ions, respectively.

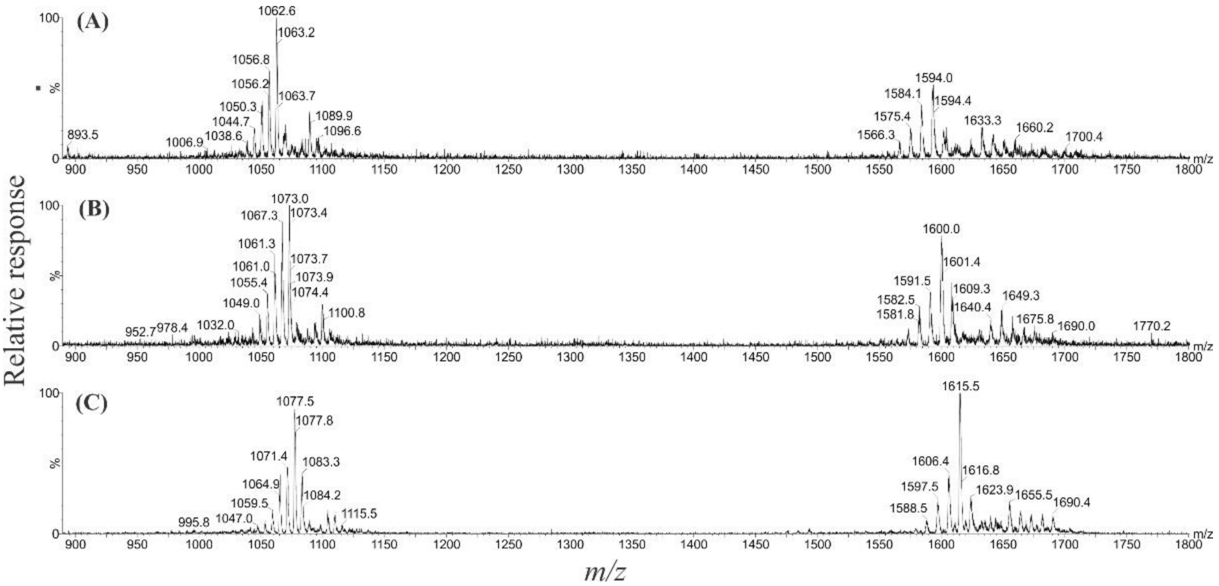

Figure S2. Full scan +ESI mass spectra of (**A**) MTX-1, (**B**) MTX-6 and (**C**) MTX-7 (*m/z* 800–1,800) showing the doubly-charged (*m/z* 1,594.0, 1,600.0 and 1,615.5) and triply-charged (*m/z* 1,062.8, 1,073.0 and 1,077.5) ions, respectively.

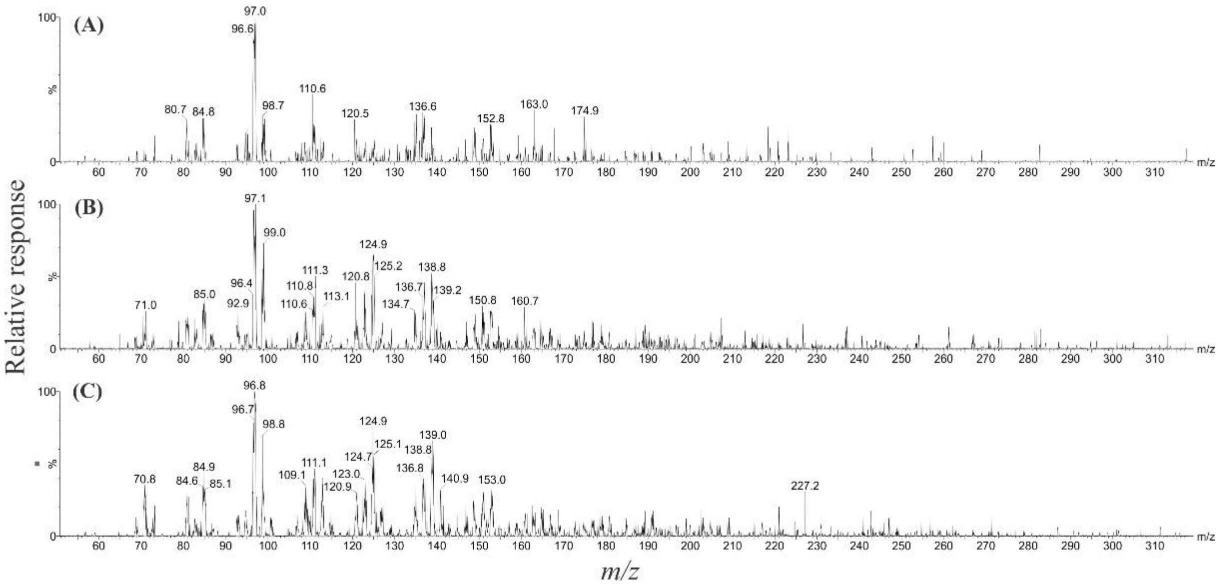

Figure S3. Lower mass range CID spectra of the dominant trianions $[M\text{-}3H]^{3-}$ of (**A**) MTX-1, (**B**) MTX-6 and (**C**) MTX-7 (60 eV CE; *m/z* 50−310).

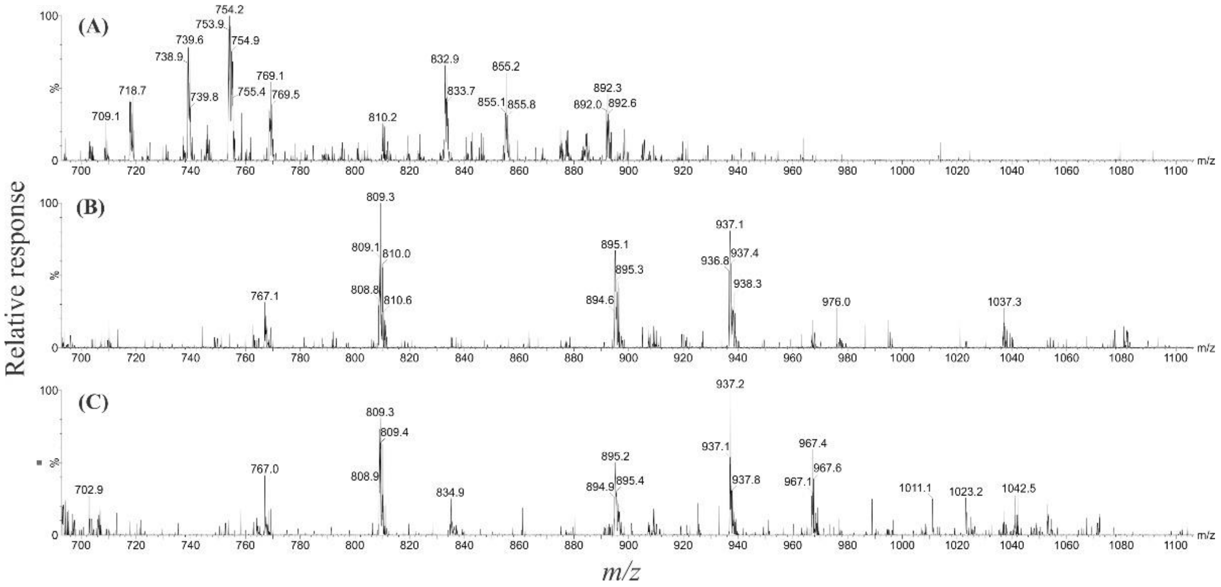

Figure S4. Higher mass range CID spectra of the dominant trianions $[M\text{-}3H]^{3-}$ of (**A**) MTX-1, (**B**) MTX-6 and (**C**) MTX-7 (60 eV CE; *m/z* 700−1,100).

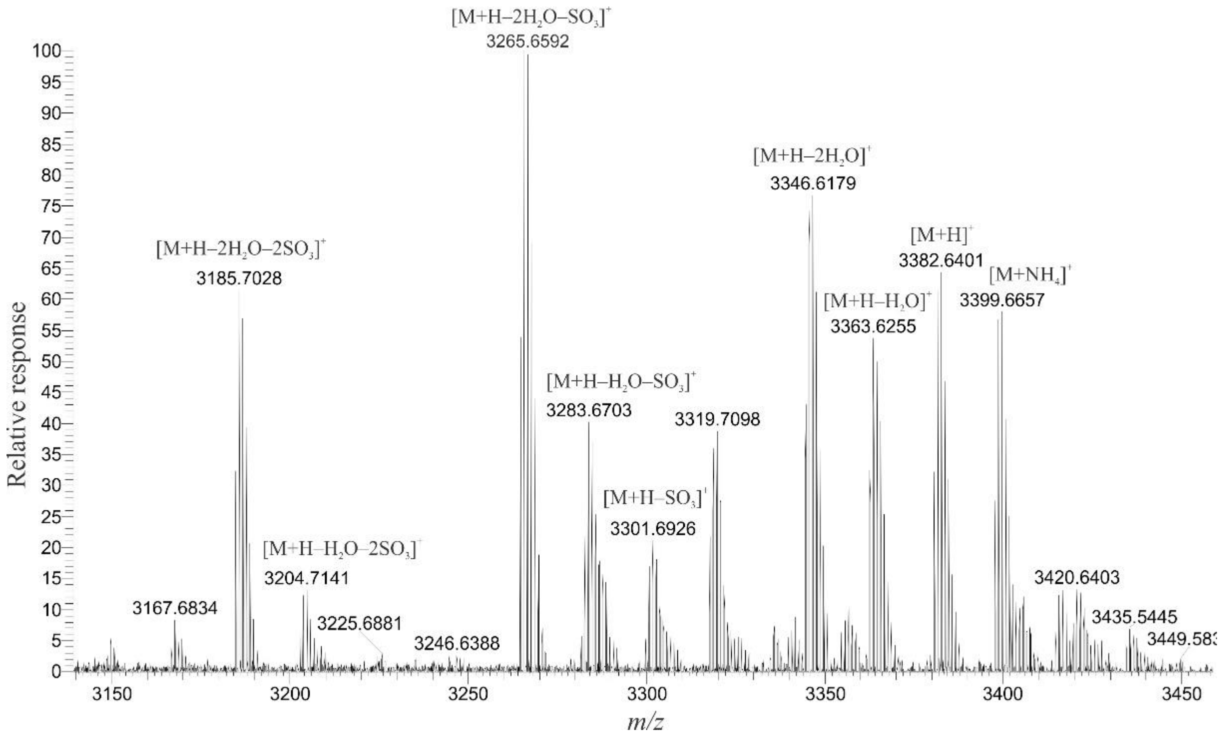

Figure S5. High resolution full scan mass spectrum of MTX-1 in +ESI mode showing the singly-charged cations (Δ +2.8 ppm). Note: this is outside the calibration range of the instrument.

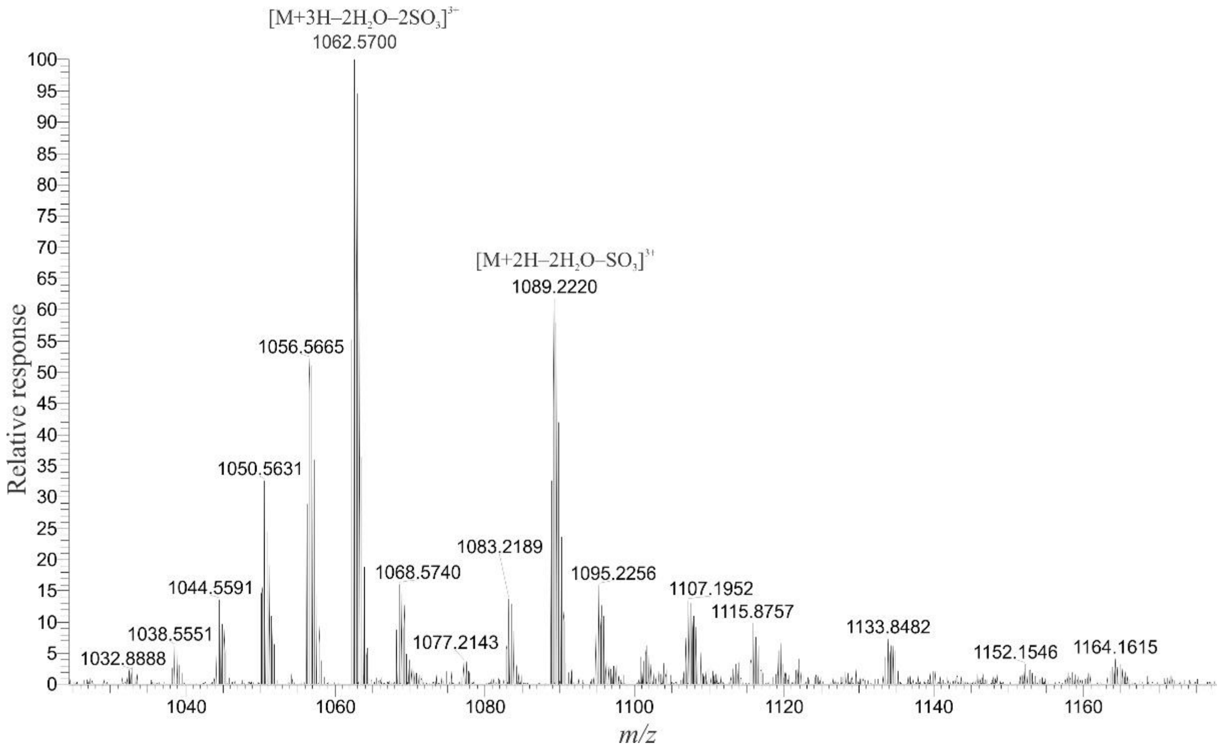

Figure S6. High resolution full scan mass spectrum of MTX-1 in +ESI mode showing the triply-charged cations (Δ –0.14 ppm).

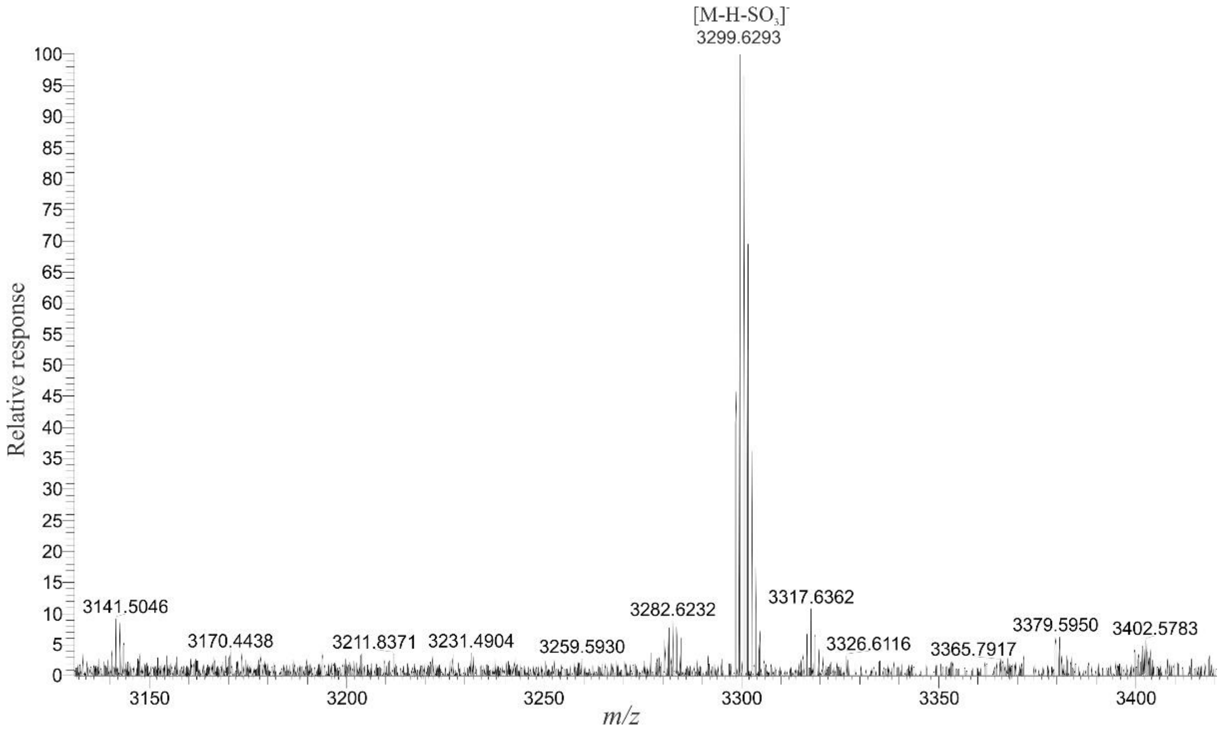

Figure S7. High resolution full scan mass spectrum of MTX-1 in –ESI mode showing a singly-charged anion (Δ –7.13 ppm). Note: this is outside the calibration range of the instrument.

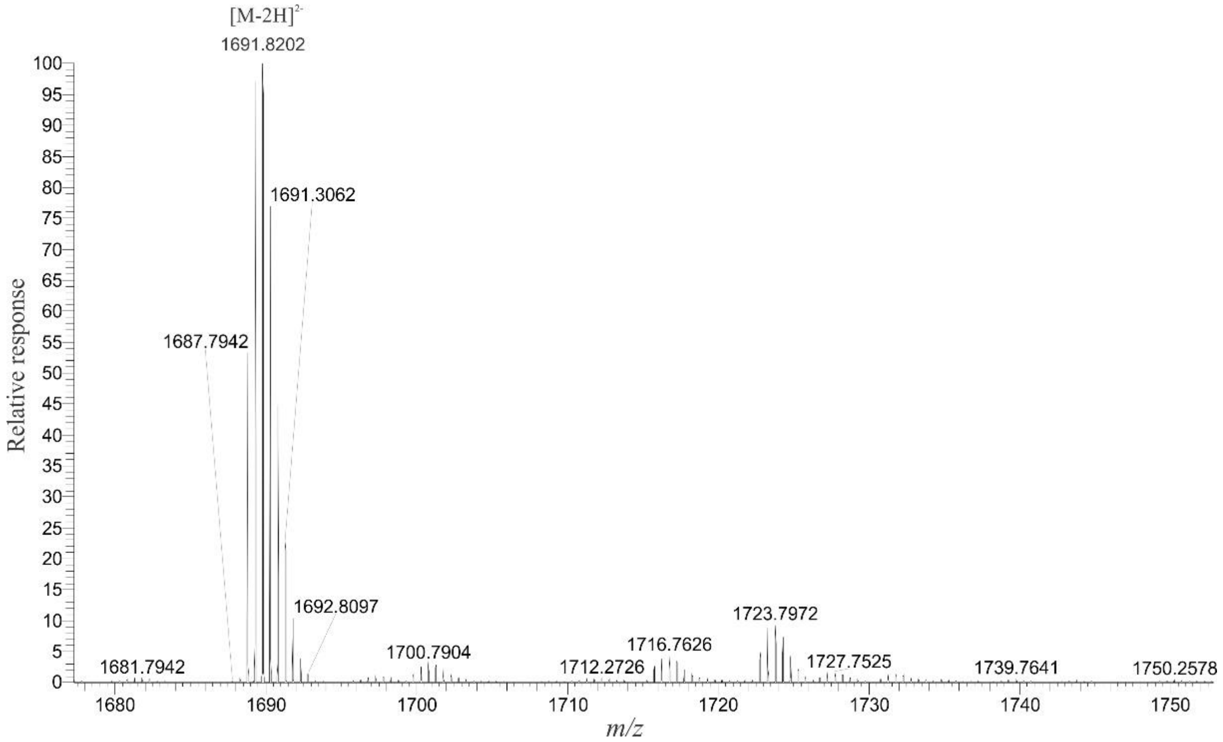

Figure S8. High resolution full scan mass spectrum of MTX-1 in –ESI mode showing a doubly-charged anion (Δ +1.49 ppm).

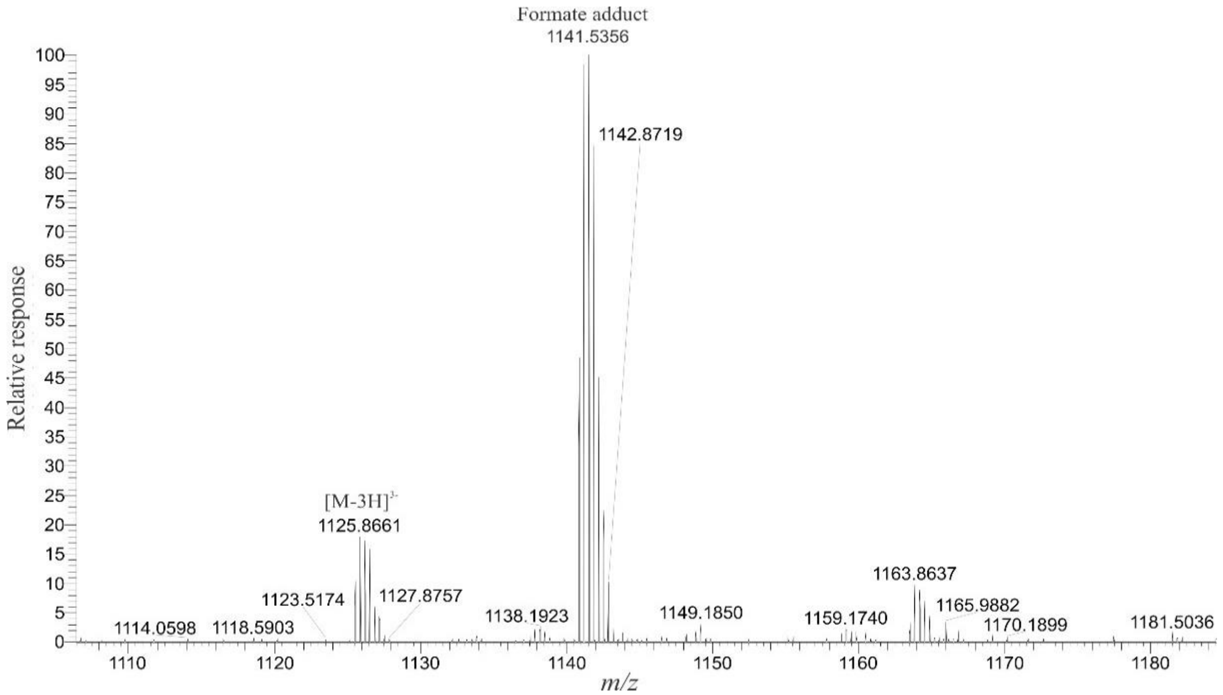

Figure S9. High resolution full scan mass spectrum of MTX-1 in –ESI mode showing the triply-charged anions (Δ –1.59 ppm).

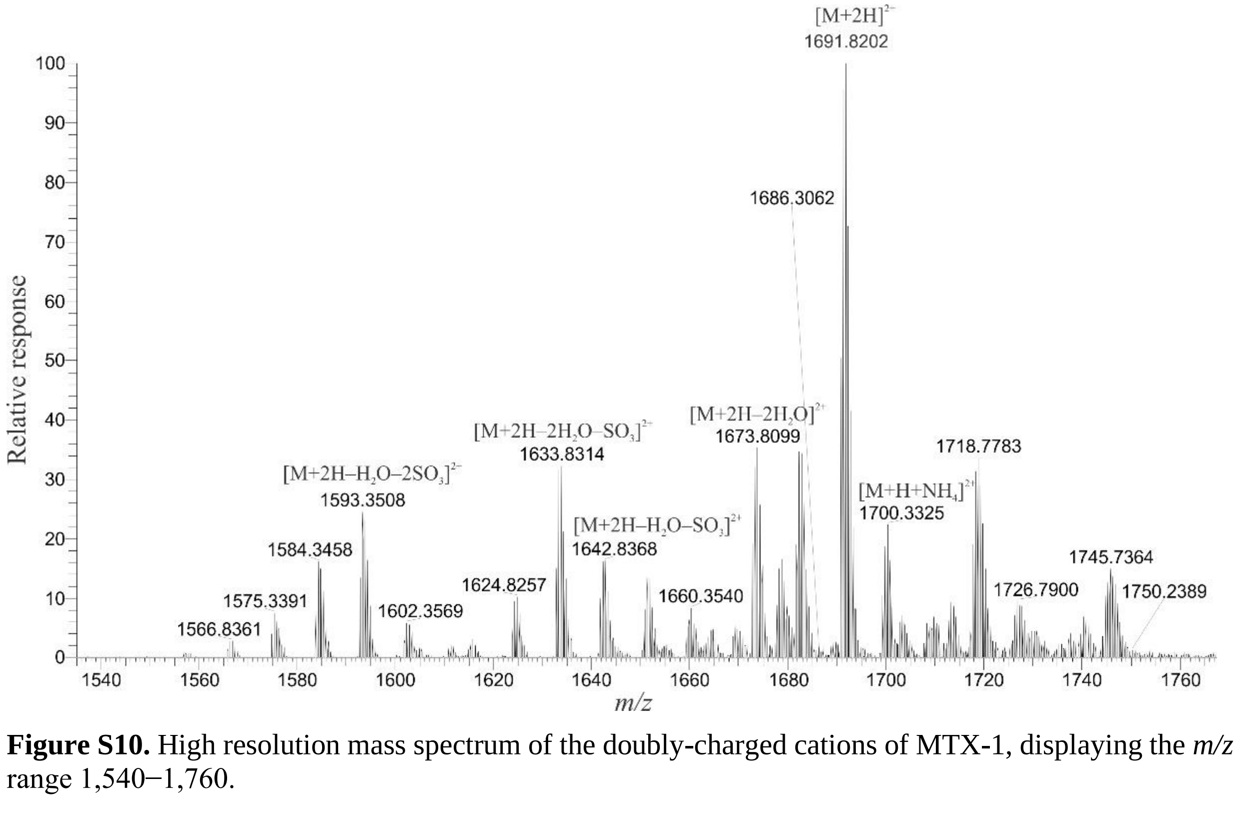

Figure S10. High resolution mass spectrum of the doubly-charged cations of MTX-1, displaying the *m/z* range 1,540−1,760.

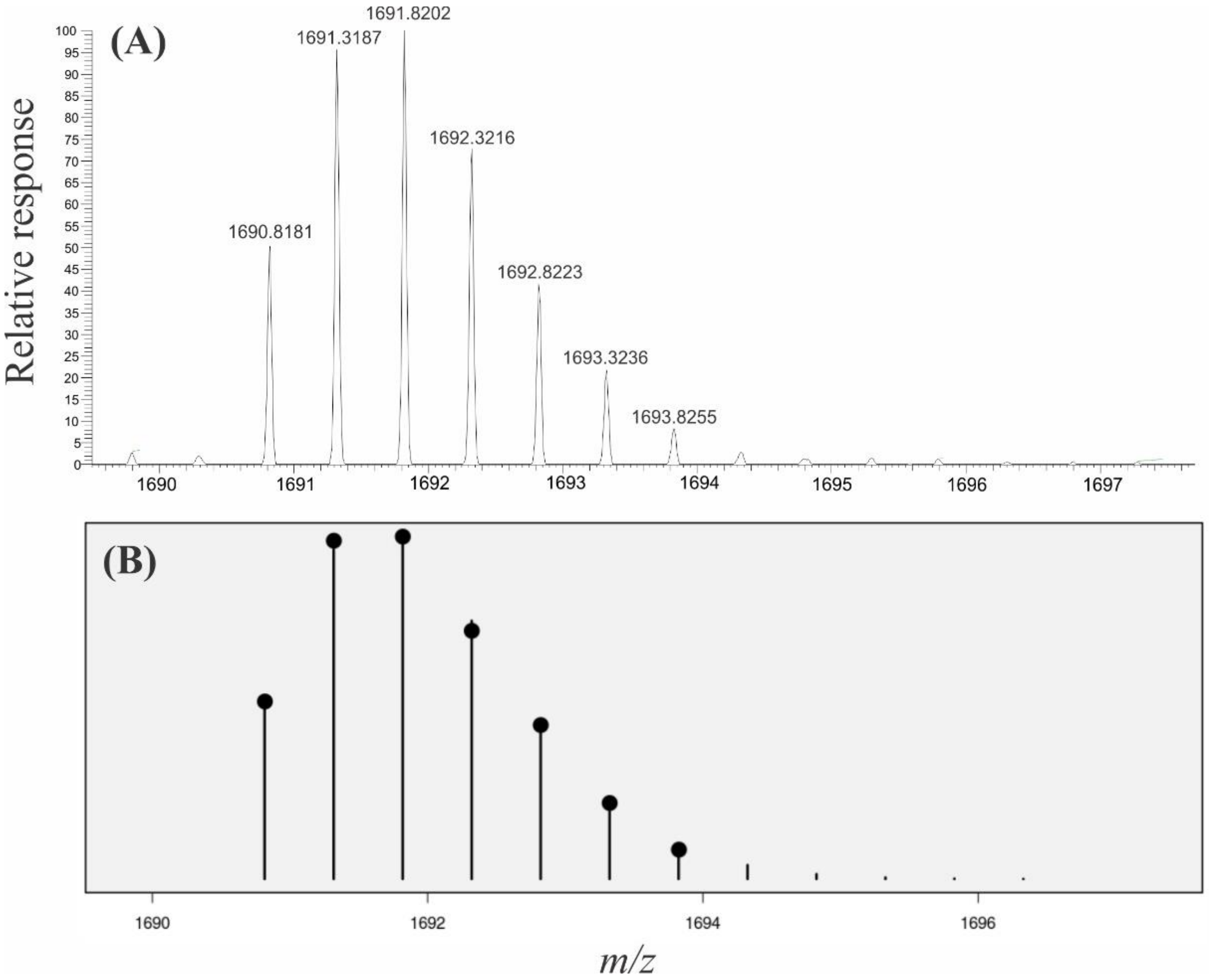

Figure S11. (**A**) Observed isotope distribution of the MTX-1 $[M+2H]^{2+}$ ion, compared to (**B**) the theoretical isotope distribution calculated using the NRCC Molecular Formula Calculator v1.01.[6]

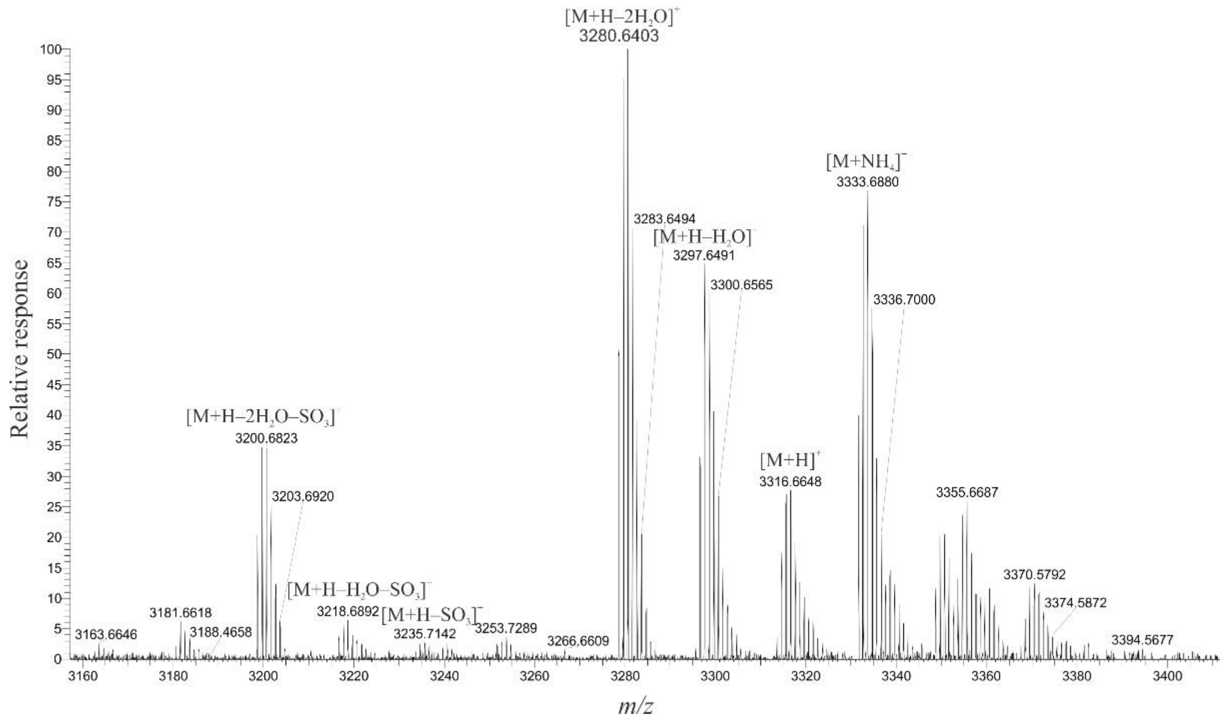

Figure S12. High resolution full scan mass spectrum of MTX-6 in +ESI mode showing the singly-charged cations. Note: this is outside the calibration range of the instrument.

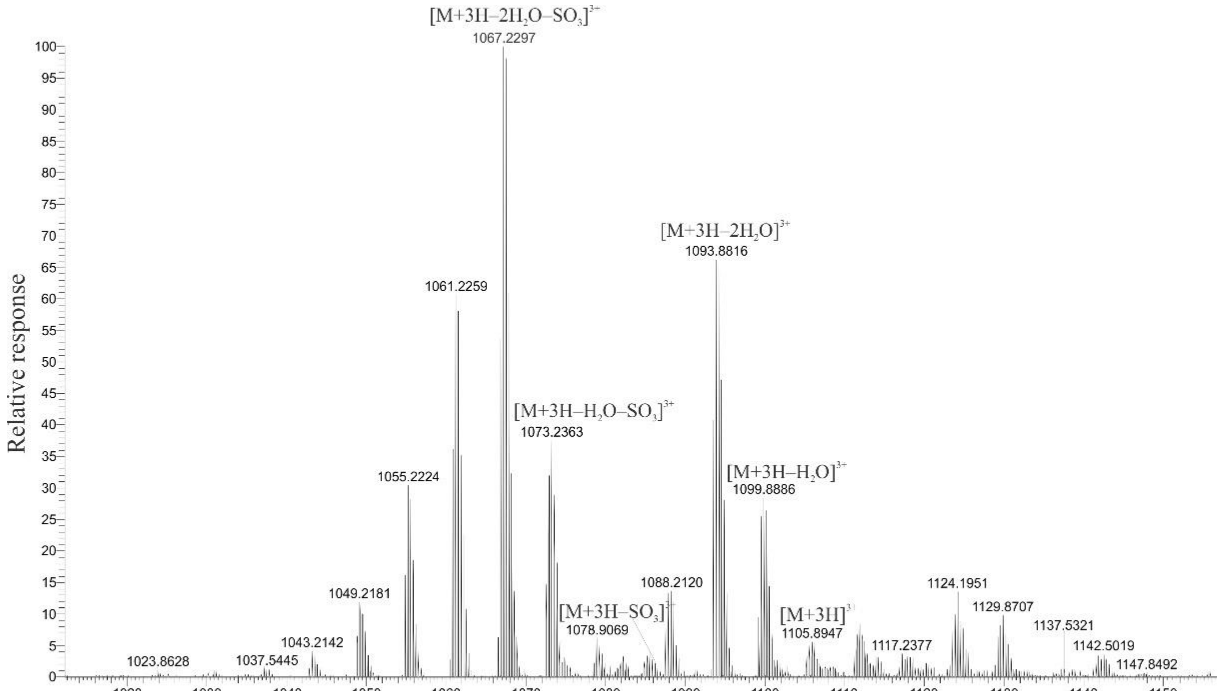

Figure S13. High resolution full scan mass spectrum of MTX-6 in +ESI mode showing the triply-charged cations.

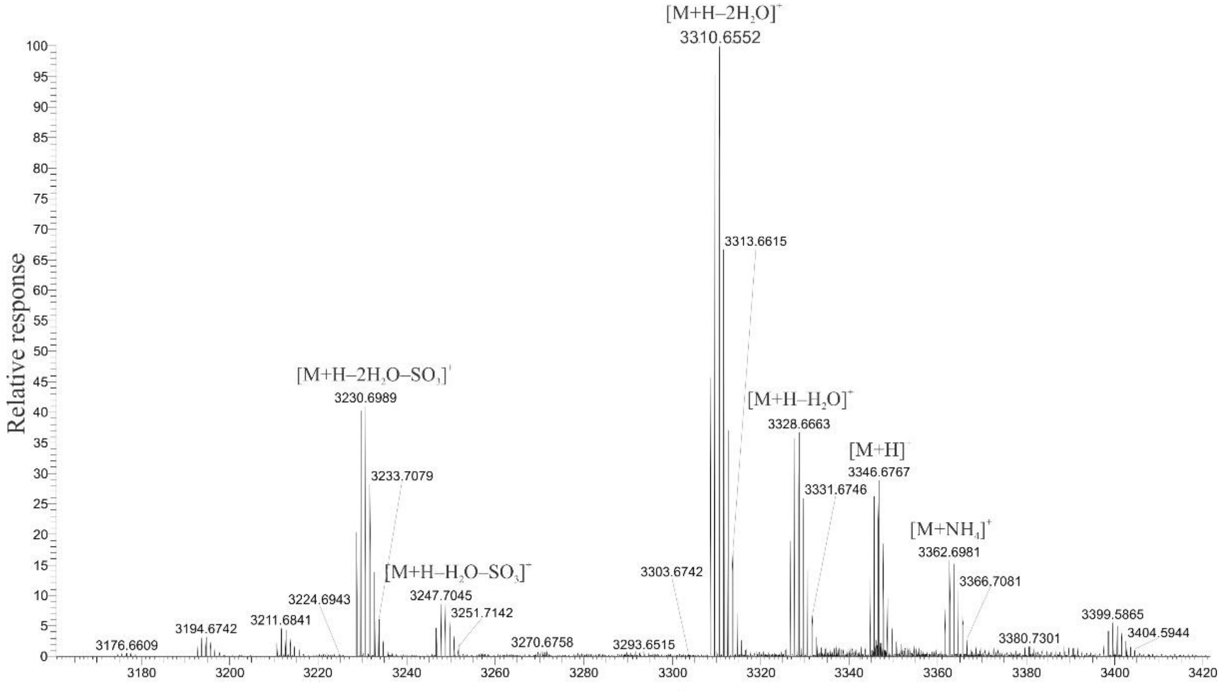

Figure S14. High resolution full scan mass spectrum of MTX-7 in +ESI mode showing the singly-charged cations. Note: this is outside the calibration range of the instrument.

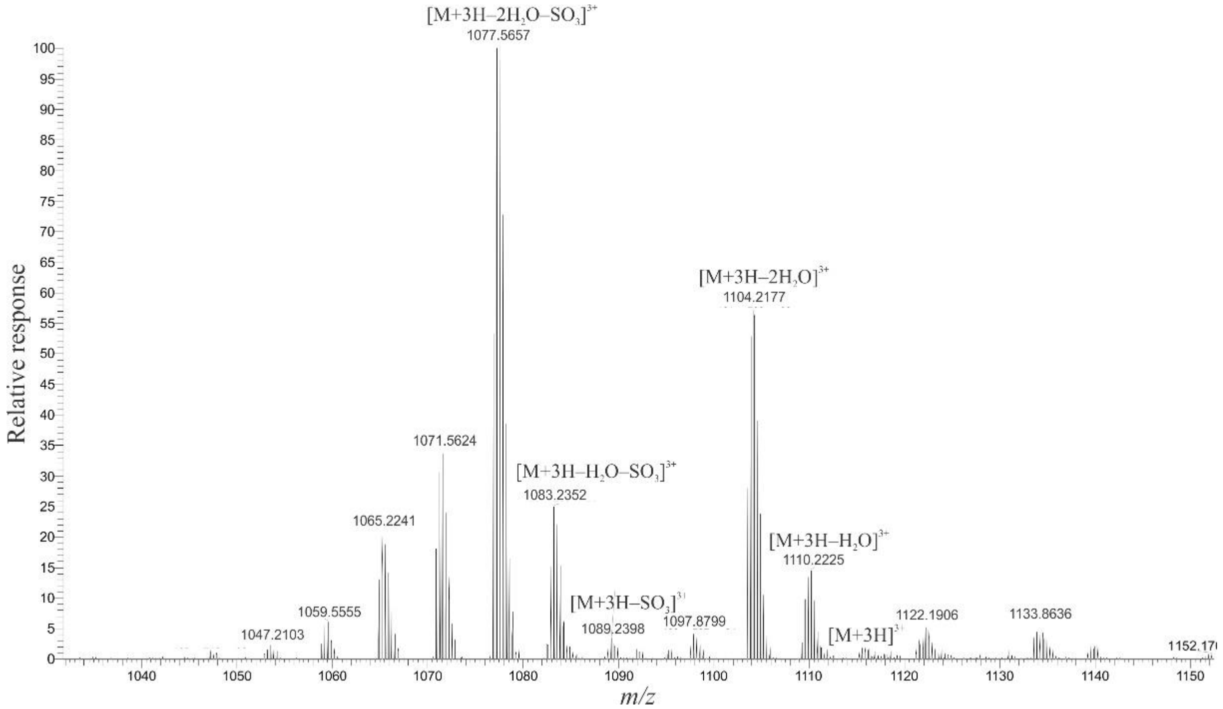

Figure S15. High resolution full scan mass spectrum of MTX-7 in +ESI mode showing the triply-charged cations.

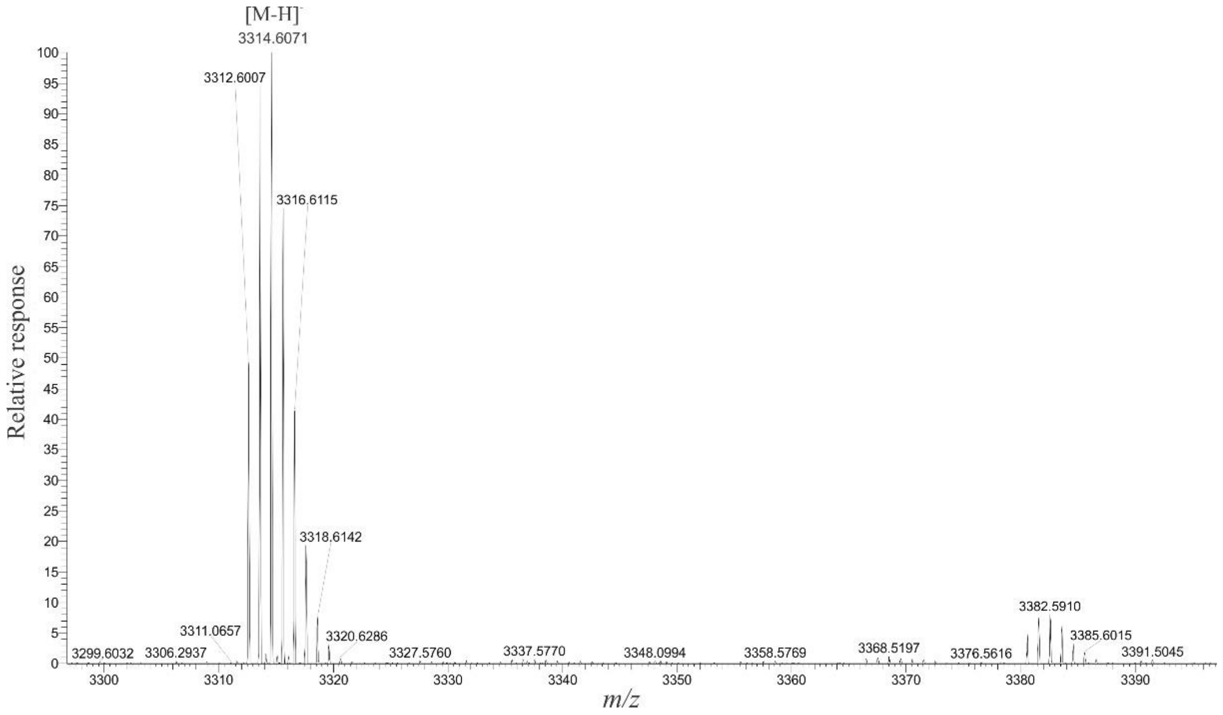

Figure S16. High resolution full scan mass spectrum of MTX-6 in –ESI mode showing a singly-charged anion. Note: this is outside the calibration range of the instrument.

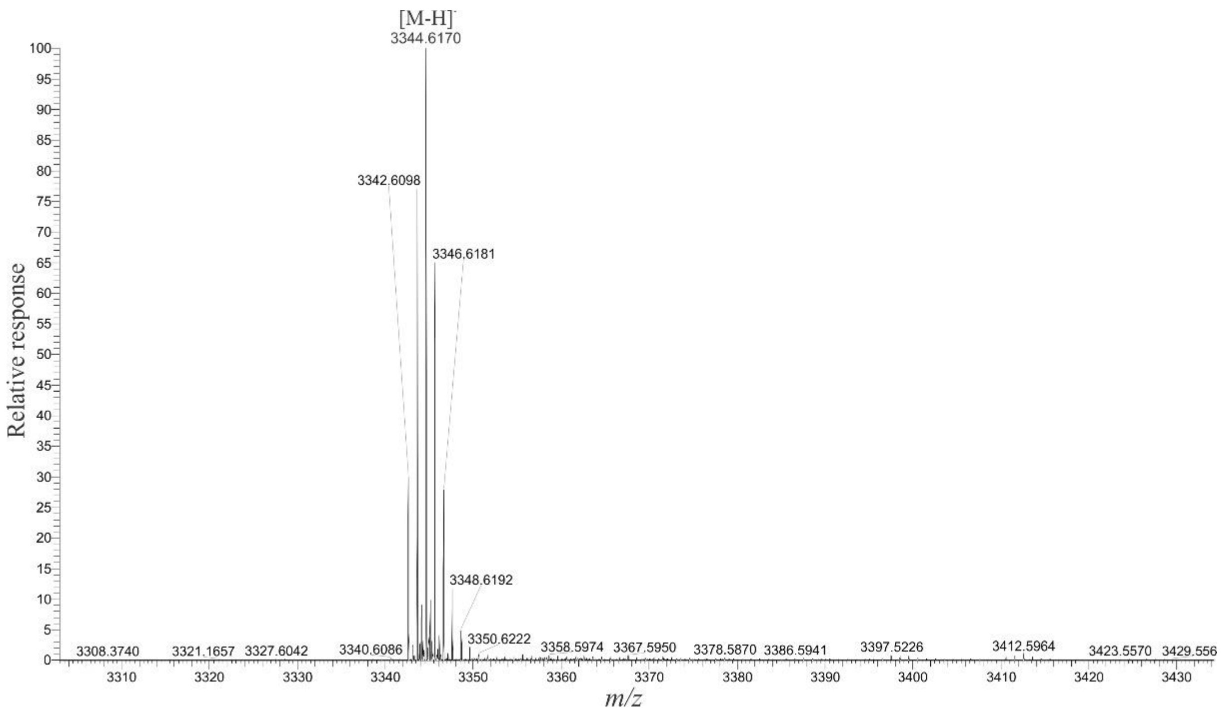

Figure S17. High resolution full scan mass spectrum of MTX-7 in –ESI mode showing a singly-charged anion. Note: this is outside the calibration range of the instrument.

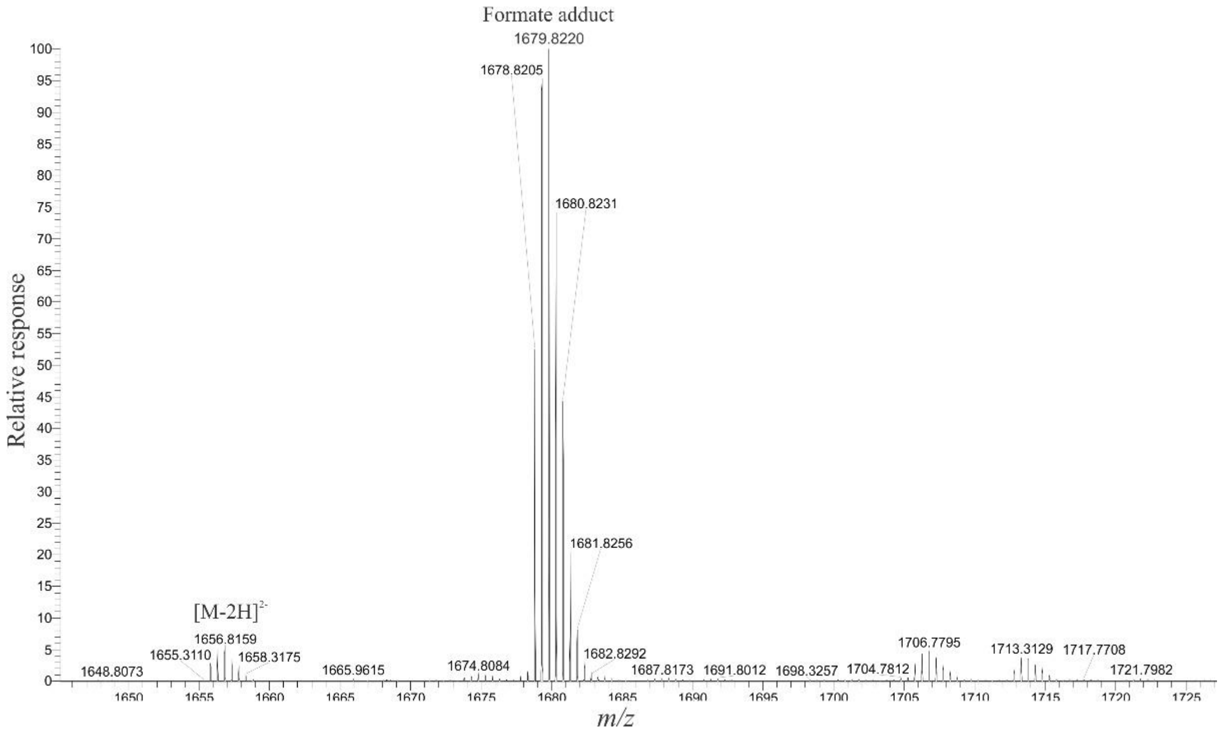

Figure S18. High resolution full scan mass spectrum of MTX-6 in –ESI mode showing the doubly-charged anions.

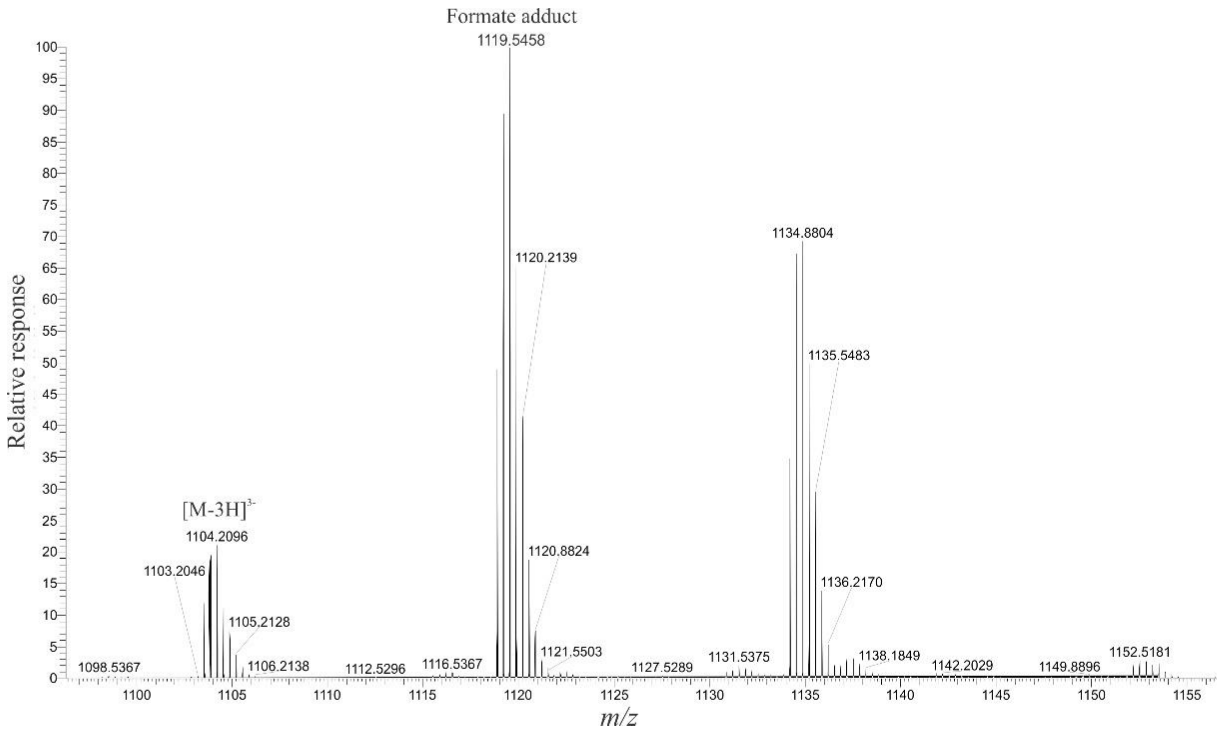

Figure S19. High resolution full scan mass spectrum of MTX-6 in –ESI mode showing the triply-charged anions.

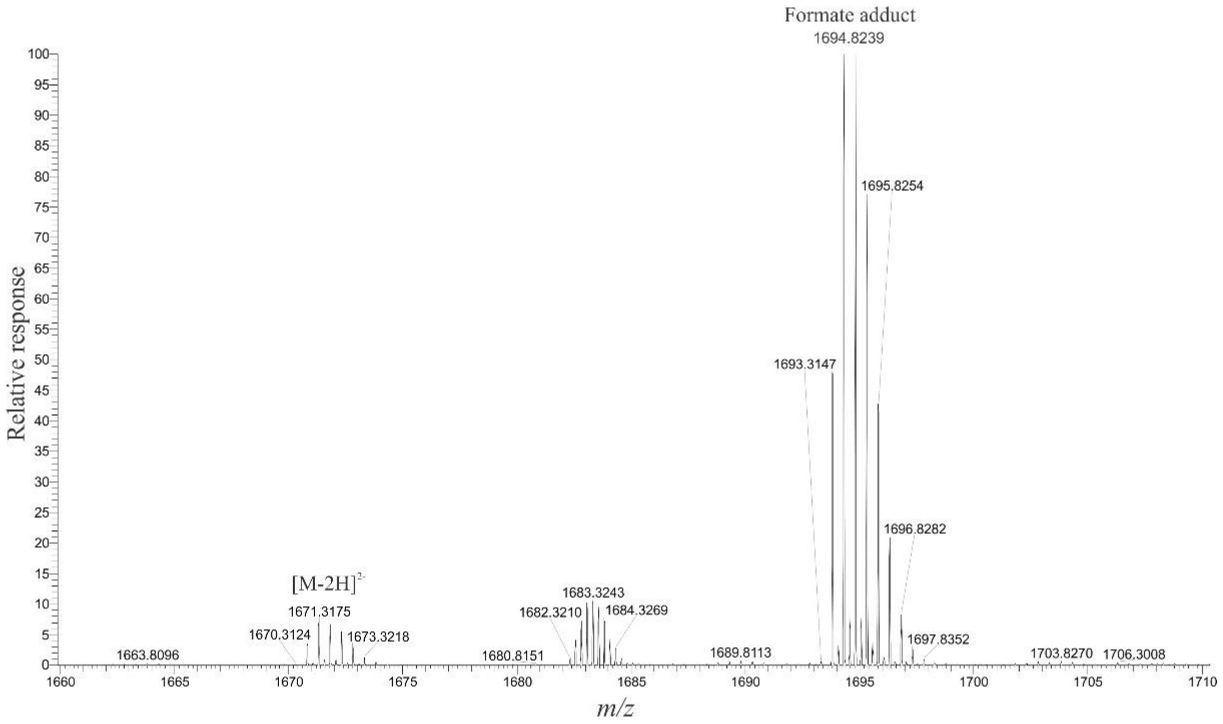

Figure S20. High resolution full scan mass spectrum of MTX-7 in –ESI mode showing the doubly-charged anions.

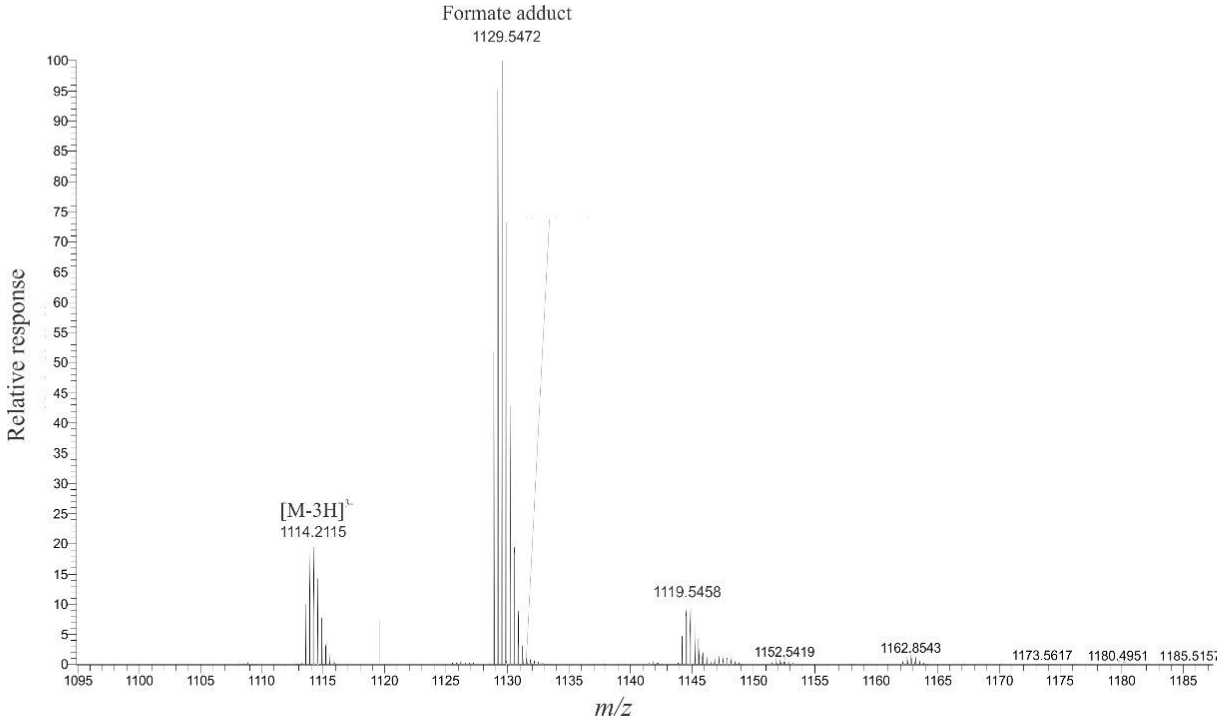

Figure S21. High resolution full scan mass spectrum of MTX-7 in –ESI mode showing the triply-charged anions.

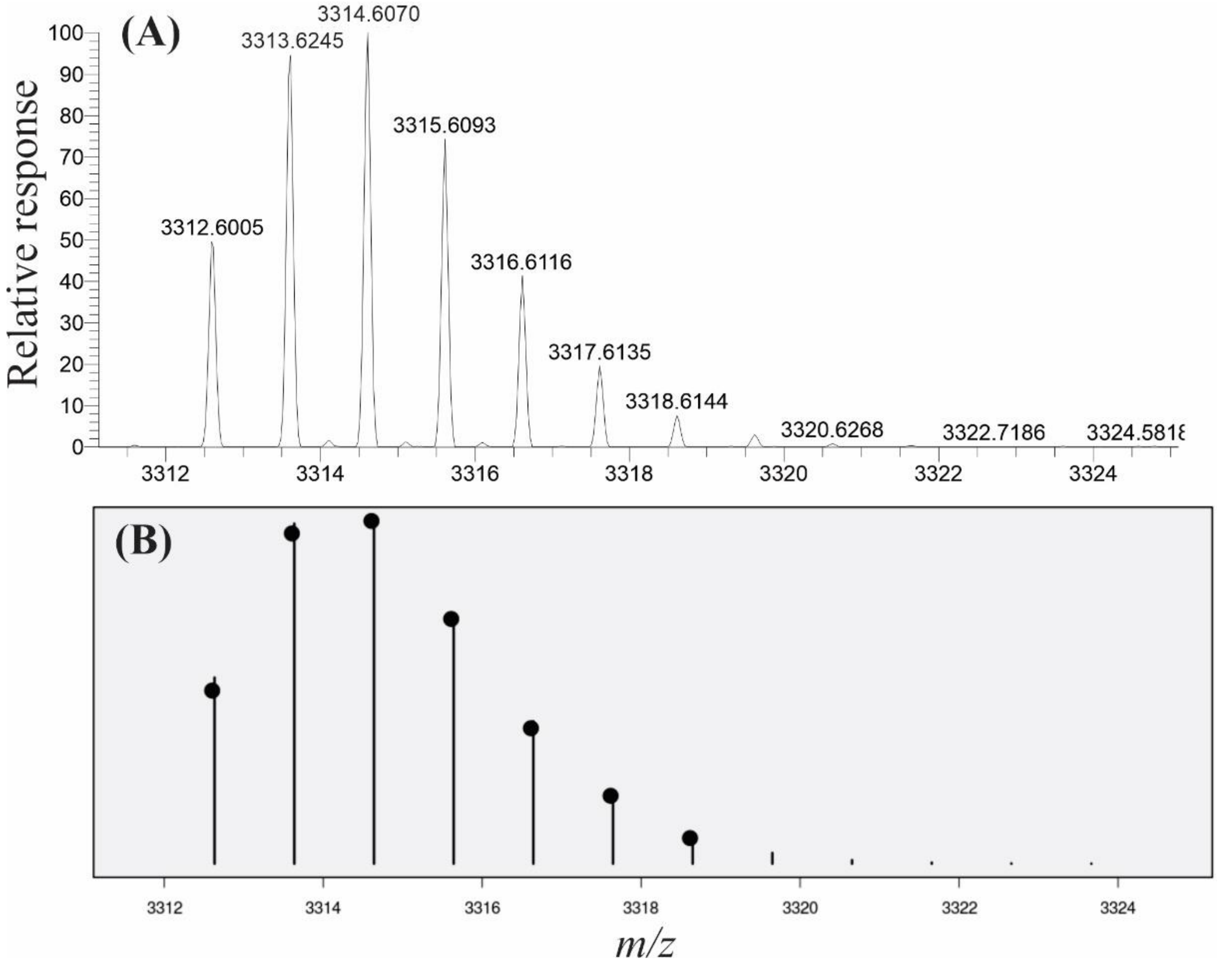

Figure S22. (**A**) Observed isotope distribution of the MTX-6 cation $[M+H]^+$, compared to (**B**) the theoretical isotope distribution calculated using the NRCC Molecular Formula Calculator v1.01.[6]

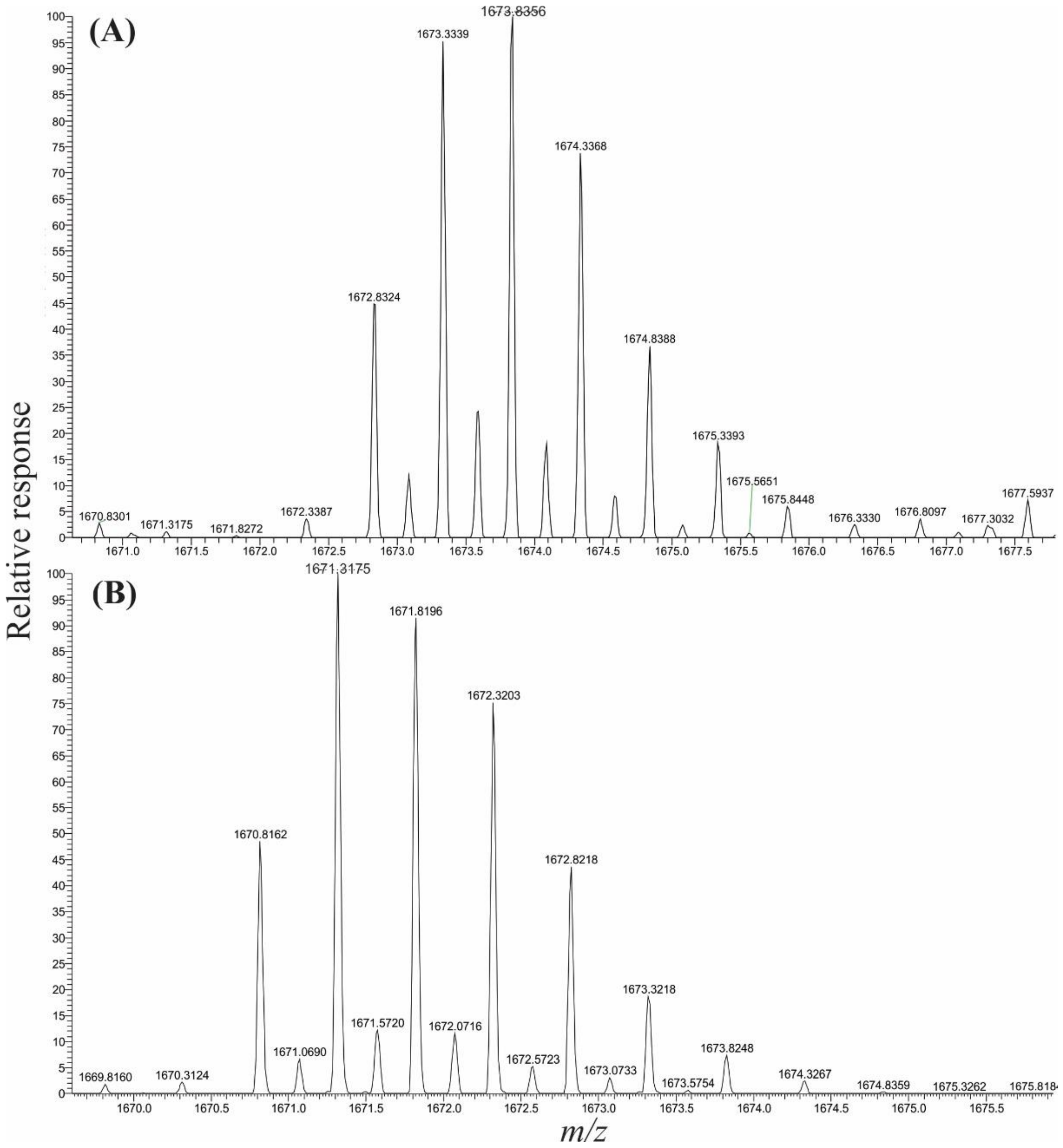

Figure S23. Expanded view of (**A**) $[M+2H]^{2+}$ and (**B**) $[M–2H]^{2-}$, of MTX-7, displaying the signals from the single (most intense) and double (minor) molecular ions.

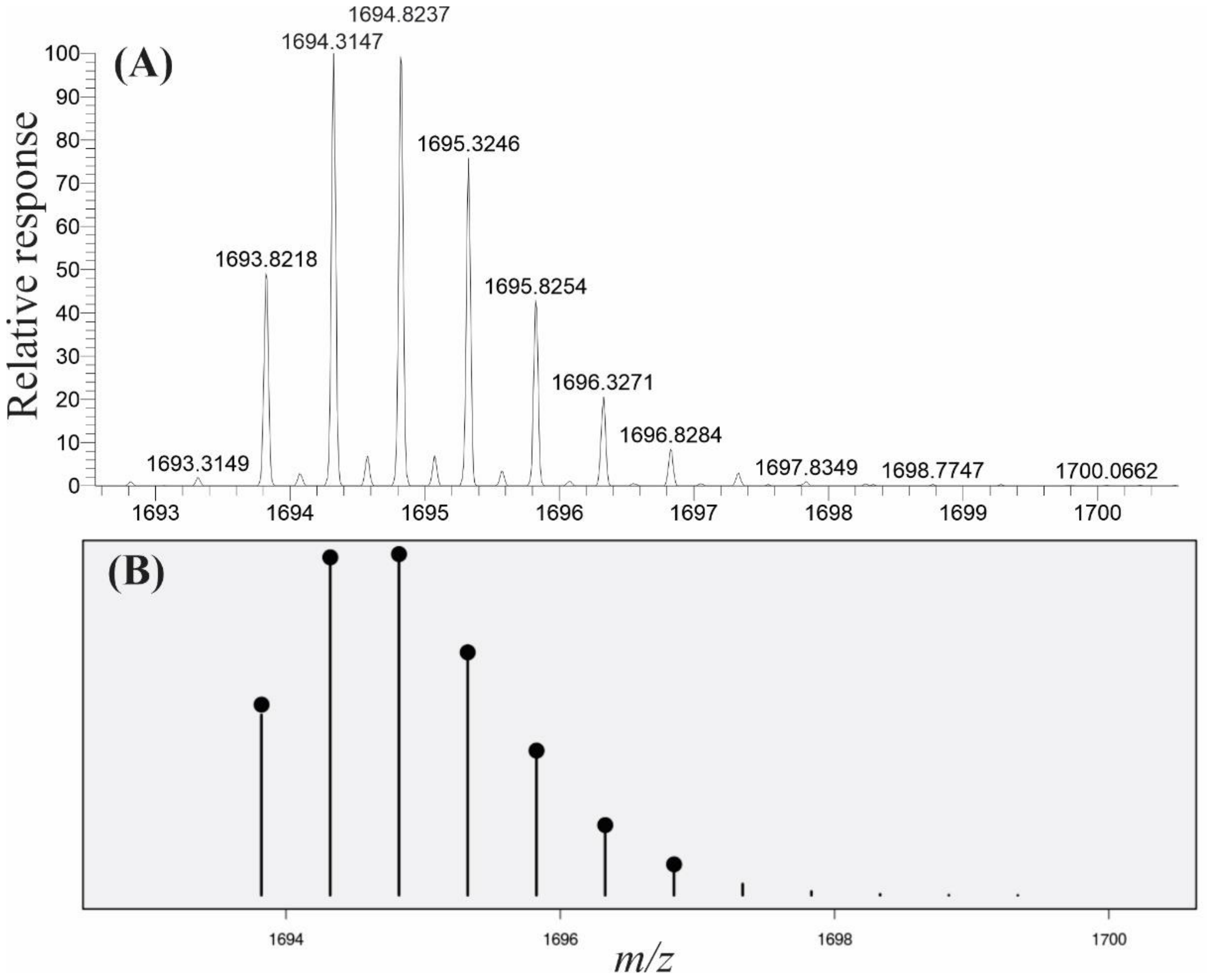

Figure S24. (**A**) Observed isotope distribution of the MTX-7 doubly-charged formate adduct, compared to (**B**) the theoretical isotope distribution calculated using the NRCC Molecular Formula Calculator v1.01.[6]

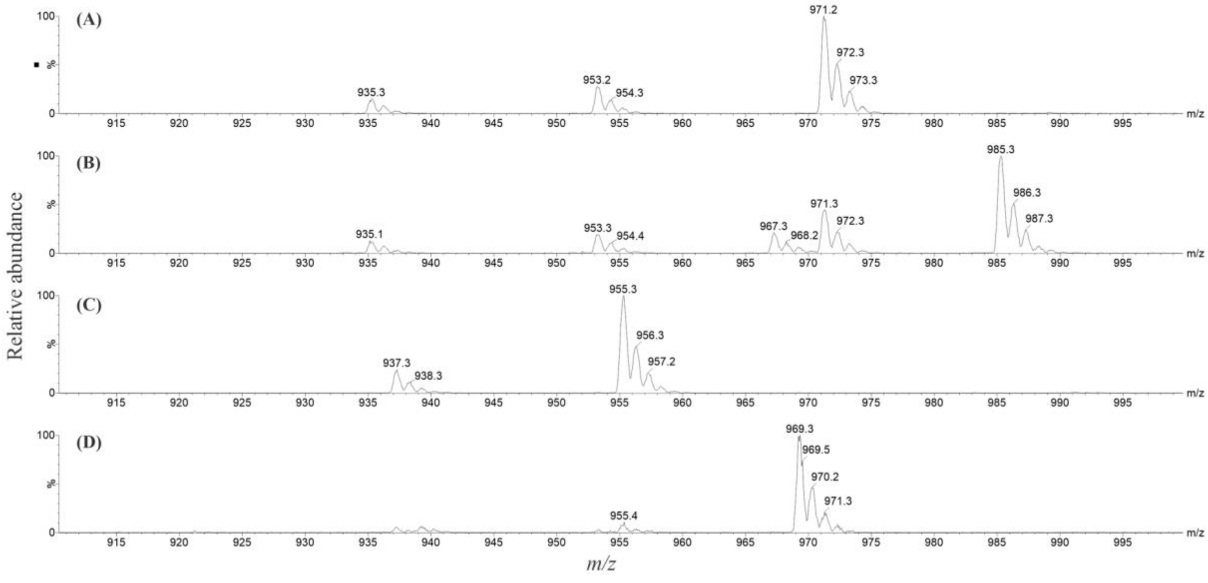

Figure S25. Full scan –ESI mass spectra of the oxidative cleavage products of the (**A**) peak at 1.31 min and (**B**) the peak at 1.35 min observed in MTX-1 and MTX-7. Also included are the peaks observed at (**C**) 1.36 min and (**D**) 1.39 min in MTX-6.

(**A**) $[M–H]^-$ = 971.3

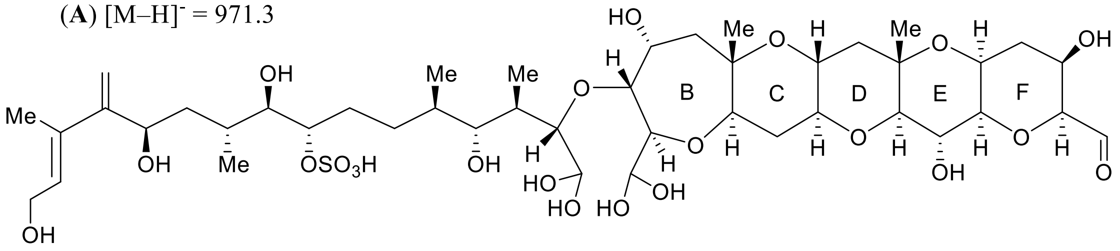

(**B**) $[M–H]^-$ = 953.3

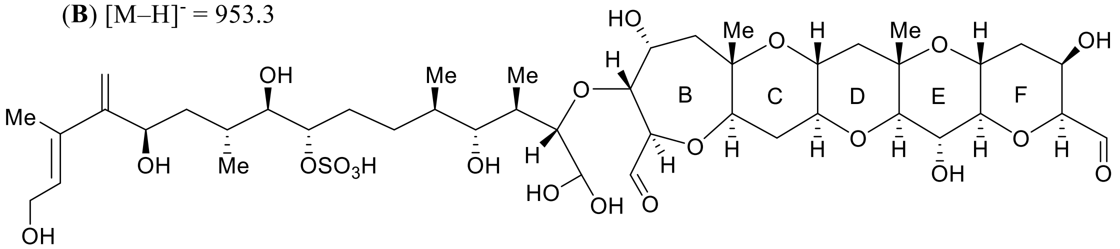

(**C**) $[M–H]^-$ = 935.3

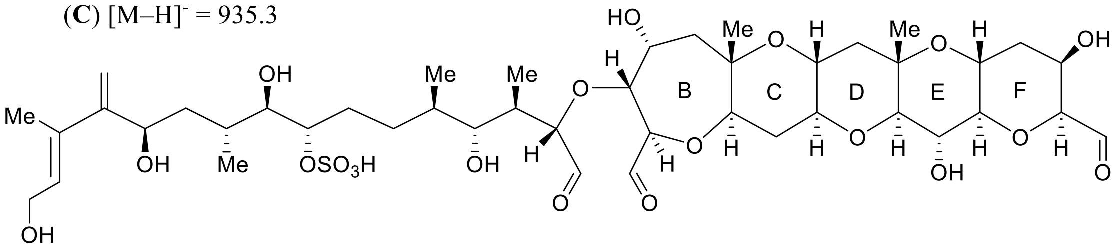

(**D**) $[M–H]^-$ = 985.3

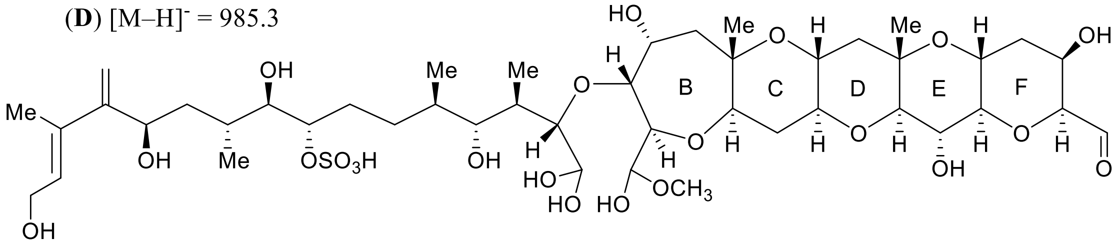

Figure S26. Proposed structures of the products of periodate oxidation of MTX-1 and MTX-7 pertaining to the common ions observed in the spectra of the compounds.

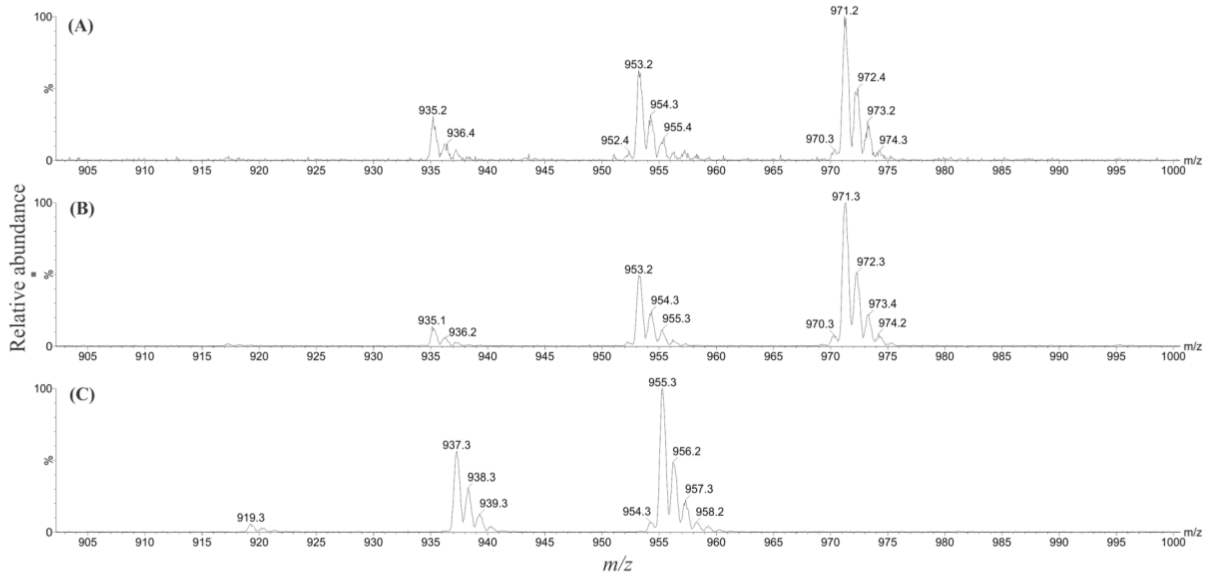

Figure S27. Full scan –ESI mass spectra of the oxidative cleavage products of the (**A**) peak at 1.37 min and (**B**) peak at 1.39 min observed in MTX-1 and MTX-7. Also included is the peak observed at (**C**) 1.42 min in MTX-6 (acquired using a BEH phenyl column and ammoniated mobile phases).

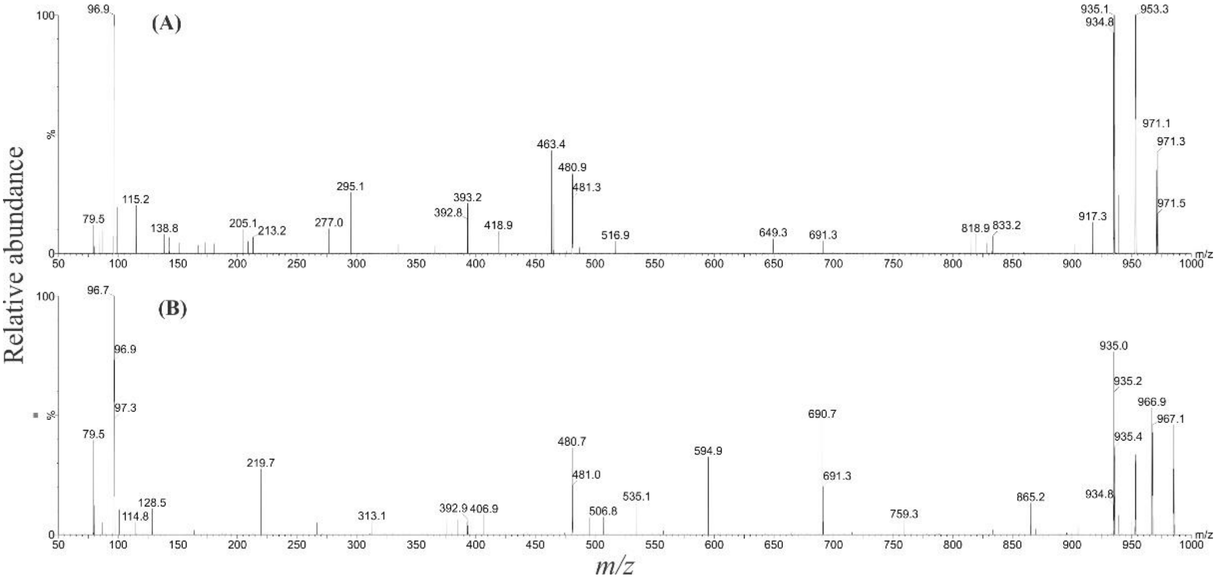

Figure S28. CID of the dominant oxidation cleavage products (**A**) *m/z* 971.2 at 1.31 min and (**B**) *m/z* 985.3 at 1.35 min for MTX-1 using 60 eV CE.

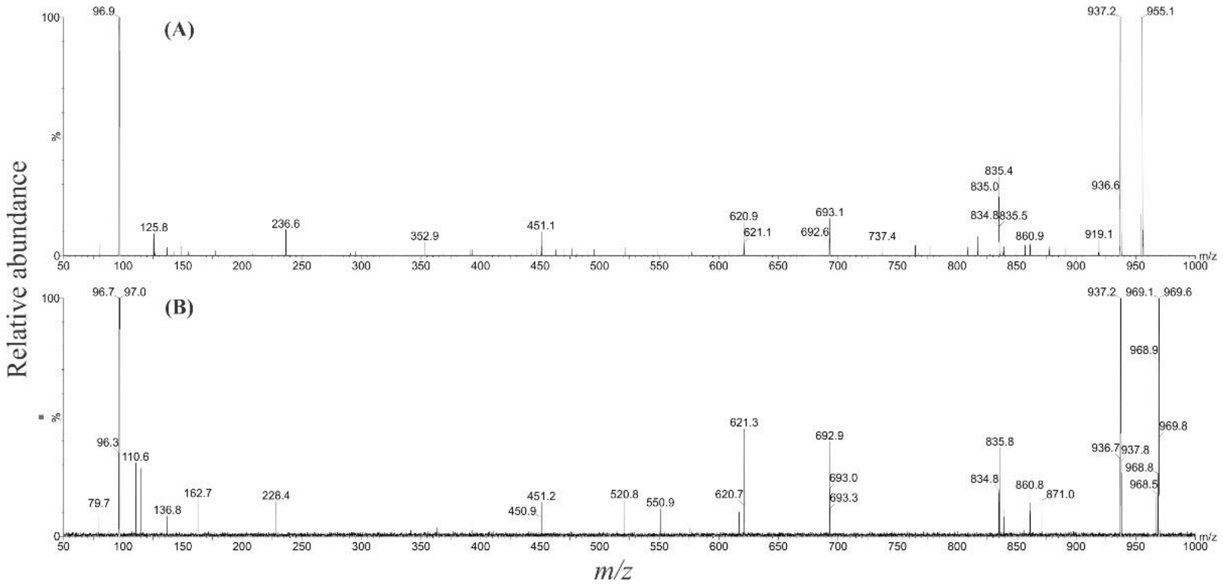

Figure S29. CID of the dominant oxidation cleavage products (**A**) *m/z* 955.3 at 1.36 min and (**B**) *m/z* 969.3 at 1.39 min for MTX-6 using 60 eV CE.

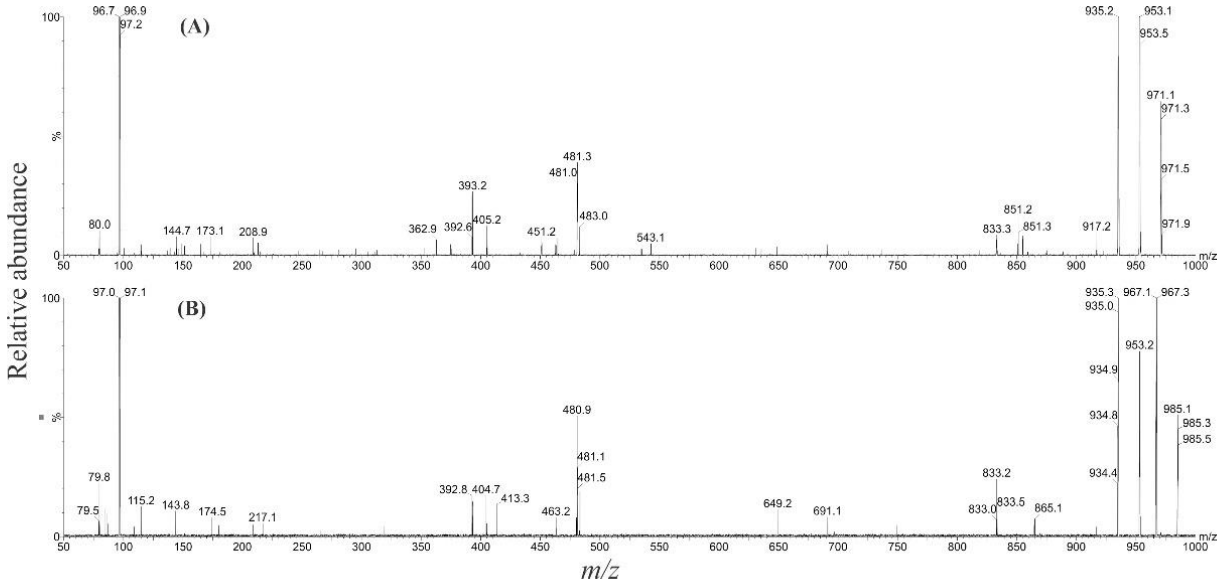

Figure S30. CID of the dominant oxidation cleavage products (**A**) *m/z* 971.2 at 1.31 min and (**B**) *m/z* 985.3 at 1.35 min for MTX-7 using 60 eV CE.

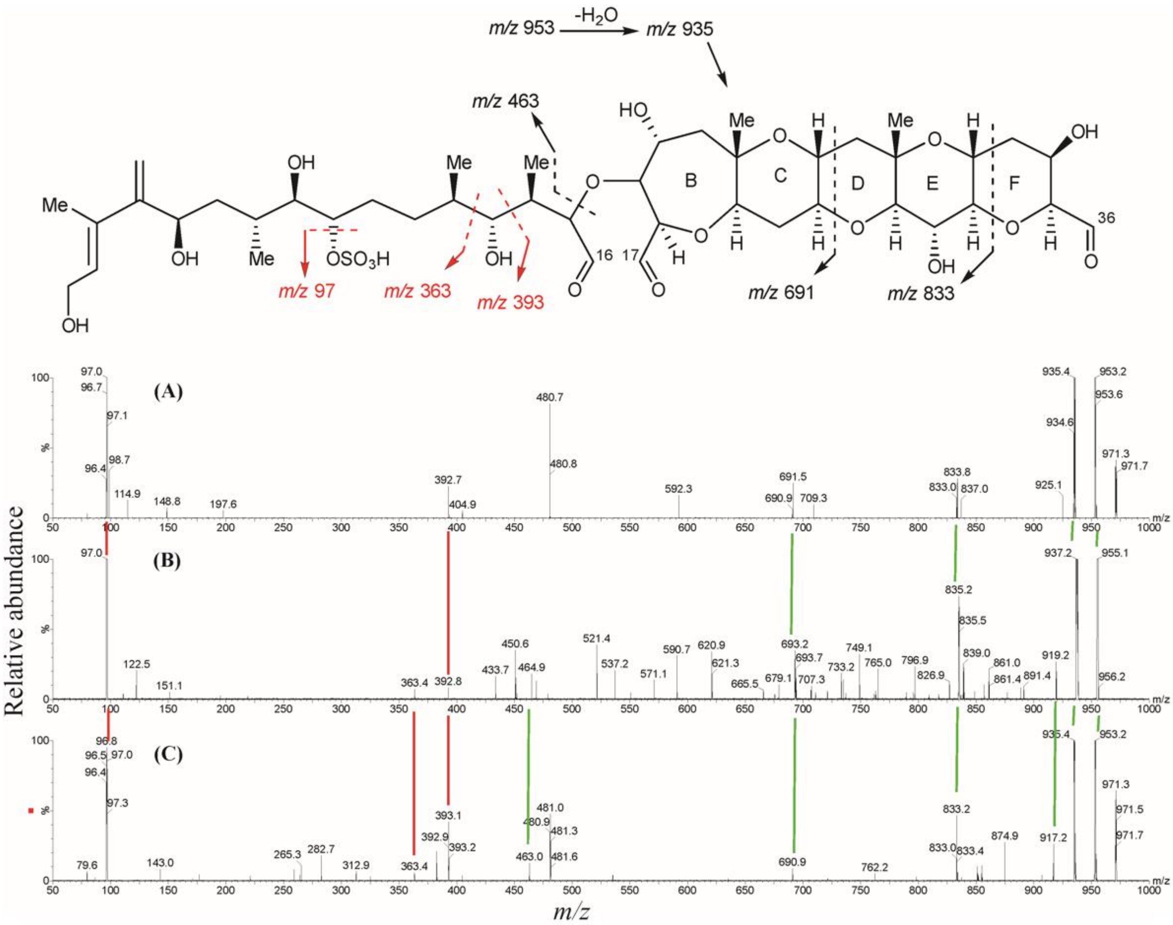

Figure S31. The proposed CID fragmentation of Fragment A in –ESI (60 eV CE; *m/z* 50−1,000) and comparison of the corresponding spectra of (**A**) MTX-1, (**B**) MTX-6 and (**C**) MTX-7. Green lines indicate the fragments that are 2 Da higher in mass for MTX-6 compared to MTX-1 and MTX-7. Red lines indicate the fragments that align between the three MTX analogues.

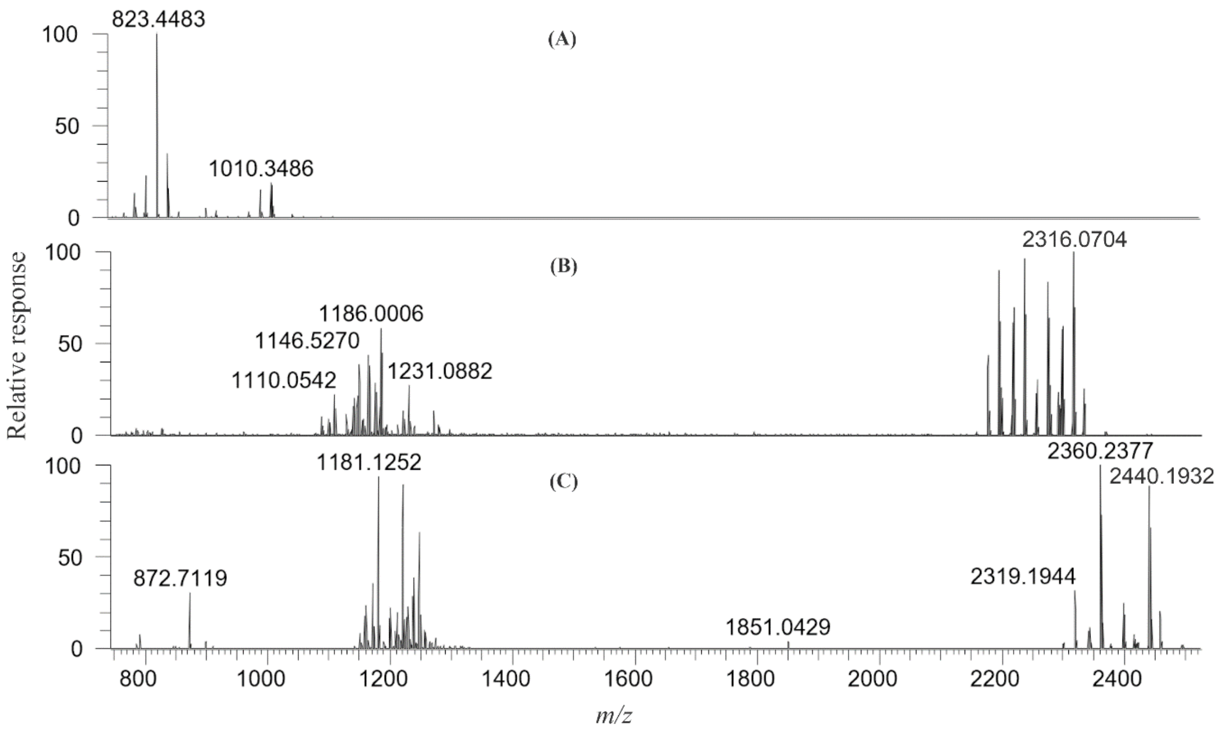

Figure S32. Full scan +ESI HR mass spectra (*m/z* 800–2,500) of the oxidative cleavage products of MTX-1 displaying (**A**) Fragment A, (**B**) Fragment B and (**C**) Fragments B and C connected.

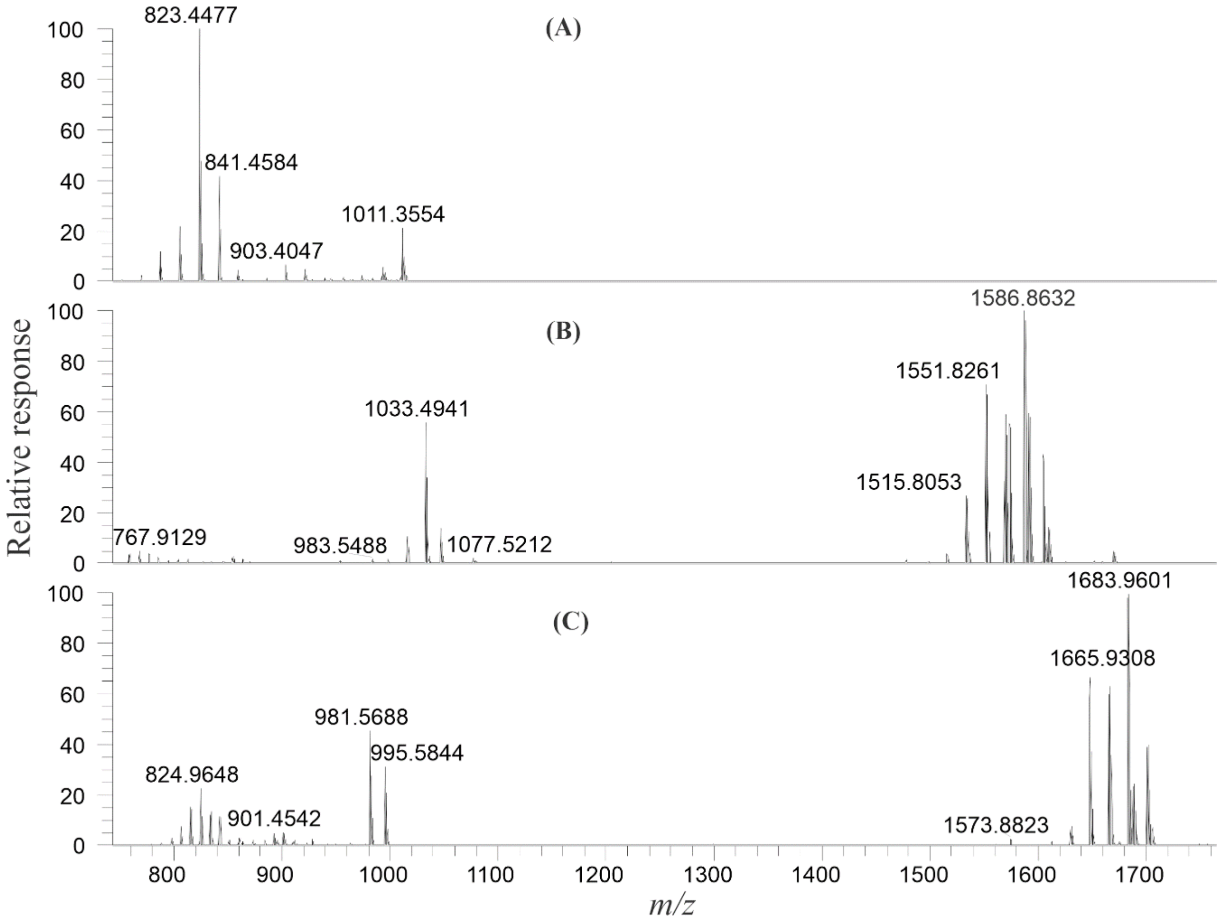

Figure S33. Full scan +ESI HR mass spectra (*m/z* 800–2,500) of the oxidative cleavage products of MTX-6 displaying (**A**) ‘Fragment A’, (**B**) ‘Fragment B’ and (**C**) ‘Fragments B and C’ connected.

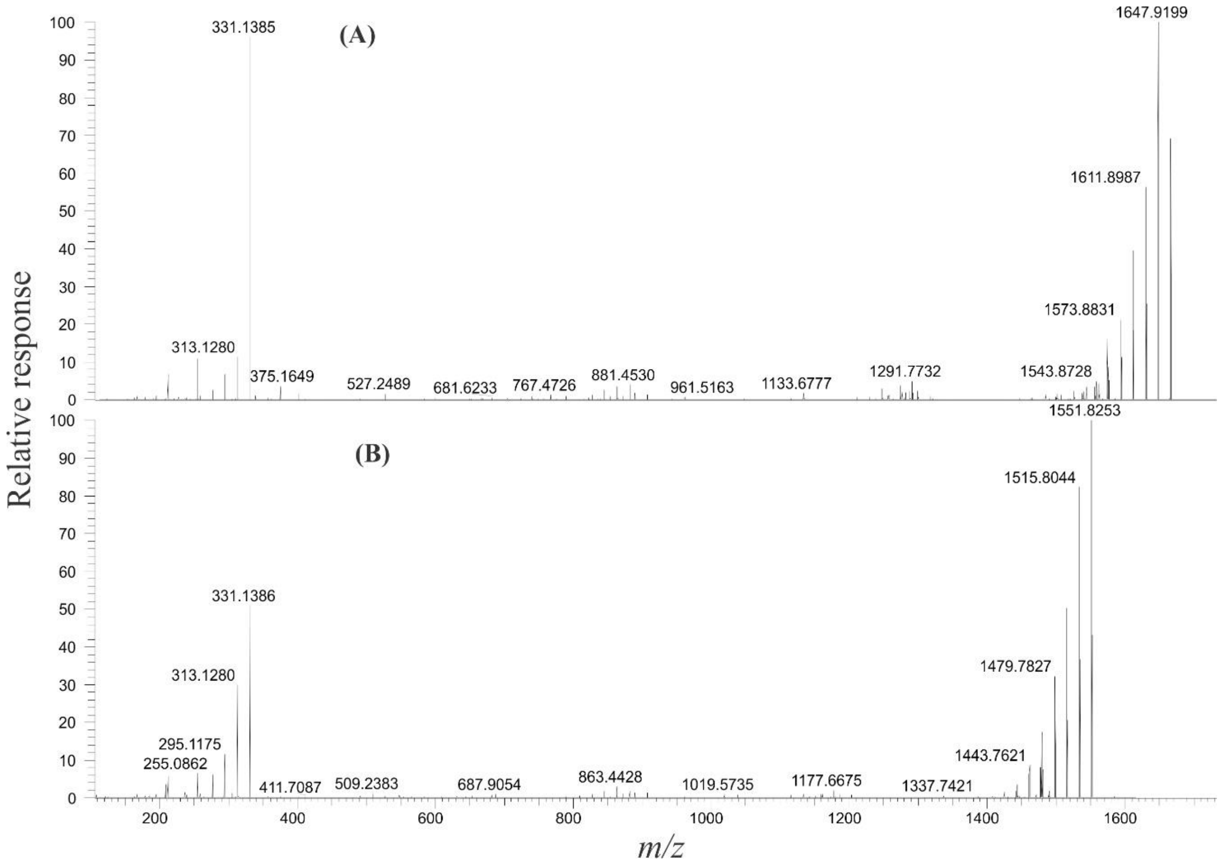

Figure S34. CID fragmentation spectra of the two unknown ions (**A**) *m/z* 1,665.9308 and (**B**) *m/z* 1,551.8261 of MTX-6, using 40 eV CE.

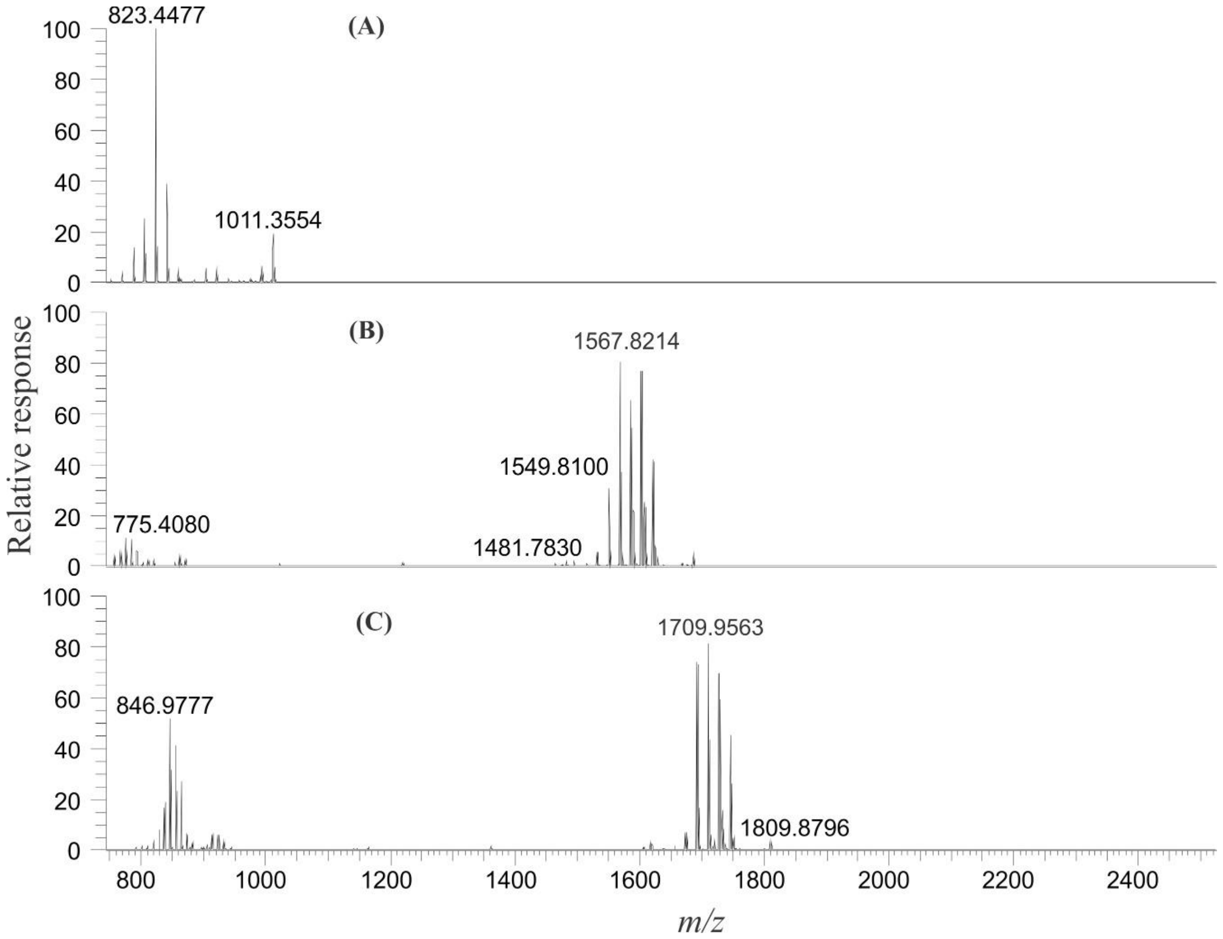

Figure S35. Full scan +ESI HR mass spectra (*m/z* 800–2,500) of the oxidative cleavage products of MTX-7 displaying (**A**) Fragment A, (**B**) ‘Fragment B’ and (**C**) ‘Fragments B and C’ connected.

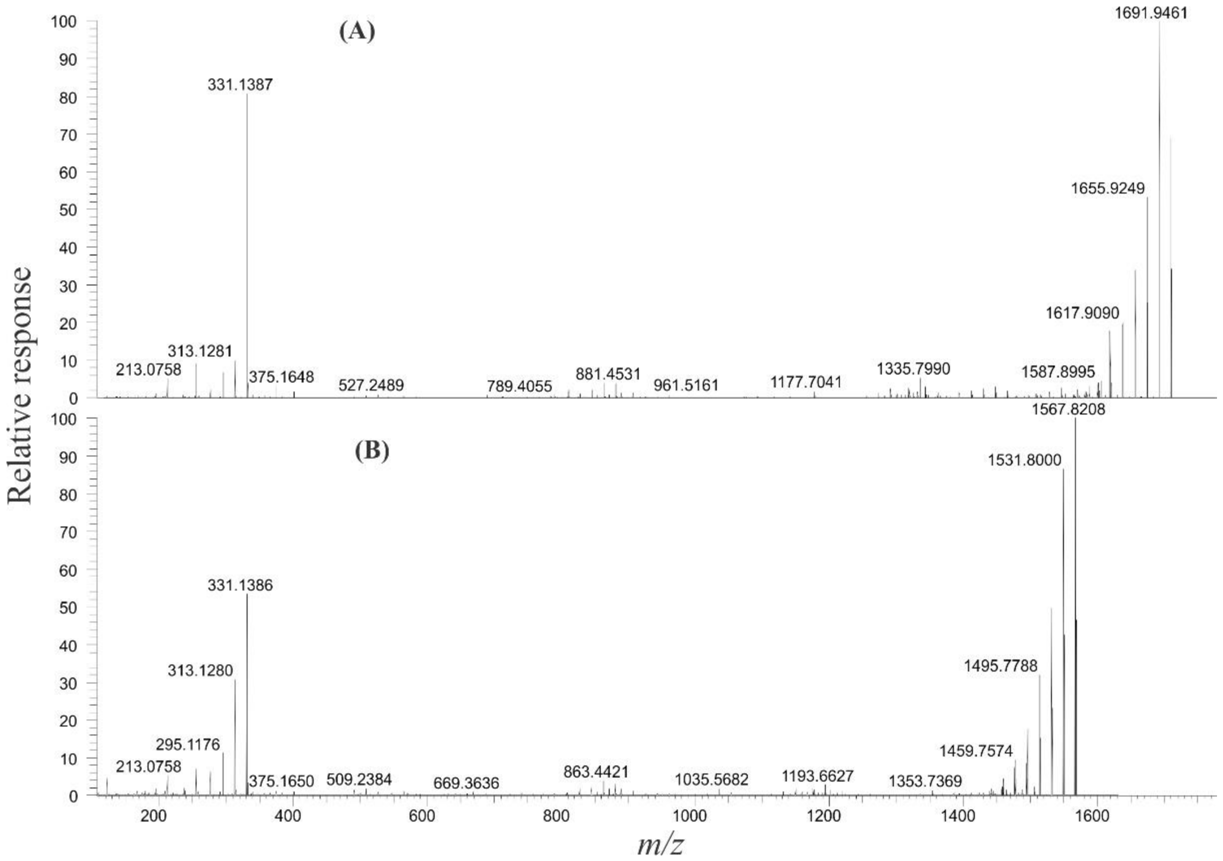

Figure S36. CID fragmentation spectra of the two unknown ions (**A**) *m/z* 1,709.9563 and (**B**) *m/z* 1,567.8214 of MTX-7, using 40 eV CE.

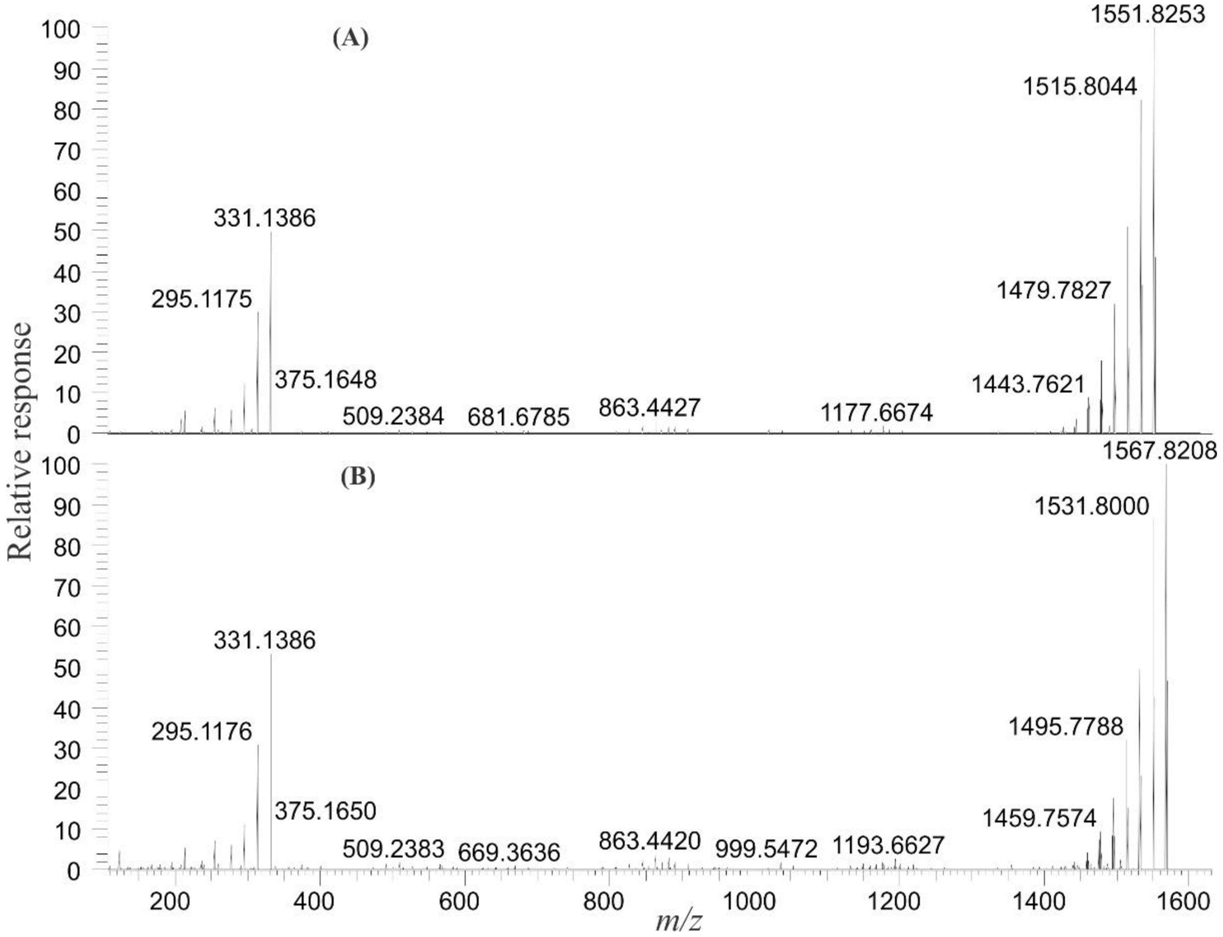

Figure S37. Comparison of the CID fragmentation spectra (40 eV CE) for the B fragments of (**A**) MTX-6 *m/z* 1,551.8253 and (**B**) MTX-7 *m/z* 1,567.8208.

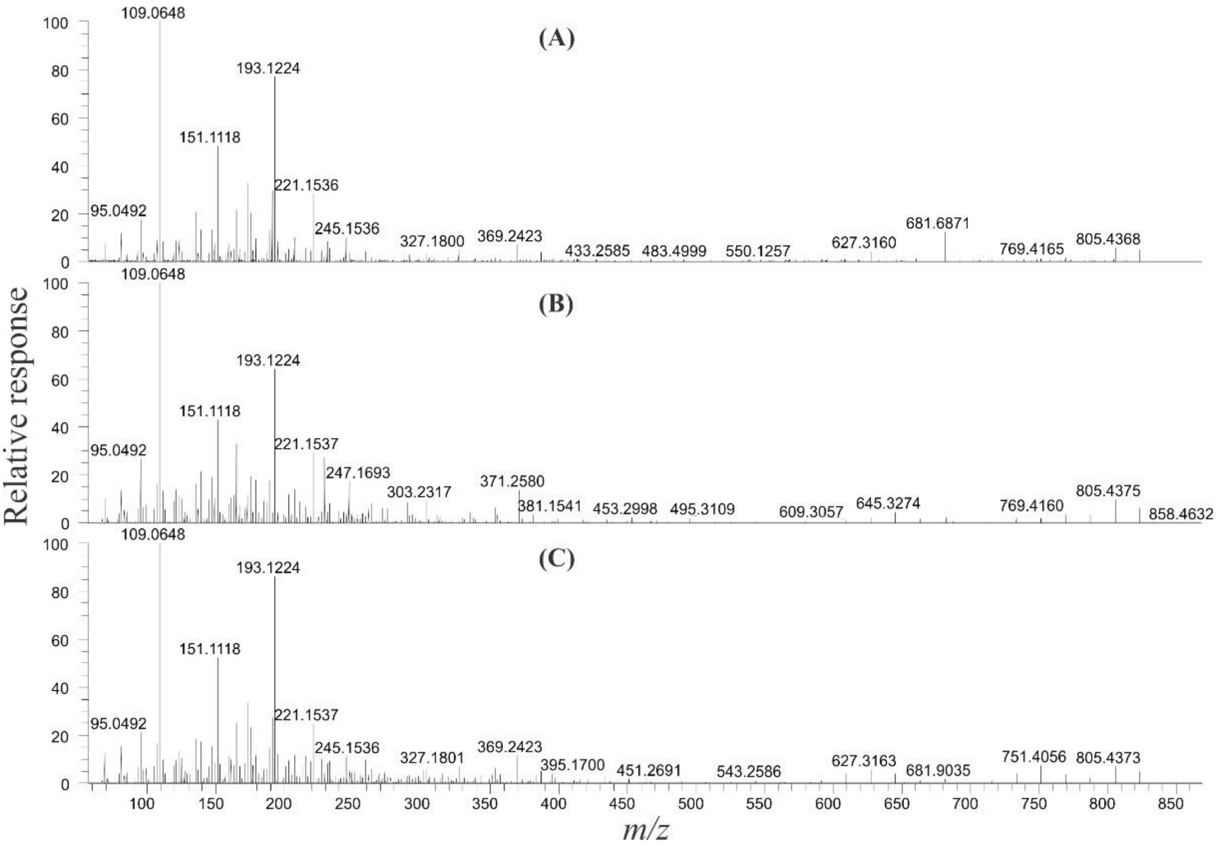

Figure S38. Fragmentation spectra of the *m/z* 823.4481 ions ($C_{43}H_{67}O_{15}$) from (**A**) MTX-1, (**B**) MTX-6 and (**C**) MTX-7.

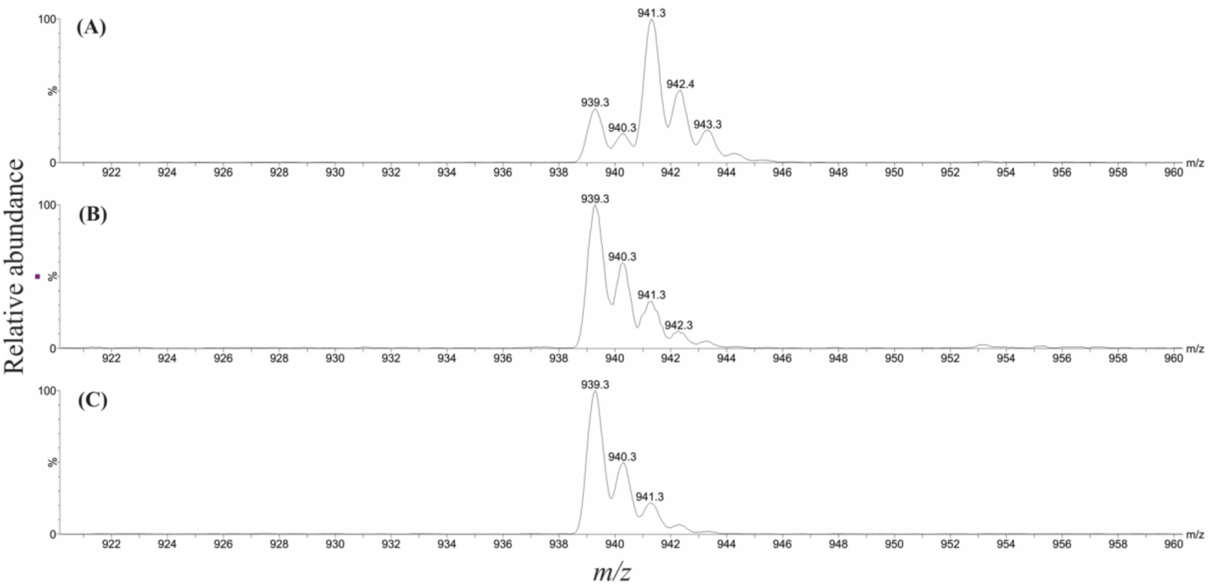

Figure S39. Reduced oxidative cleavage products of the (**A**) peak at 1.90 min, (**B**) peak at 1.92 min in MTX-1 and MTX-7, and (**C**) the peak in MTX-6 at 1.96 min.

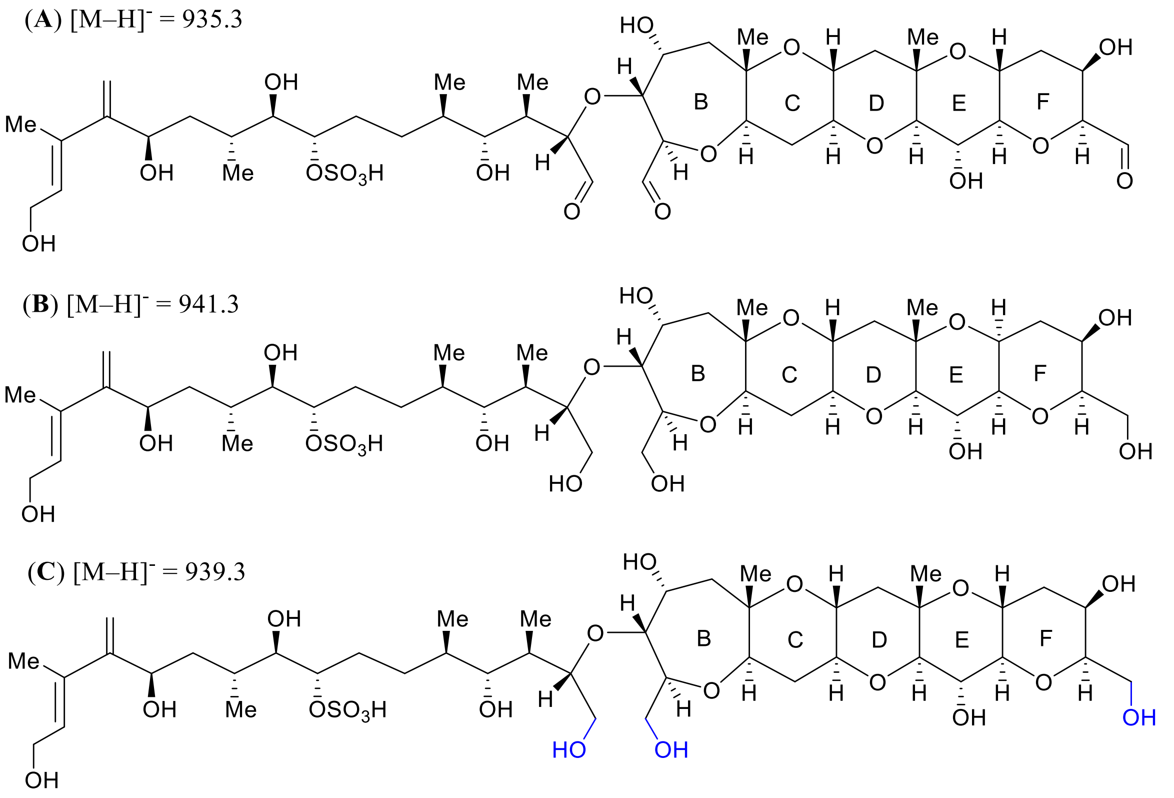

Figure S40. The (**A**) Fragment A oxidation product of MTX-1 and MTX-7 and the proposed structures for the reduced forms pertaining to the two unresolved peaks at (**B**) 1.90 min and (**C**) 1.92 min (one of the three hydroxyls in blue is an aldehyde).

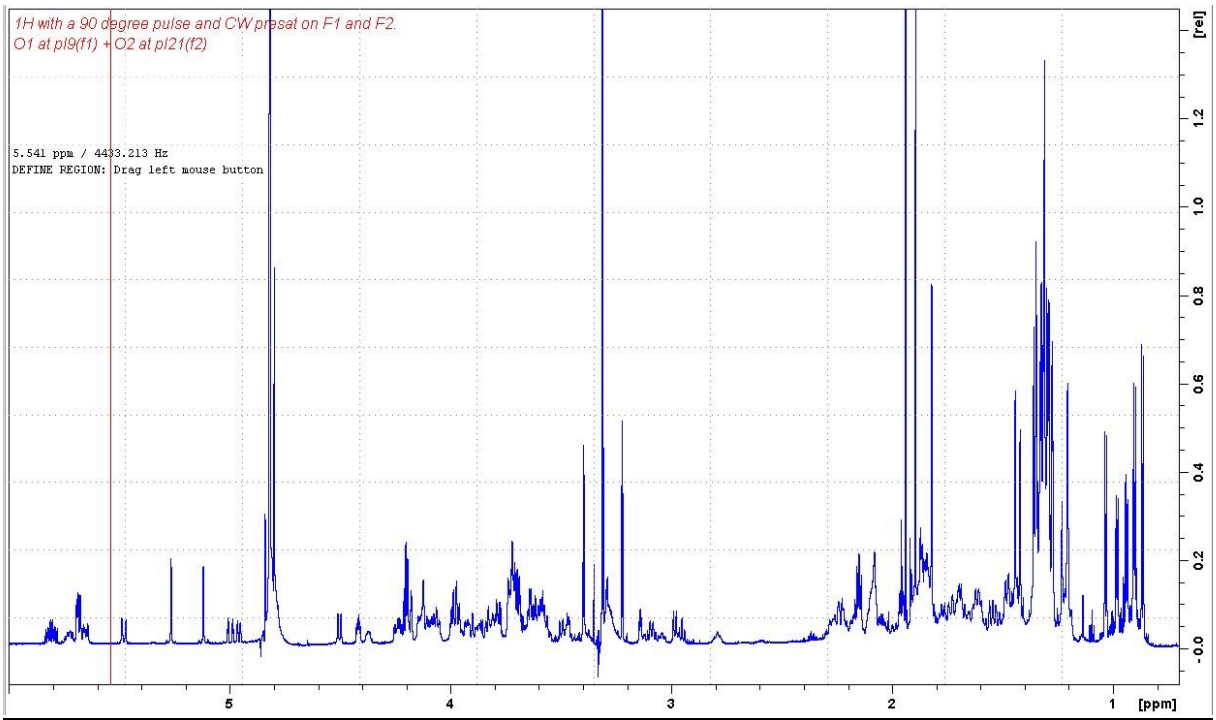

Figure S41. ^{1}H NMR spectrum of MTX-7 acquired in CD_3OD at 800 MHz.

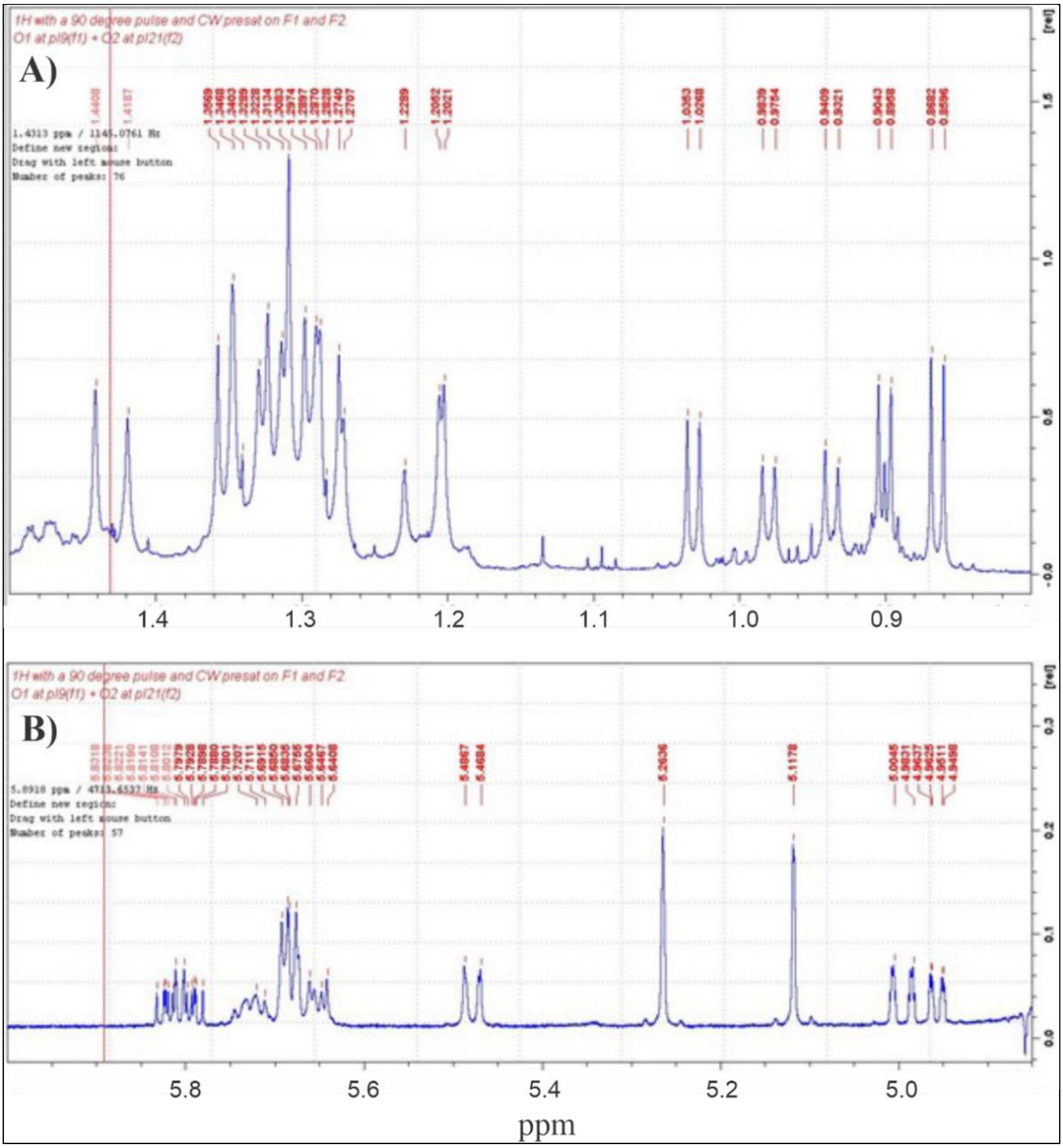

Figure S42. Expansion of the ^{1}H NMR spectrum of MTX-7 displaying (**A**) the upfield methyl groups (0.8–1.5 ppm) and (**B**) the downfield olefinic proton signals (4.9–5.9 ppm) acquired in CD_3OD at 800 MHz.

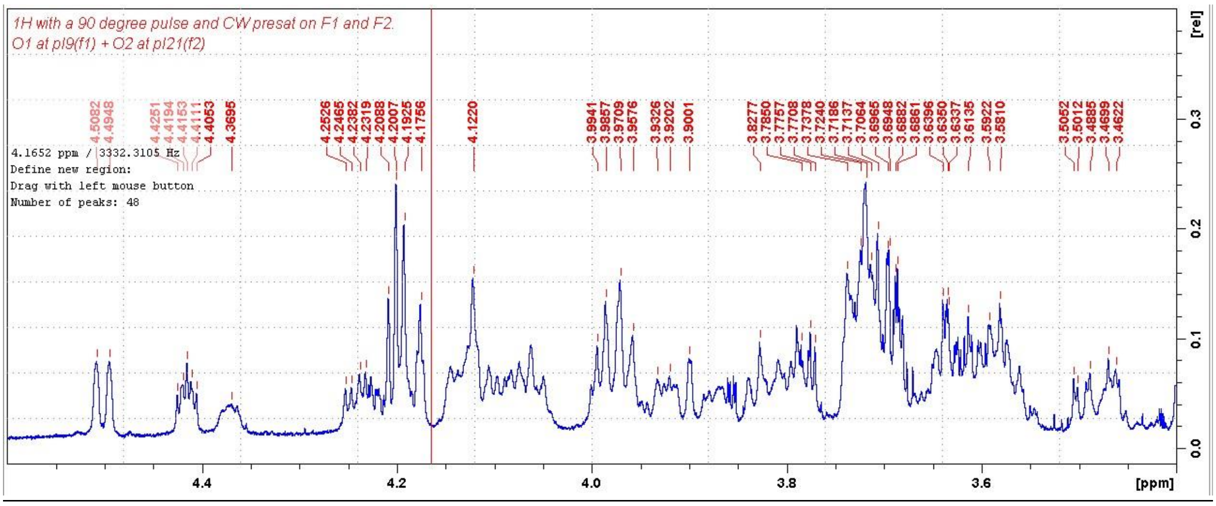

Figure S43. Expansion of the ^{1}H NMR spectrum of MTX-7 (3.5–4.5 ppm region) acquired in CD_3OD at 800 MHz.

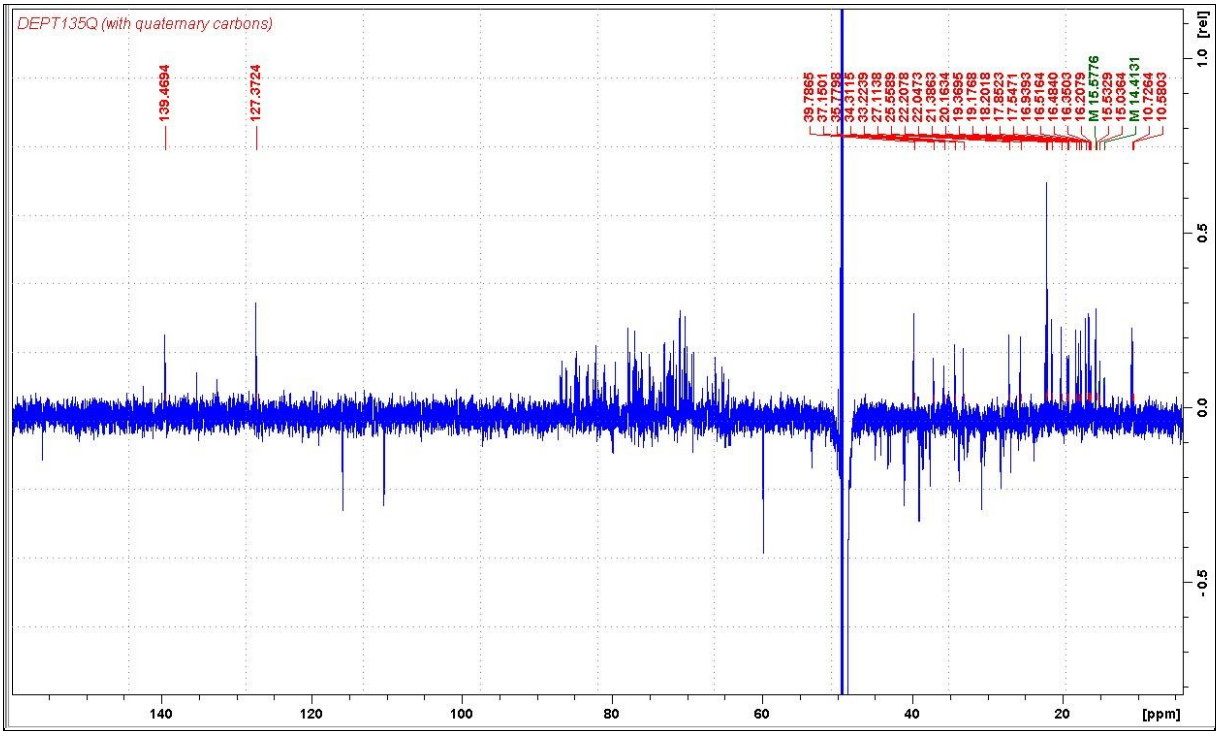

Figure S44. DEPT135Q NMR spectrum of MTX-7 acquired in CD_3OD at 200 MHz.

Table S1. The ^{1}H and ^{13}C NMR chemical shifts (ppm), multiplicity and coupling constants (Hz) of the 22 methyl groups (numbering according to their chemical shift, starting upfield) observed in MTX-7. Spectra were acquired in CD_3OD using a 800 MHz spectrometer.

**Methyl group** [a]	**δ_H (multi, *J* in Hz)**	**δ_C**	**Type**
1	0.86 (d, 6.9 Hz)	16.4	Secondary
2	0.90 (d, 6.8 Hz)	16.48	Secondary
3	0.94 (d, 6.8 Hz)	10.7	Secondary
4	0.98 (d, 6.8 Hz)	17.6	Secondary
5	1.03 (d, 6.8 Hz)	16.9	Secondary
6	1.202	10.6	Tertiary
7	1.205	15.6	Tertiary
8	1.23	15.0	Tertiary
9	1.271	16.2	Tertiary
10	1.274	22.1 [b]	Tertiary
11	1.30	17.9	Tertiary
12	1.307 [c]	20.2	Tertiary
13	1.307 [c]	21.4	Tertiary
14	1.313	19.2	Tertiary
15	1.32	18.2	Tertiary
16	1.33	16.52	Tertiary
17	1.35 [d]	25.6	Tertiary
18	1.35 [d]	27.1	Tertiary
19	1.36	22.1 [b]	Tertiary
20	1.42	19.4	Tertiary
21	1.44	22.2	Tertiary
22	1.82	15.5	Tertiary

MTX = Maitotoxin.
NMR chemical shifts are reported relative to CHD_2OD (3.31 ppm) and CD_3OD (49.0 ppm).
[a] The 22 methyl group signals are numbered according to their ^{1}H NMR chemical shift.
[b, c, d] Co-occurring signals observed for these sets of methyl groups.

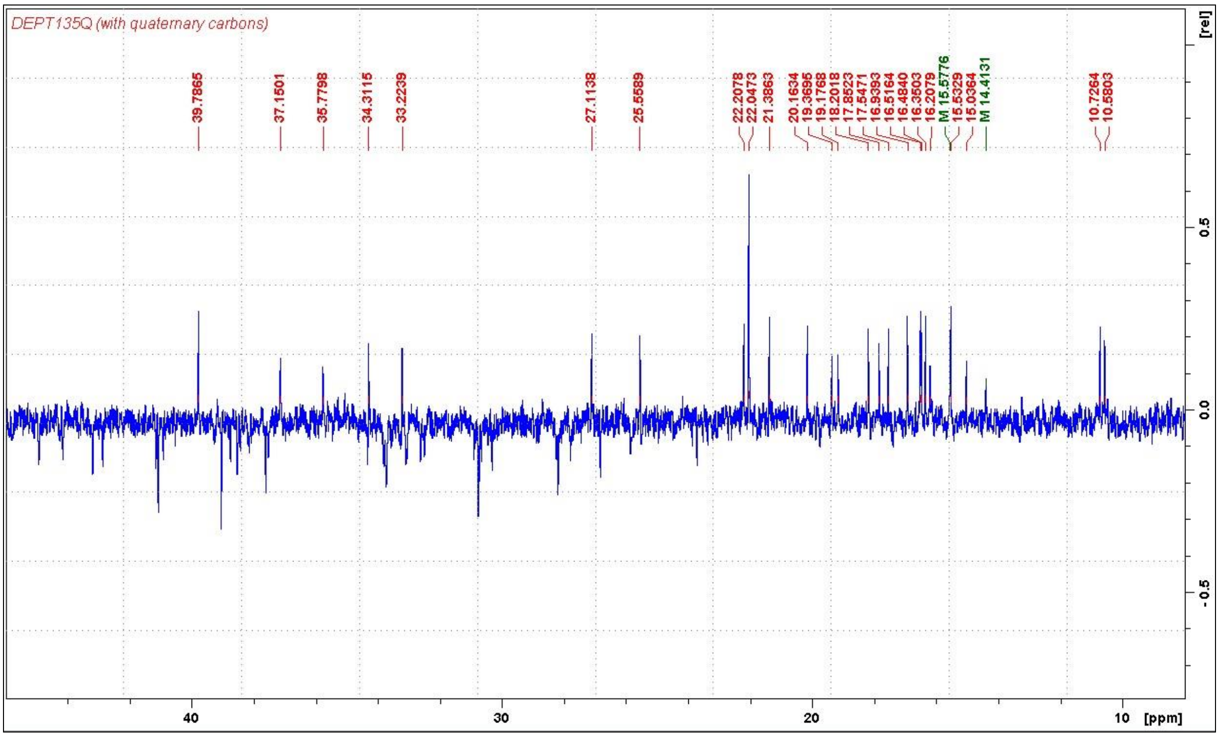

Figure S45. Expansion of the 8–42 ppm region of the DEPT135Q spectrum of MTX-7 acquired in CD_3OD at 200 MHz.

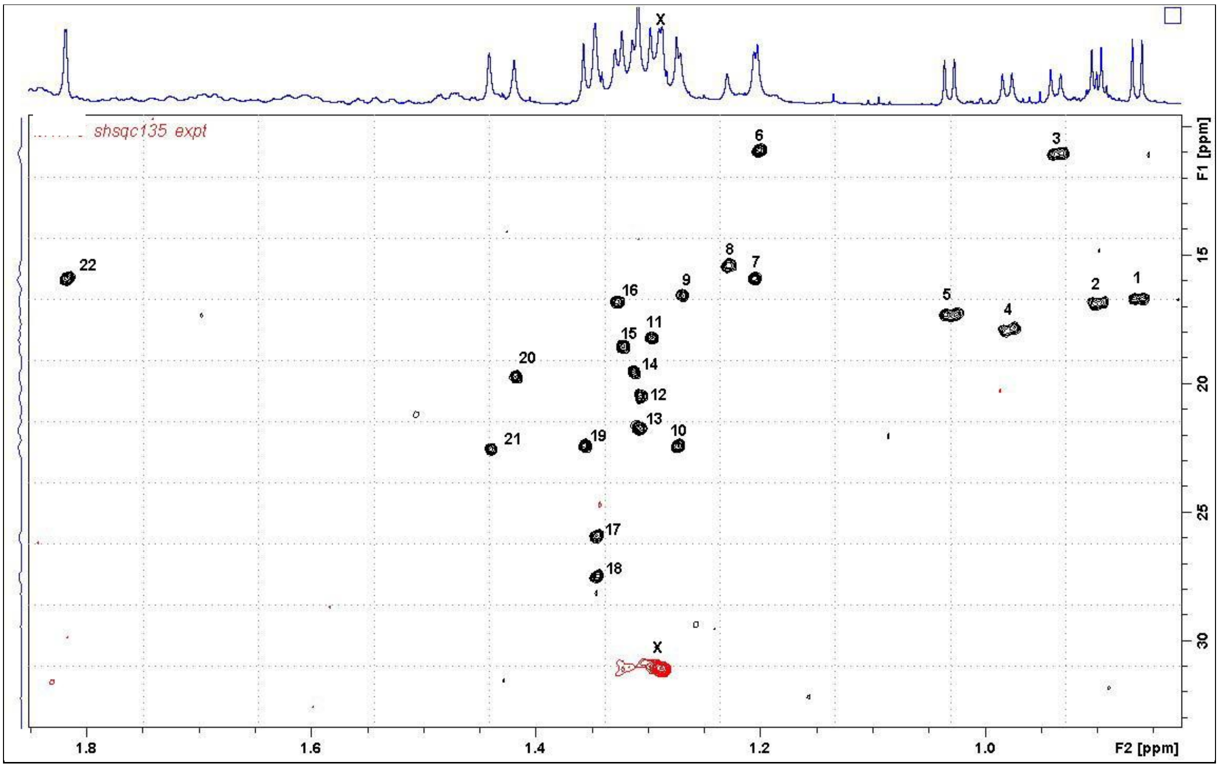

Figure S46. Expansion of the SHSQC spectrum (^{1}H: 0.8–1.8 ppm; ^{13}C: 10–30 ppm) showing the correlations determined for the 22 methyl groups (numbered according to their ^{1}H chemical shift, starting upfield) present in MTX-7 acquired in CD_3OD at 800 MHz.

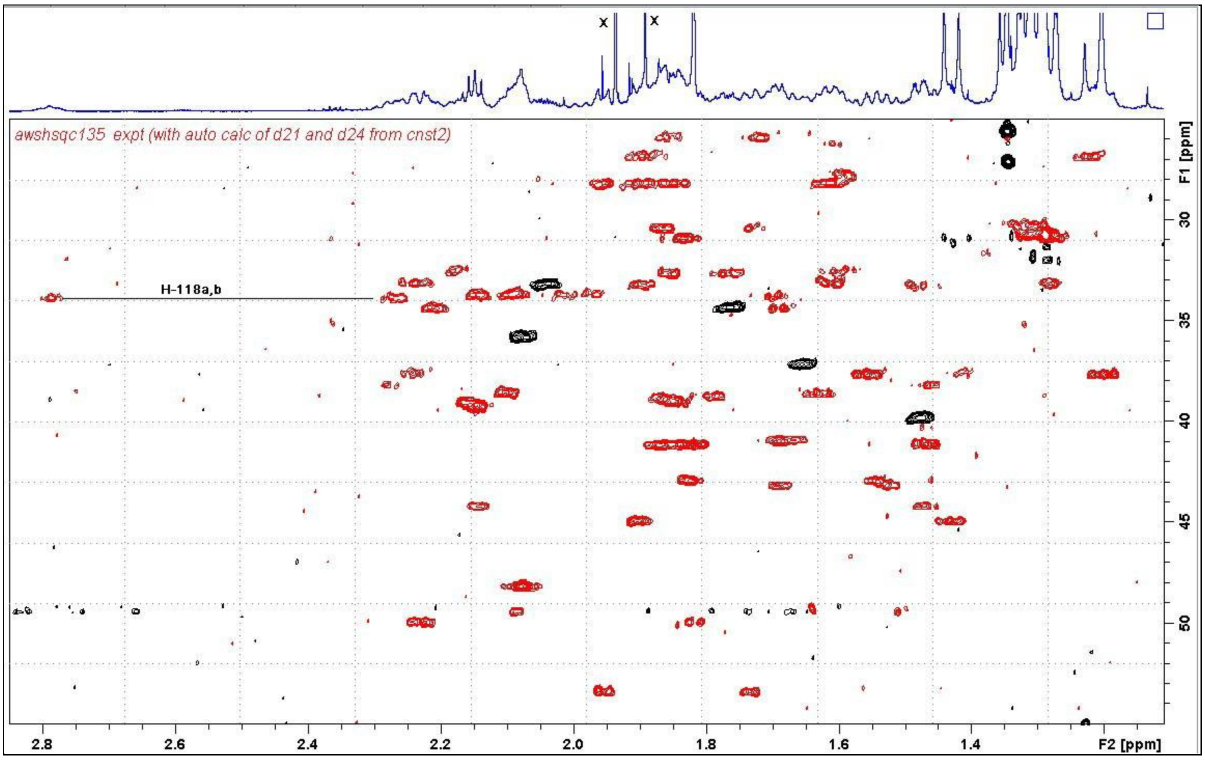

Figure S47. Expansion of the SHSQC spectrum (^{1}H: 1.2–2.8 ppm; ^{13}C: 25–55 ppm) of MTX-7 acquired in CD_3OD at 800 MHz showing the methylene proton region, including the 2.78 ppm and 2.27 ppm proton signals proposed to originate from the H-$118_{a,b}$ protons of a ring B′ (*Z*)-double bond, analogous to that published by Murata *et al.*,[1,2] for MTX-1.

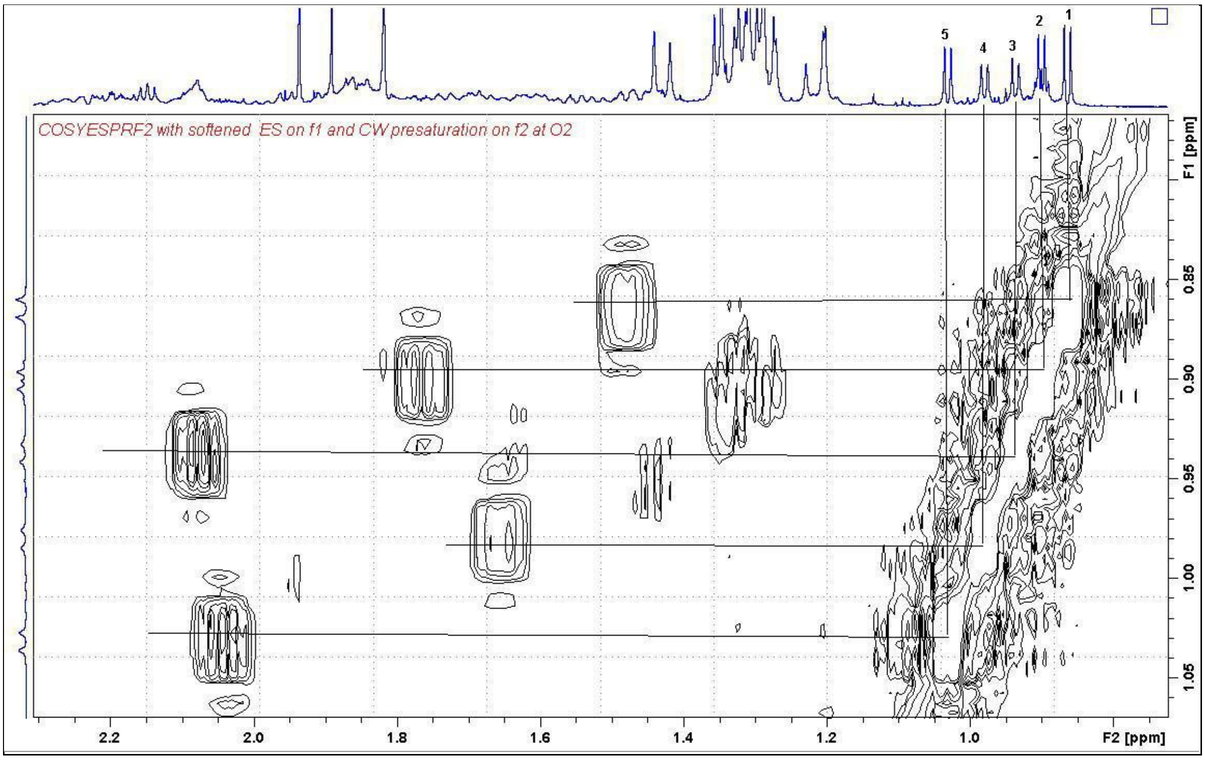

Figure S48. Expansion of the COSY spectrum (^{1}H: 0.8–1.05 ppm; ^{1}H: 0.8−2.2 ppm) of MTX-7 acquired in CD_3OD at 800 MHz depicting the methine proton correlations to the five secondary methyl groups.

Table S2. The ^{1}H and ^{13}C NMR chemical shifts (ppm) of the five methine groups and the respective secondary methyl groups (the chemical shift corresponds to the underlined atom). Spectra were acquired in CD_3OD using a 800 MHz spectrometer.

**Methine group**	**Chemical shift (ppm)**		
	**<u>C</u>H(CH₃)**	**C<u>H</u>(CH₃)**	**CH(C<u>H</u>₃)**
1	39.8	1.48	0.86
2	34.3	1.77	0.90
3	35.8	2.08	0.94
4	37.2	1.65	0.98
5	33.1	2.04	1.03

NMR chemical shifts are reported relative to CHD_2OD (3.31 ppm) for ^{1}H signals and CD_3OD (49.0 ppm) for ^{13}C signals.

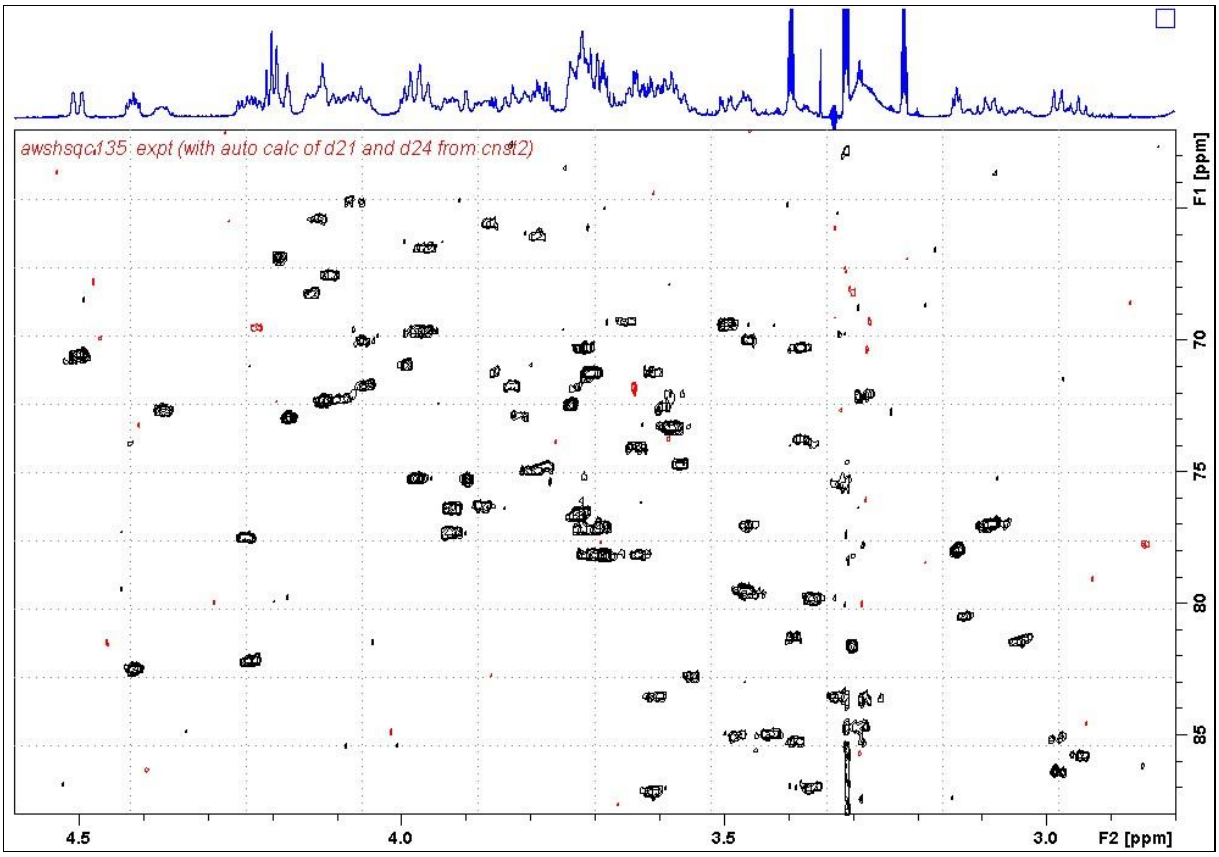

Figure S49. Expansion of the SHSQC spectrum (^{1}H: 2.9–4.5 ppm; ^{13}C: 62−88 ppm) of MTX-7 acquired in CD_3OD at 800 MHz showing correlations arising from the oxygenated methine signals.

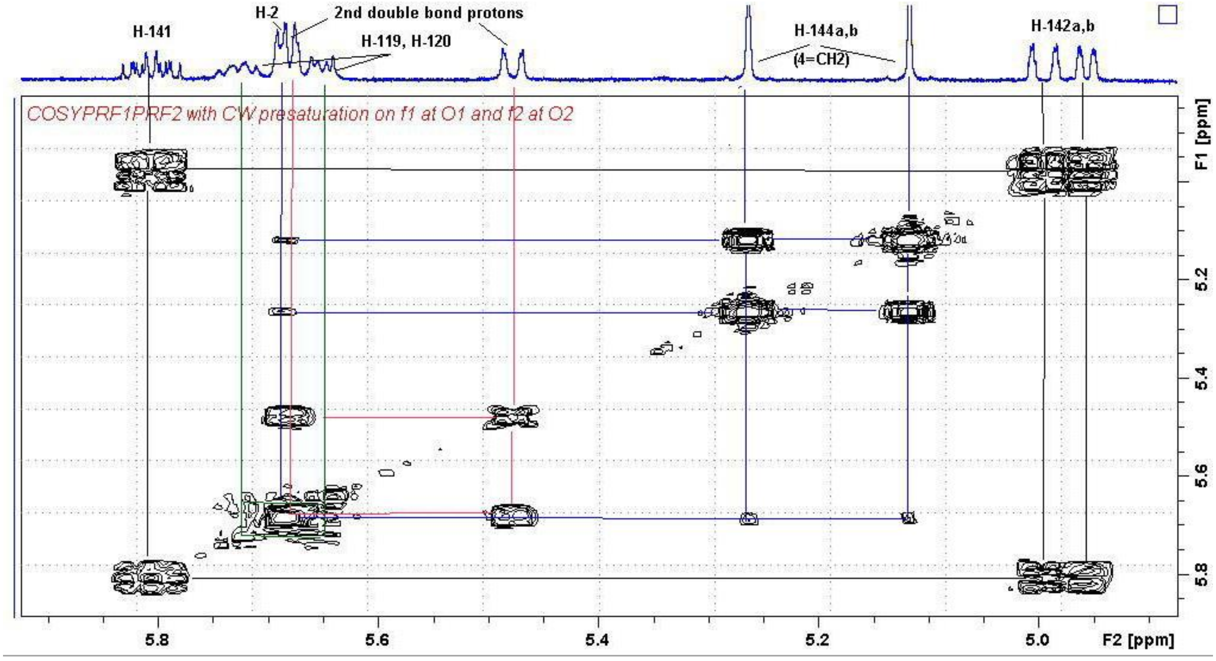

Figure S50. Expansion of the COSY spectrum (^{1}H: 4.7–5.8 ppm; ^{1}H: 4.9−5.8 ppm) of MTX-7 acquired in CD_3OD at 800 MHz showing the five sets of olefinic signals (individual protons or groups), with assignments based on those published for MTX-1 by Murata *et al*.[2]

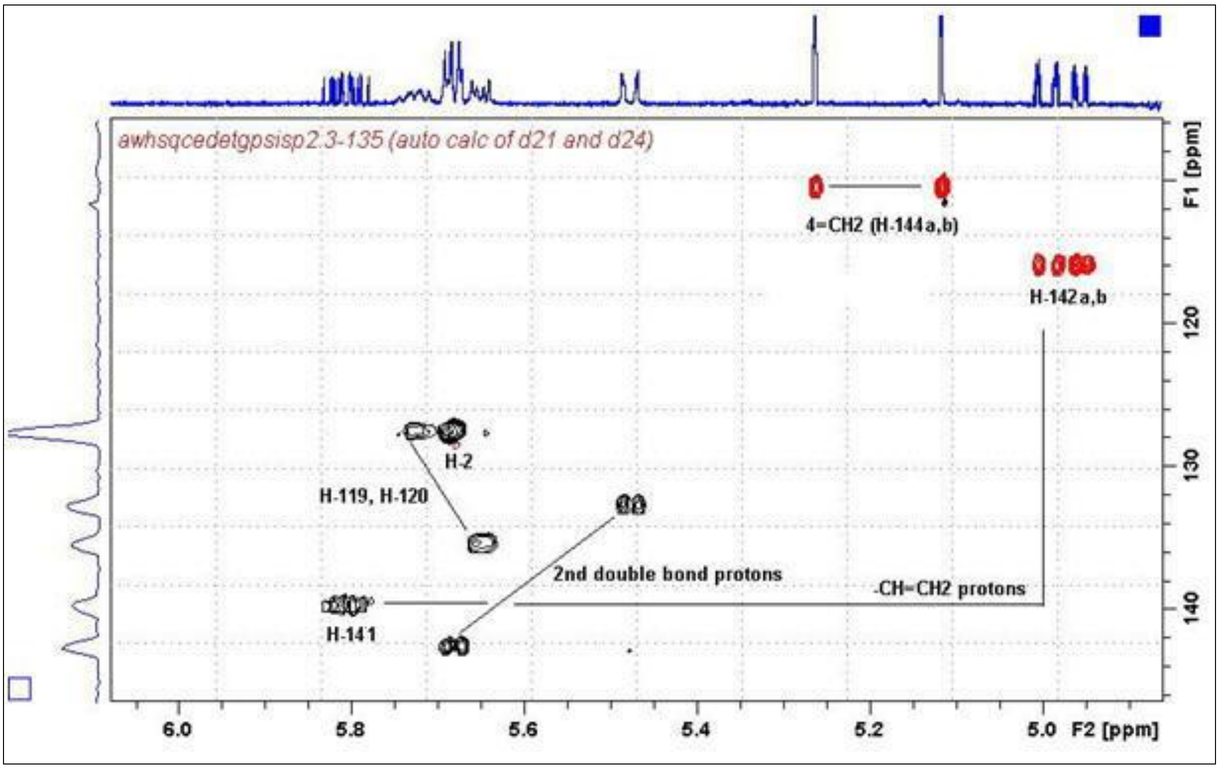

Figure S51. Expansion of the HSQC spectrum (^{1}H: 4.9–6.0 ppm; ^{13}C: 100−150 ppm) of MTX-7 acquired in CD_3OD at 800 MHz showing the five individual or pairs of olefinic protons, with assignments based on those published for MTX-1 by Murata *et al*.[2]

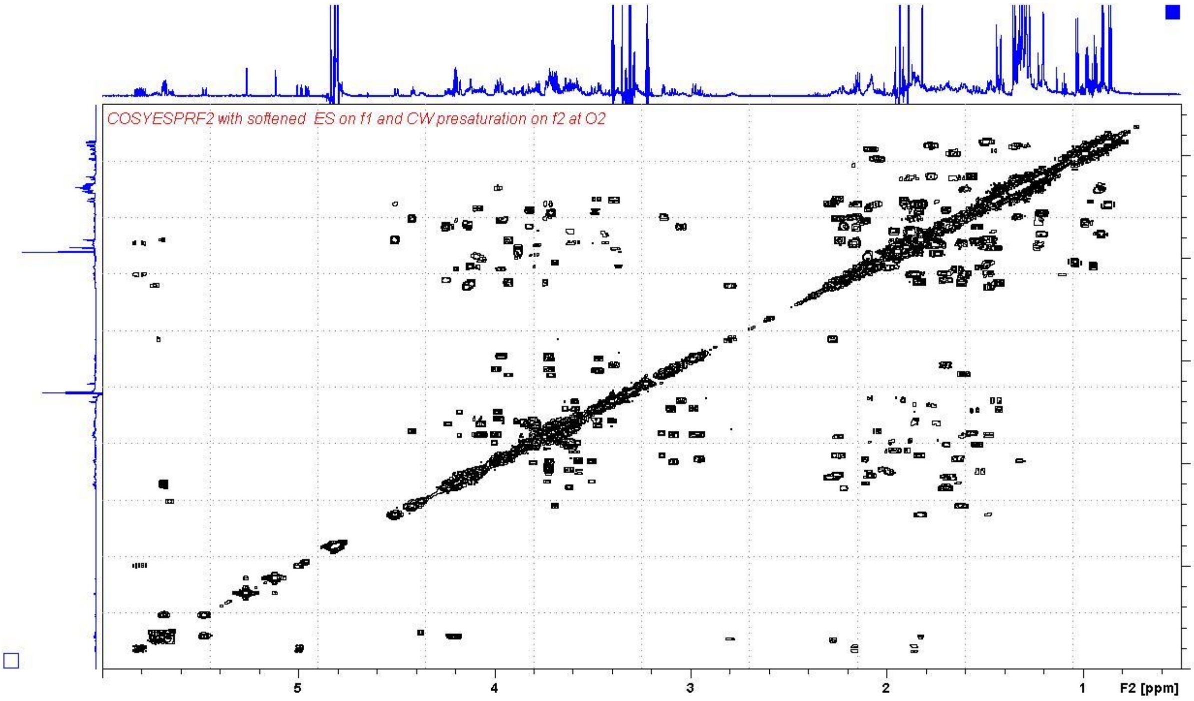

Figure S52. COSY NMR spectrum of MTX-7 acquired in CD_3OD at 800 MHz.

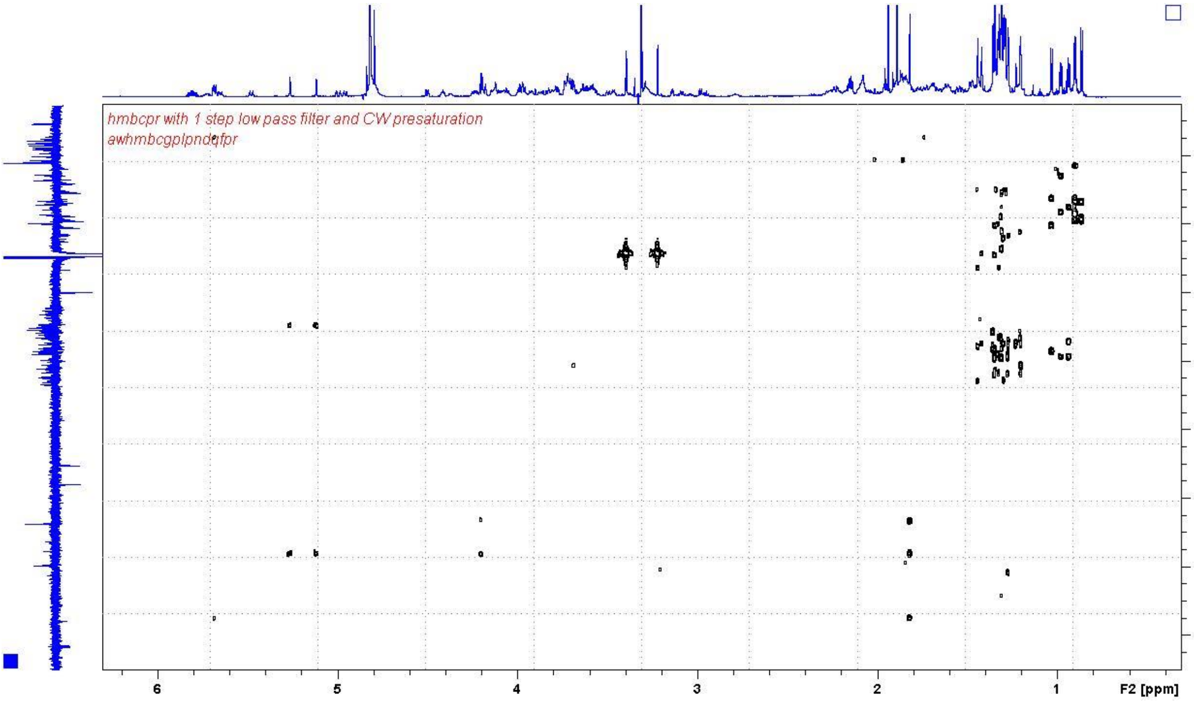

Figure S53. HMBC NMR spectrum of MTX-7 acquired in CD_3OD at 800 MHz.

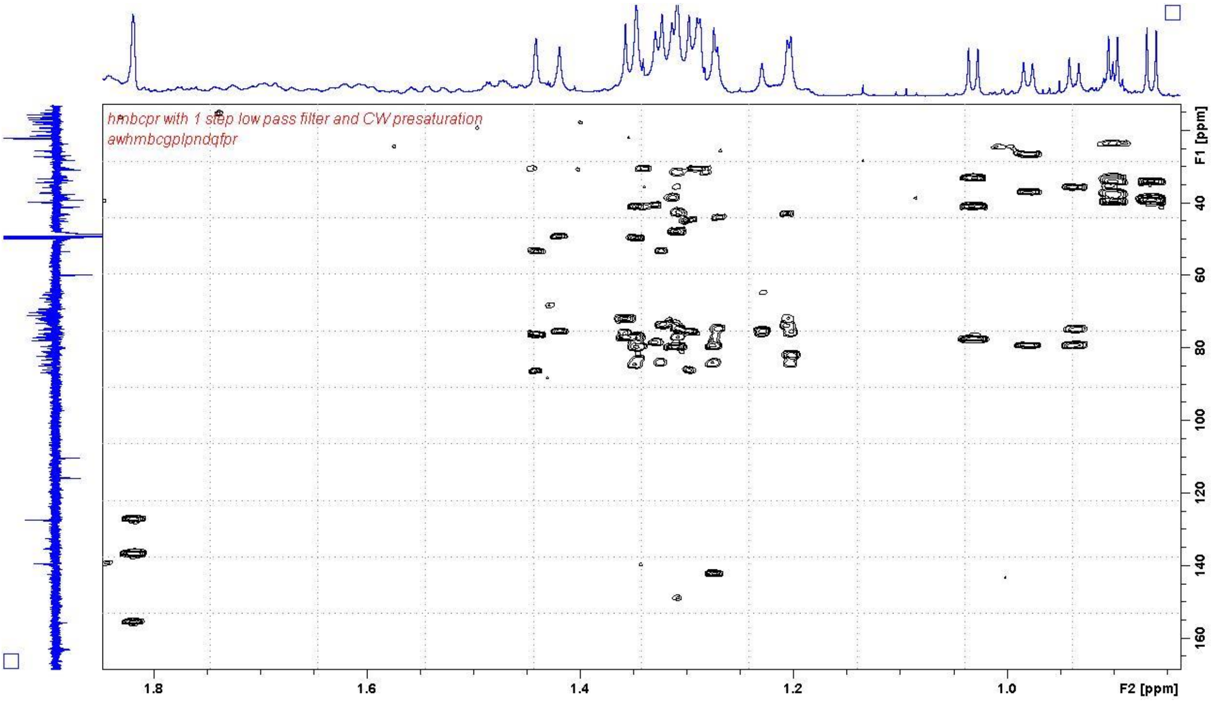

Figure S54. Expansion of the HMBC NMR spectrum of MTX-7 (^{1}H: 0.8–1.8 ppm; ^{13}C: 20–140 ppm) acquired in CD_3OD at 800 MHz.

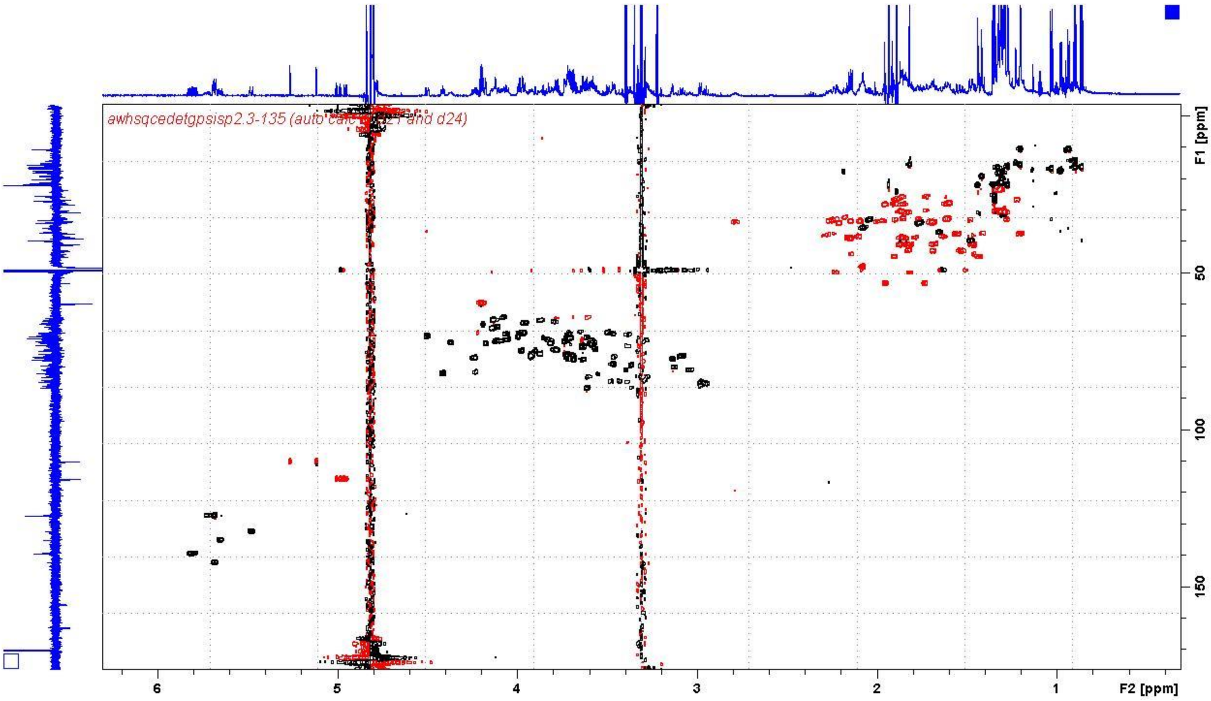

Figure S55. HSQC NMR spectrum of MTX-7 acquired in CD_3OD at 800 MHz.

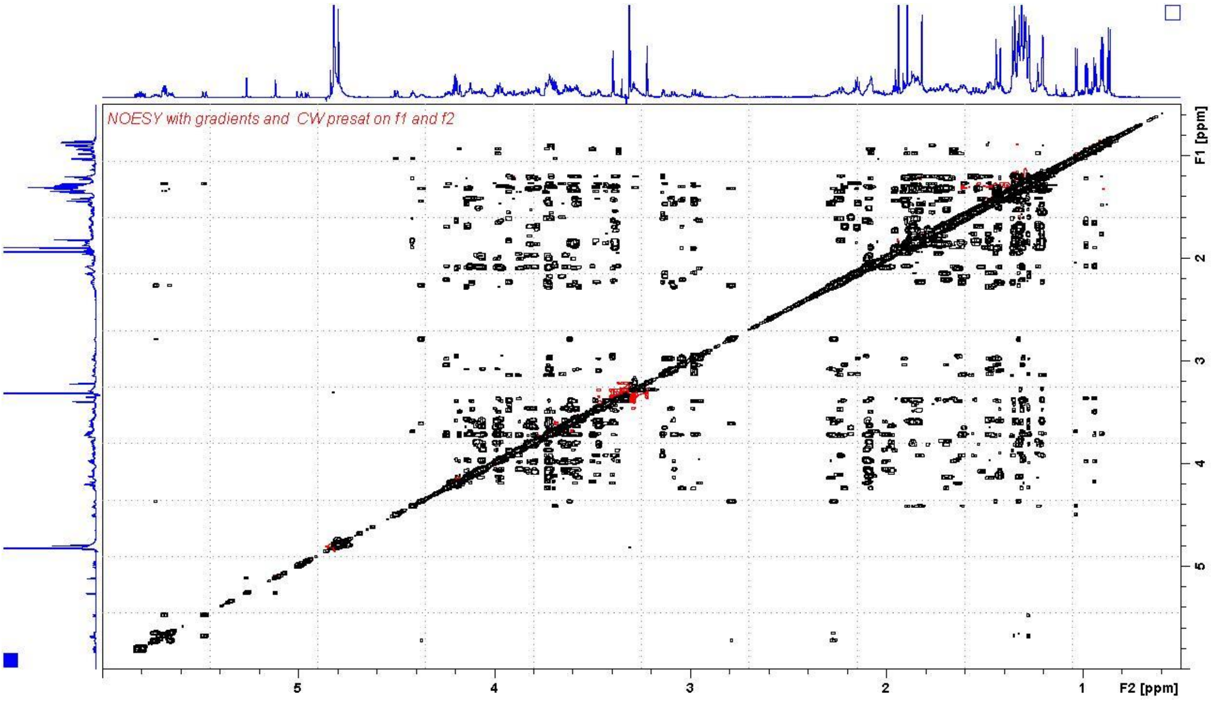

Figure S56. NOESY NMR spectrum of MTX-7 acquired in CD_3OD at 800 MHz.

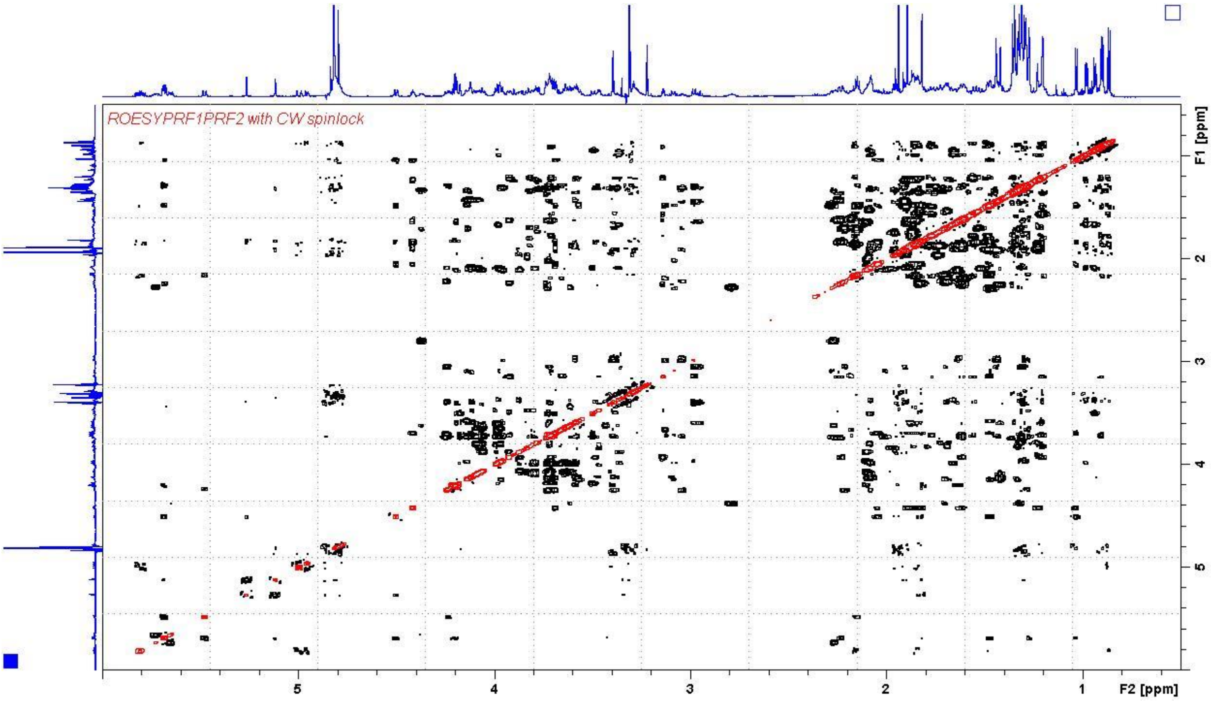

Figure S57. ROESY NMR spectrum of MTX-7 acquired in CD_3OD at 800 MHz.

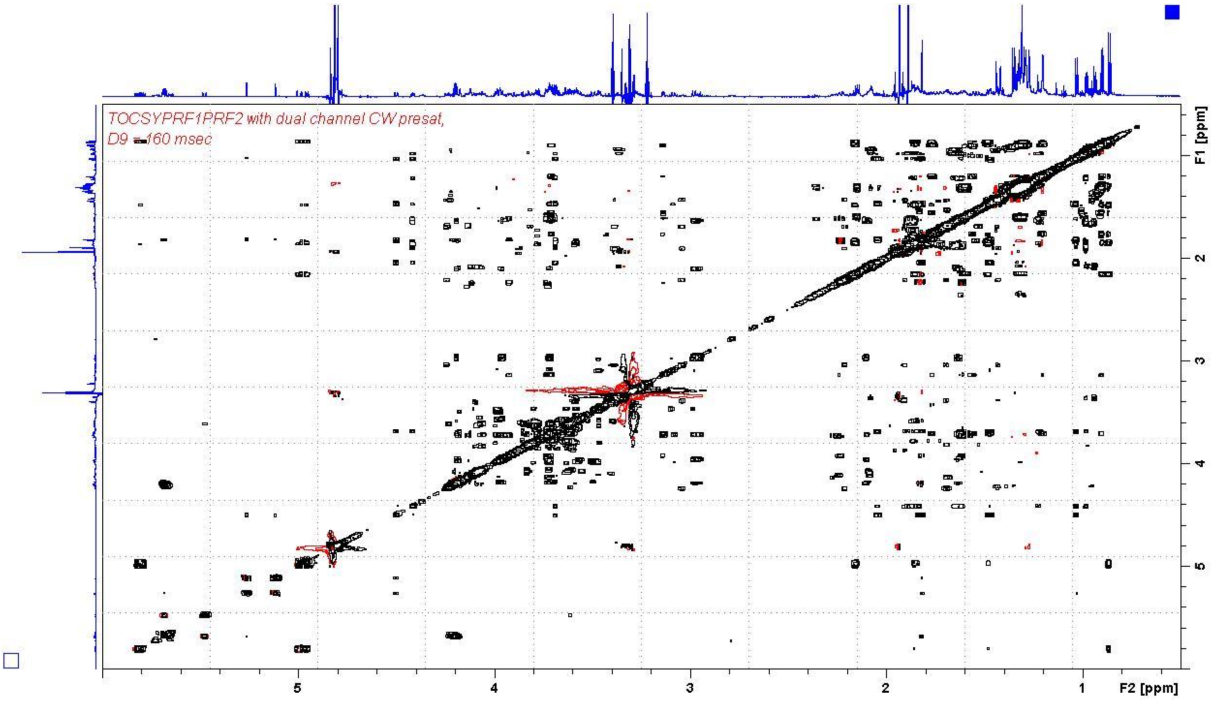

Figure S58. TOCSY NMR spectrum of MTX-7 acquired in CD_3OD at 800 MHz.

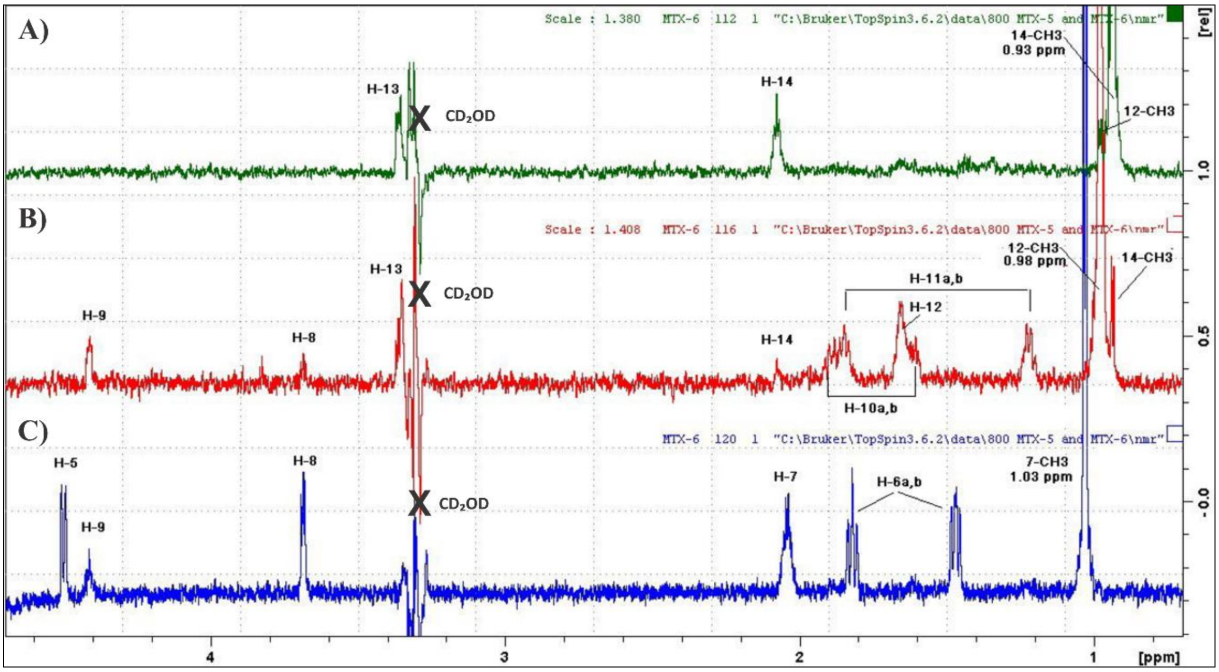

Figure S59. 160 msec 1D-SELTOCSY spectra used to assign the three ring A secondary methyl groups (**A**) 0.936 ppm (14-CH_3), (**B**) 0.979 ppm (12-CH_3) and (**C**) 1.031 ppm (7-CH_3) in MTX-7 acquired in CD_3OD at 800 MHz.

Table S3. The ^{1}H and ^{13}C NMR chemical shifts (ppm; 800 MHz) of the aliphatic side chain atoms connected to ring A in MTX-7. Spectra were acquired in CD_3OD using a 800 MHz spectrometer.

**Atom**	**δ_H**	**δ_C**
1	4.20 (2H)	59.6
2	5.68	127.3
3	–	155.7
4	–	137.3
5	4.50	70.0
6	1.47, 1.82	41.1
7	2.04	33.2
8	3.69	77.8
9	4.41	81.7
10	1.61, 1.89	33.5
11	1.22, 1.87	26.8
12	1.65	37.2
13	3.35	79.5
14	2.08	35.8
15	3.97 [a]	74.9
3-CH_3 (C-143)	1.82	15.5
4=CH_2 (C-144)	5.12, 5.26	110.4
7-CH_3 (C-145)	1.03	16.9
12-CH_3 (C-146)	0.98	17.6
14-CH_3 (C-147)	0.94	10.7

MTX = Maitotoxin.
NMR chemical shifts are reported relative to CHD_2OD (3.31 ppm) for ^{1}H signals and CD_3OD (49.0 ppm) for ^{13}C signals.
[a] Tentative assignment based on the HMBC correlation observed.

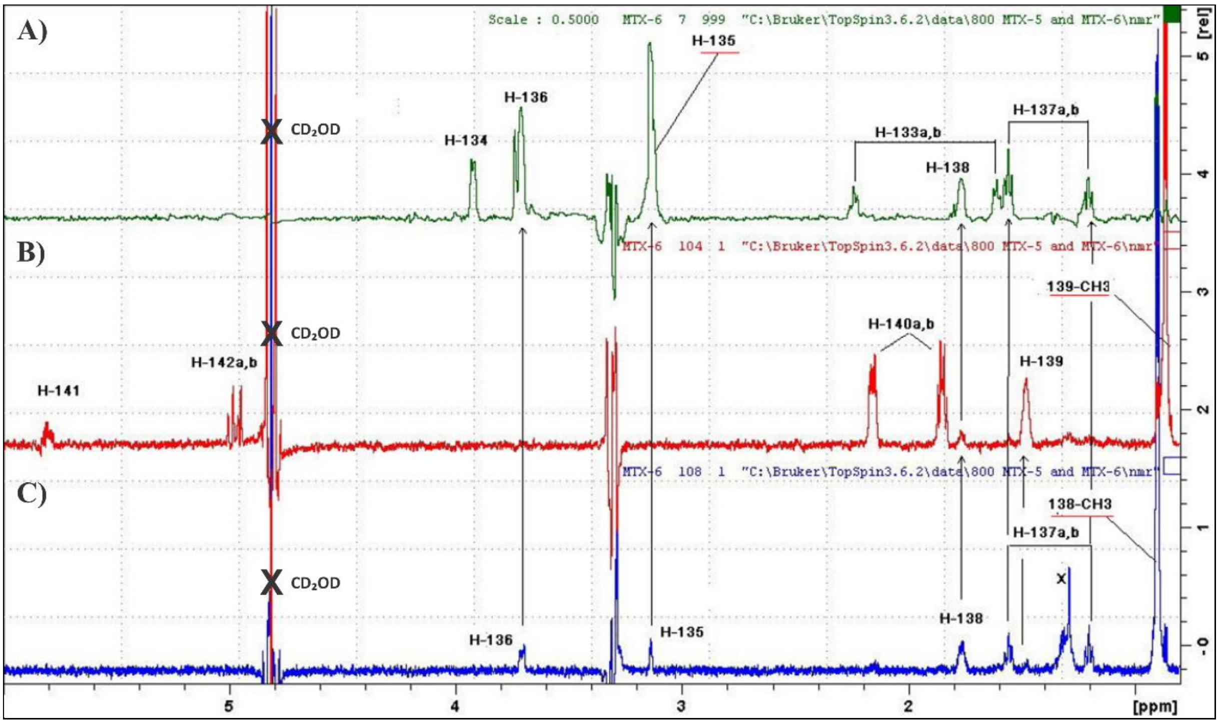

Figure S60. (**A**) 3.13 ppm H-135 slice from the 160 msec 2D-TOCSY spectrum, and the 160 msec 1D-SELTOCSY spectra acquired for the two ring F′ secondary methyl groups (**B**) 139-CH_3 (0.863 ppm) and (**C**) 138-CH_3 (0.900 ppm) in MTX-7 acquired in CD_3OD at 800 MHz.

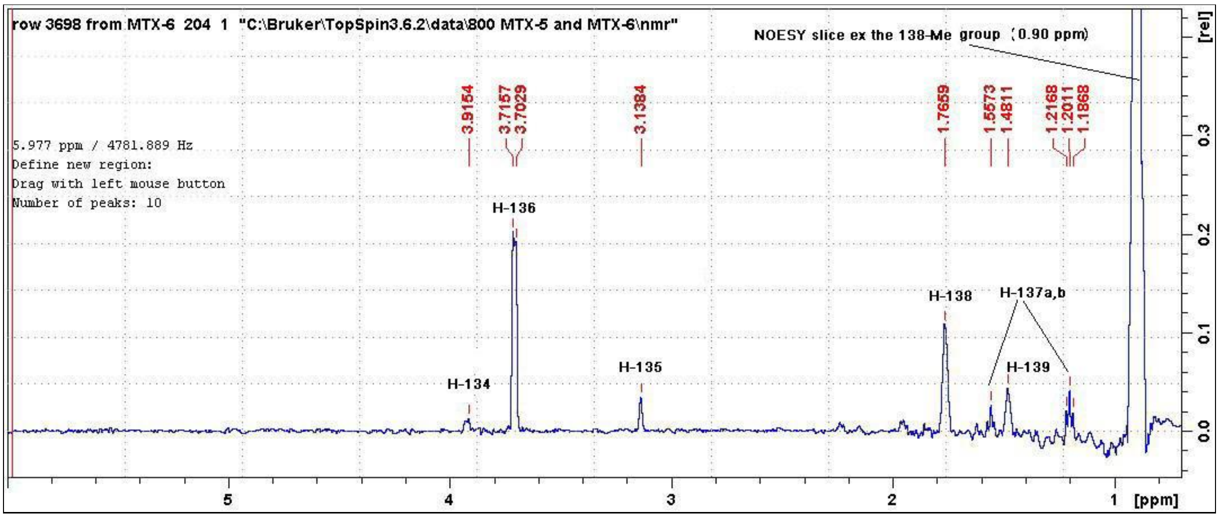

Figure S61. A NOESY slice ex the 138-CH_3 (0.900 ppm) methyl group protons of MTX-7 acquired in CD_3OD at 800 MHz showing correlations to H-134 (3.92 ppm), H-135 (3.31 ppm), H-136 (3.70 ppm), H-137$_{a,b}$ (1.20, 1.56 ppm), H-138 (1.76 ppm) and H-139 (1.48 ppm) protons.

Table S4. The ^{1}H and ^{13}C NMR chemical shifts (ppm) of the aliphatic side chain atoms connected to ring F′ in MTX-7. Spectra were acquired in CD_3OD using a 800 MHz spectrometer.

**Atom**	**δ_H**	**δ_C**
134	3.93	75.8 or 76.7 [a]
135	3.13	77.4
136	3.70	– [b]
137	1.20, 1.56	37.6
138	1.76	34.3
139	1.48	39.8
140	1.85, 2.15	39.1
141	5.81	139.6
142	4.96, 4.99	115.6
163 (138-CH_3)	0.90 (3H)	16.5
164 (139-CH_3)	0.86 (3H)	16.4

MTX = Maitotoxin.
NMR chemical shifts are reported relative to CHD_2OD (3.31 ppm) for ^{1}H signals and CD_3OD (49.0 ppm) for ^{13}C signals.
[a] HSQC and SHSQC spectra showed two coincident proton signals occurred at 3.93 ppm, therefore the C-134 carbon chemical shift could not be assigned.
[b] HSQC and SHSQC spectra showed six coincident proton signals occurred at 3.70 ppm, therefore the C-136 carbon chemical shift could not be determined.

Table S5. The ^{1}H and ^{13}C NMR chemical shifts (ppm) and the HMBC ^{13}C correlations observed for the 22 methyl groups (numbered according to their chemical shift, starting upfield) in MTX-7. Spectra were acquired in CD_3OD using a 800 MHz spectrometer.

**Methyl group**	**δ_H (multi, *J* in Hz)**	**δ_C**	**HMBC ^{13}C correlations**
1	0.86 (d, 6.9 Hz)	16.4	34.3, 39.1, 39.8
2	0.90 (d, 6.8 Hz)	16.48	34.3, 37.6, 39.8
3	0.94 (d, 6.8 Hz)	10.7	35.8, 74.9, 79.5
4	0.98 (d, 6.8 Hz)	17.6	26.8, 37.2, 79.5
5	1.03 (d, 6.8 Hz)	16.9	33.2, 41.1, 77.8
6	1.202	10.6	76.0, 81.9, 84.8
7	1.205	15.6	43.1, 71.8, 74.2
8	1.23	15.0	75.0 [a], 76.4
9	1.271	16.2	44.0, 79.1, 80.1
10	1.274	22.1 [b]	79.8, 84.5, 142.2
11	1.30	17.9	44.9, 57.7, 86.0
12	1.307 [c]	20.2	43.1 [c], 73.8 [c], 77.5 [c]
13	1.307 [c]	21.4	48.1 [c], 75.0 [c], 79.9 [c]
14	1.313	19.2	38.7, 72.5, 79.9 [c]
15	1.32	18.2	53.3, 73.8, 84.3
16	1.33	16.52	40.9, 74.5, 78.6
17	1.35 [d]	25.6	41.1 [d], 77.0 [d], 83.2 [d]
18	1.35 [d]	27.1	49.9 [d], 80.0 [d], 84.9 [d]
19	1.36	22.1 [b]	72.1, 76.0, 77.3
20	1.42	19.4	49.3, 75.7, 81.3
21	1.44	22.2	53.3, 76.4, 86.7
22	1.82	15.5	126.9, 136.6, 155.4

HMBC = Heteronuclear multiple bond correlation; MTX = maitotoxin.
NMR chemical shifts are reported relative to CHD_2OD (3.31 ppm) for ^{1}H signals and CD_3OD (49.0 ppm) for ^{13}C signals.
[a] Correlation believed to arise from two carbon signals with identical chemical shifts.
[b, c, d] HMBC correlations are interchangeable.

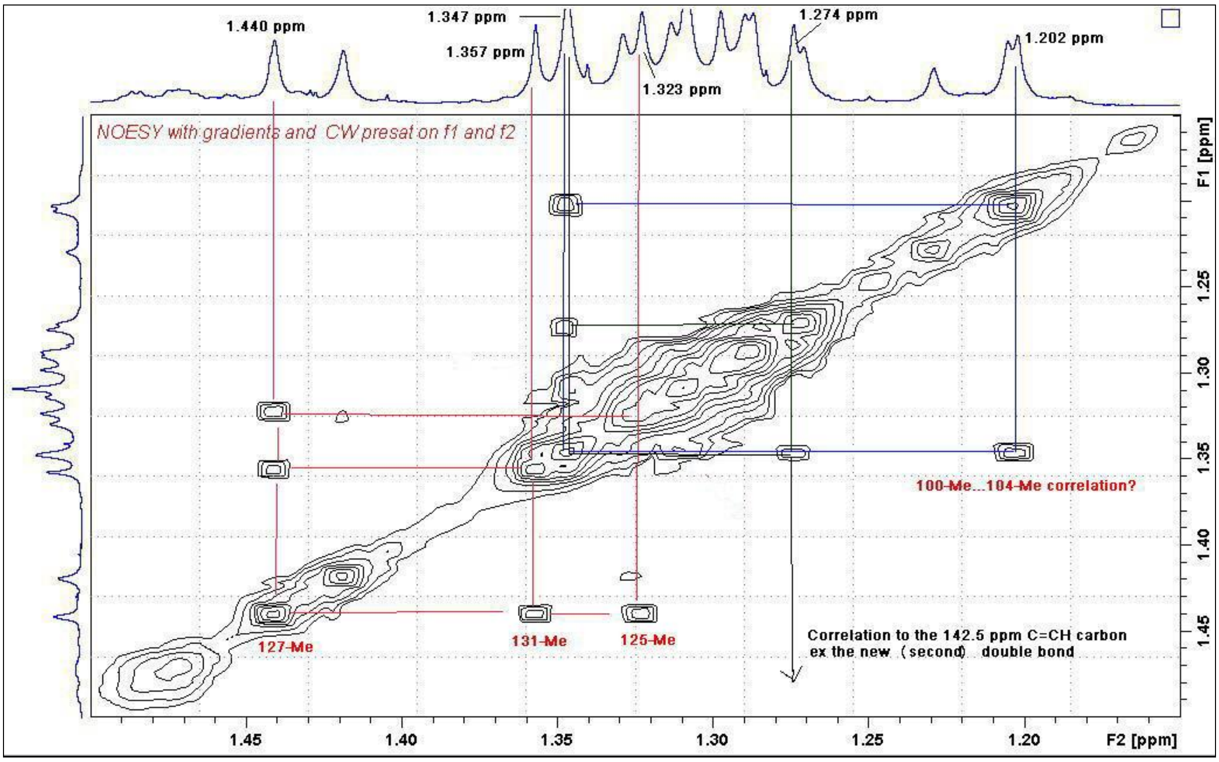

Figure S62. Expansion of the NOESY spectrum (^{1}H: 1.15–1.50 ppm; ^{1}H: 1.15−1.50 ppm) of MTX-7 acquired in CD_3OD at 800 MHz displaying the three sets of mutual NOESY correlations pertaining to the three methyl groups located in rings C′–F′.

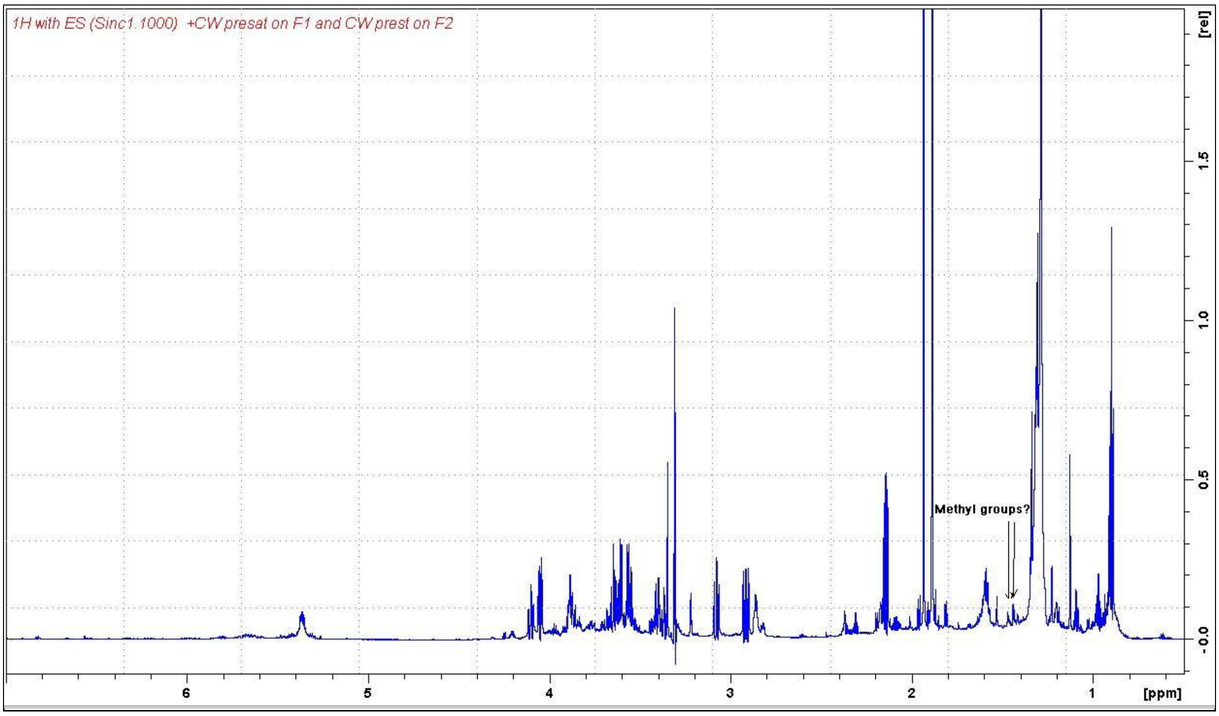

Figure S63. ^{1}H NMR spectrum of MTX-6 acquired in acquired in CD_3OD at 800 MHz.

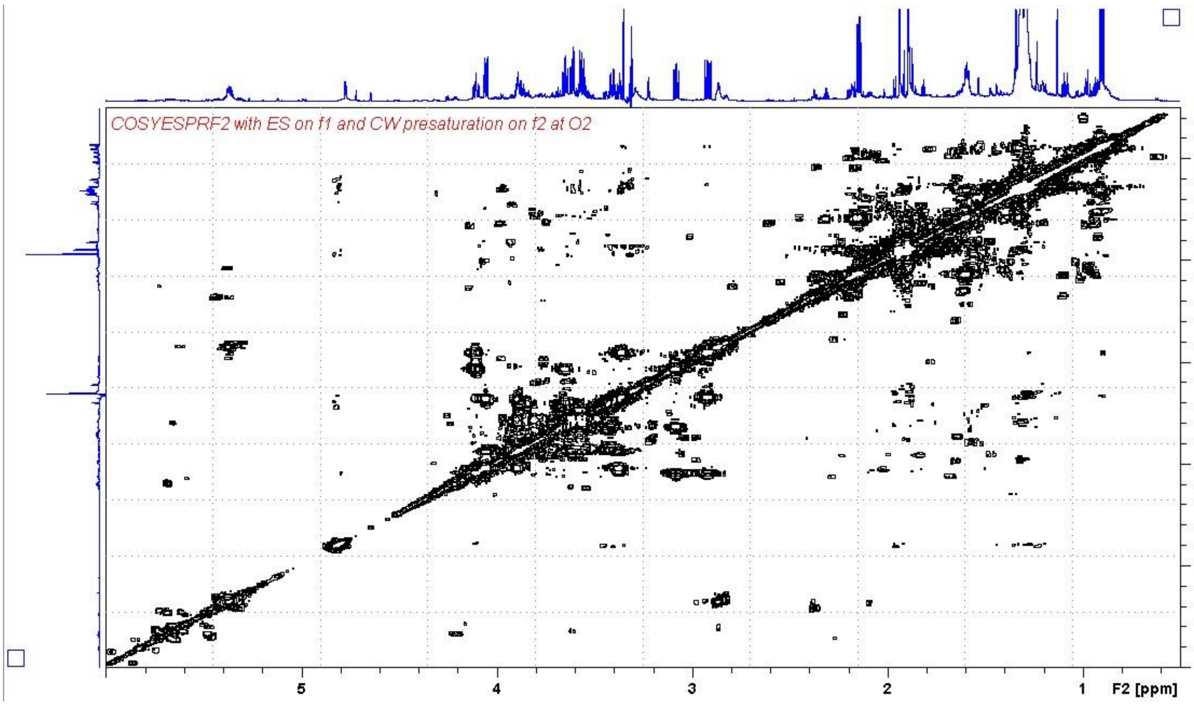

Figure S64. COSY NMR spectrum of MTX-6 acquired in CD_3OD at 800 MHz.

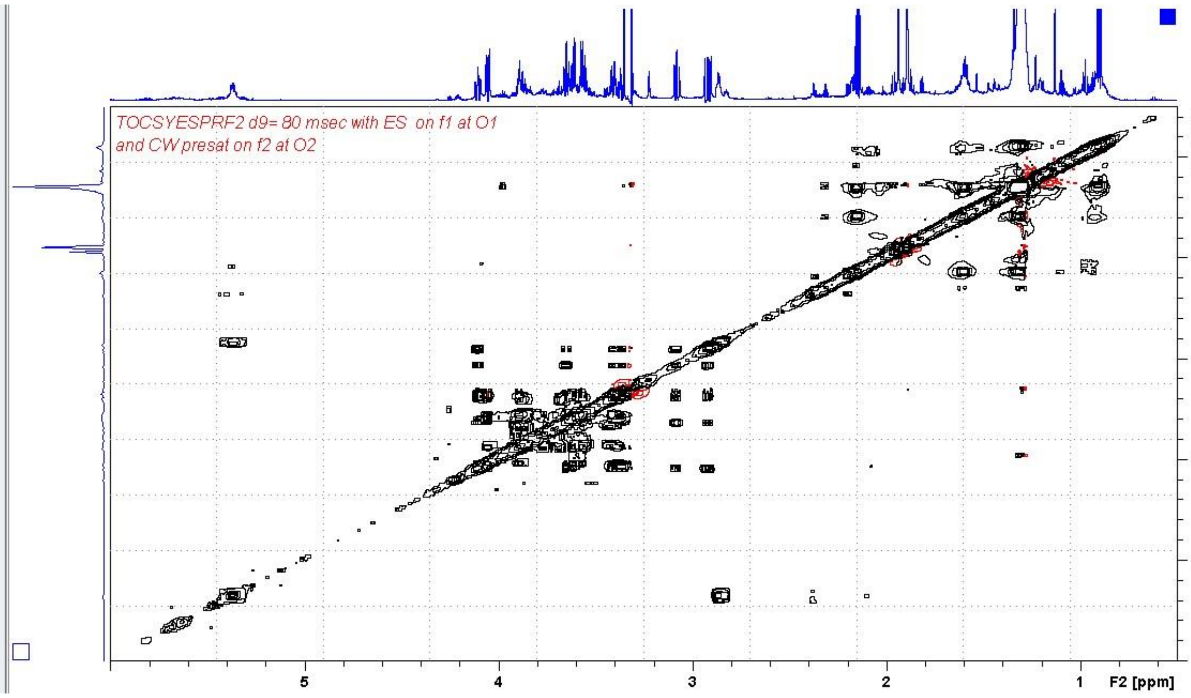

Figure S65. TOCSY NMR spectrum of MTX-6 acquired in CD_3OD at 800 MHz.

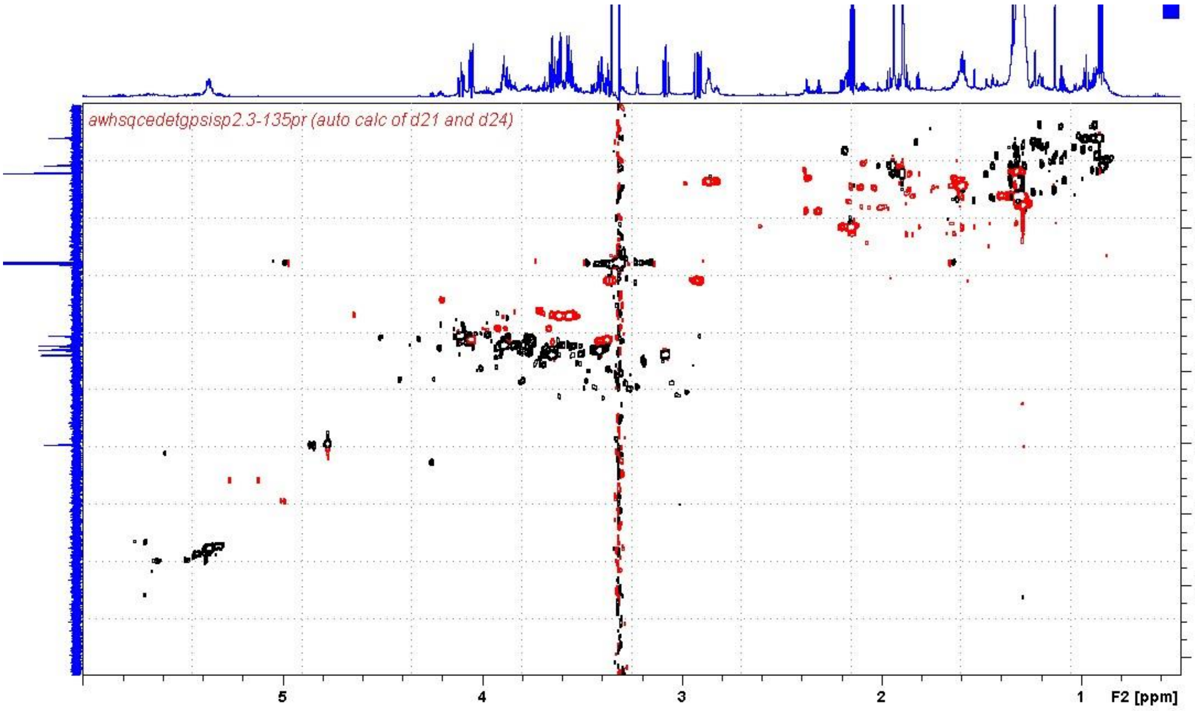

Figure S66. HSQC NMR spectrum of MTX-6 acquired in CD_3OD at 800 MHz.

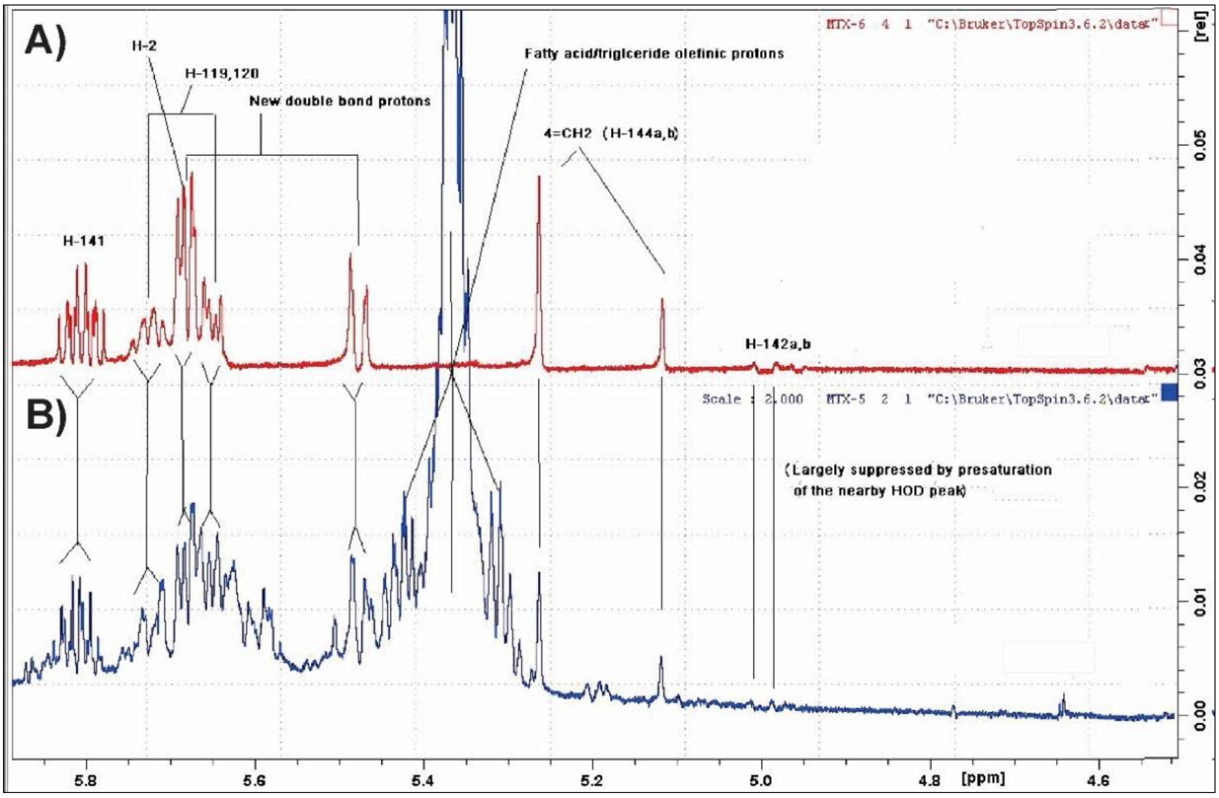

Figure S67. Stacked ^{1}H NMR spectra (4.6–5.8 ppm) of (**A**) MTX-7 and (**B**) MTX-6 acquired in CD_3OD at 800 MHz showing the five sets of olefinic proton signals; H-2, 4=CH_2 (H-$144_{a,b}$), H-119 and H-120 (ring B'), H-141 and H-$142_{a,b}$.

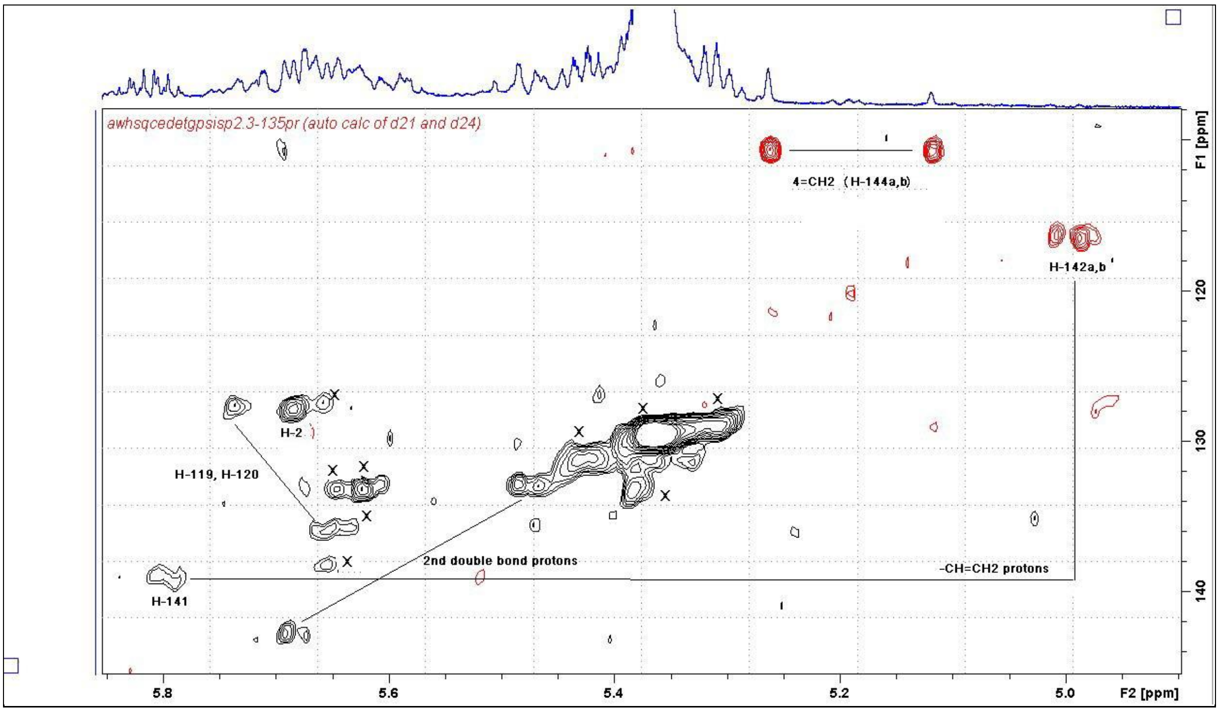

Figure S68. Expansion of the HSQC spectrum (^{1}H: 4.9–6.0 ppm; ^{13}C: 100–150 ppm) of MTX-6 acquired in CD_3OD at 800 MHz showing the five individual or pairs of olefinic protons, with assignments based on those defined for MTX-7 (Figure S52).

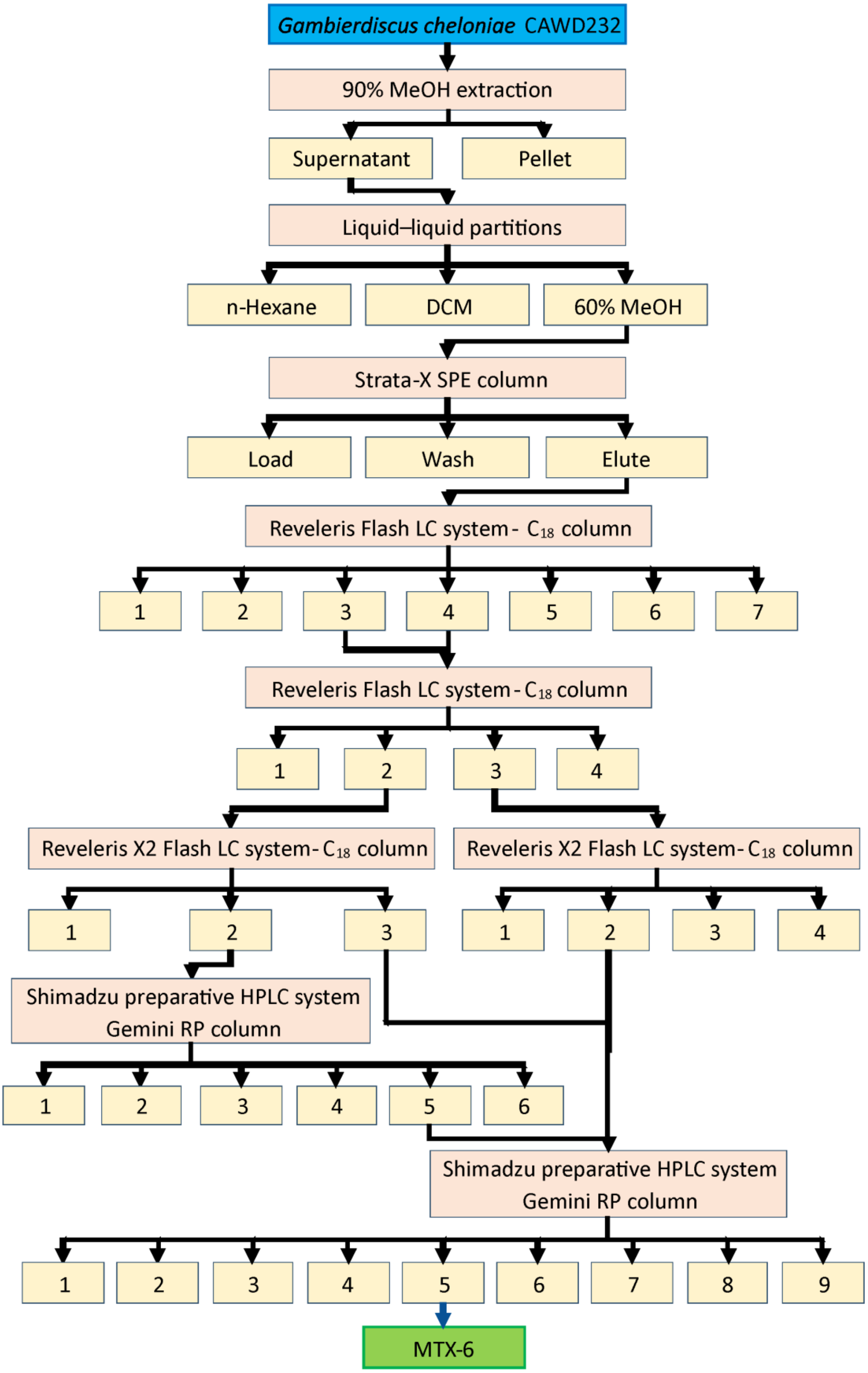

Figure S69. Purification scheme of maitotoxin-6.

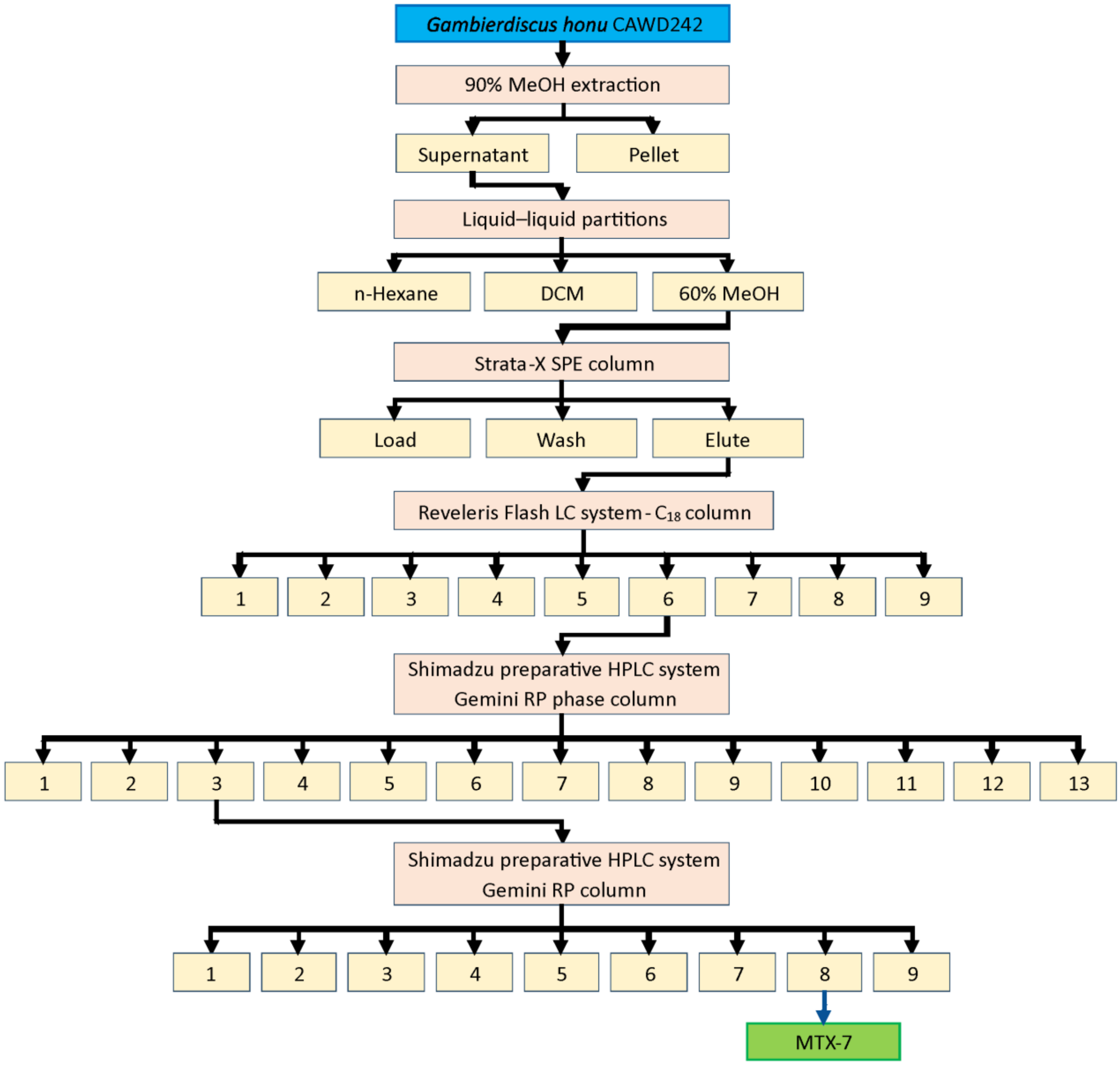

Figure S70. Purification scheme of maitotoxin-7.

Table S6. An inclusion list of the observed periodate oxidation fragment ions for MTX-1, MTX-6 and MTX-7 that were used for the CID experiments.

**Positive ionization**	**Negative ionization**
803.4229 (30 eV CE)	971.4180
903.4059 (30 eV CE)	955.4227
823.4488 (30 eV CE)	1,179.5278
2,316.0703	1,156.5243
1,149.5345	1,158.5042
2,440.1934	1,218.5842
1,220.6019	1,629.8208
921.4154 (30 eV CE)	1,611.8109
841.4580 (30 eV CE)	1,647.8316
1,567.8207	1,753.9464
1,585.7346	1,613.8260
1,709.9563	1,595.8173
1,551.8265	1,631.8375
1,569.8429	1,709.8216
1,665.9309	1,727.9313

MTX = Maitotoxin.

References

1. Murata, M., Iwashita, T., Yokoyama, A., Sasaki, M., and Yasumoto, T., Partial structures of maitotoxin, the most potent marine toxin from the dinoflagellate *Gambierdiscus toxicus*. *Journal of the American Chemical Society*, **1992**. 114: p. 6594-6596.
2. Murata, M., Naoki, H., Iwashita, T., Matsunaga, S., Sasaki, M., Yokoyama, A., and Yasumoto, T., Structure of maitotoxin. *Journal of the American Chemical Society*, **1993**. 115: p. 2060-2062.
3. Organisation for Economic Co-operation and Development, OECD Guideline for Testing of Chemicals 425. Acute Oral Toxicity: Up-and-Down-Procedure (UDP). **2006**. Available from: http://www.epa.gov/oppfead1/harmonization/docs/E425guideline.pdf
4. United States Environmental Protection Agency, User documentation for the AOT425StatPgm program. **2002**.
5. Scheuer, P.J., Takahashi, W., Tsutsumi, J., and Yoshida, T., Ciguatoxin: Isolation and chemical nature. *Science*, **1967**. 155: p. 1267-1268.
6. National Research Council Canada, Molecular formula calculator. 1.01. Available from: https://metrology.shinyapps.io/molecular-formula-calculator/
